# Predicting the Mechanical Properties of Supramolecular Gels

**DOI:** 10.1002/adma.202415031

**Published:** 2025-01-09

**Authors:** Jack D. Simpson, Lisa Thomson, Christopher M. Woodley, Chloe M. Wallace, Bart Dietrich, Alex S. Loch, Dave J. Adams, Neil G. Berry

**Affiliations:** ^1^ Department of Chemistry University of Liverpool Liverpool L69 7ZD UK; ^2^ School of Chemistry University of Glasgow Glasgow G12 8QQ UK

**Keywords:** gels, machine learning, mechanical properties

## Abstract

The prediction of gelation is an important target, yet current models do not predict any post‐gel properties. Gels can be formed through the self‐assembly of many molecules, but close analogs often do not form gels. There has been success using a number of computational approaches to understand and predict gelation from molecular structures. However, these approaches focus on whether or not a gel will form, not on the properties of the resulting gels. Critically, it is the properties of the gels that are important for a specific application, not simply whether a gel will be formed. Supramolecular gels are often kinetically trapped, meaning that predicting gel properties is inherently a difficult challenge. Here, the first successful a priori prediction of gel properties for such self‐assembled, supramolecular systems is reported.

## Graphical abstract

The prediction of gelation is an important target, yet current models do not predict any post‐gel properties. Critically, it is the properties of the gels that are important for a specific application. Supramolecular gels are often kinetically trapped, meaning that predicting gel properties is inherently a difficult challenge. Here, the first successful a priori prediction of gel properties for such self‐assembled, supramolecular systems is reported.

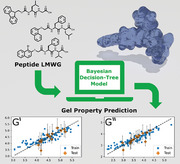

## 1 Introduction

Gels are important soft materials with applications in many areas from food, medicine, and drug delivery to soft robotics, water remediation, sensing, and optoelectronics. Gels can be prepared in many ways. One effective method uses low molecular weight gelators (LMWGs), molecules that self‐assemble to form fibrous structures that entangle to form a network that immobilizes the solvent. Many molecules are effective LMWGs, encompassing a significant diversity of chemical structures.^[^ ^1^, ^2^, ^3^ ^]^

One key issue is that most LMWGs are discovered by accident or through tedious synthetic iteration of known LMWG scaffolds. Given this and other difficulties in generating new gels, work has focused on accelerating discovery by using solvent models and computational approaches. The most common approach to rationalizing gelation ability is to correlate solubility parameters with the gelation state.^[^ ^4^, ^5^ ^]^ Other groups have utilized molecular dynamics (MD) simulations^[^ ^6^, ^7^, ^8^, ^9^, ^10^ ^]^ or density functional theory (DFT) calculations^[^ ^10^, ^11^ ^]^ to investigate the packing of supramolecular gels. For example, Tuttle and Ulijn's group have used coarse‐grained MD approaches to screen the self‐assembly abilities of 8000 tripeptides.^[^ ^7^ ^]^ More recently, MD simulations have been used in combination with machine learning (ML) to guide the design and selection of self‐assembling peptides.^[^ ^8^, ^9^ ^]^ Other work by Zheng and Shi's group has used a generative transformer model in combination with a ML classifier model to generate and virtually screen hydrogel candidate molecules.^[^ ^12^ ^]^

Although work has been published describing predictive models designed specifically for the prediction of gelation state,^[^ ^13^ ^]^ there are few examples using ML directly from Simplified Molecular‐Input Line‐Entry System (SMILES) strings. In the first such report, we successfully utilized physicochemical and fingerprint descriptors to build models to accurately predict the gelation state of di‐ and tri‐peptides in water.^[^ ^14^ ^]^ Other models have since been published that also use physicochemical descriptors to predict the gelation state of peptides.^[^ ^15^, ^16^, ^17^, ^18^ ^]^ However, none of these approaches have provided definitive design rules, which results in ambiguity and reproducibility issues.

All the above focus on predicting whether a gel will be formed with no comment as to the properties of the gels.^[^ ^19^ ^]^ Our previous work predicted the likelihood of a gel being formed, but the resulting gels had a significant variation in their mechanical properties; ability to reheal after shear and transparency, for example. Depending on the application for which the gels are intended, different properties will be required, which necessitates an accurate model to predict these properties. For example, in cell culturing applications, gel stiffness is known to control stem cell differentiation, and therefore specific storage moduli are required depending on the target outcome.^[^ ^20^, ^21^, ^22^, ^23^ ^]^ For applications that utilize imaging of the gels, or prementioned stem cells, high transparency is desirable.^[^ ^24^ ^]^ Hence, it is not sufficient to have an effective prediction as to whether a gel is formed, but an understanding of what the properties of the resulting gels will be is critical to their targeted design.

Mechanical gel properties are quantified by measurement of the storage modulus (*G*ʹ)—how much energy the material stores during deformation, and the loss modulus (*G*ʹʹ)—the measure of the resistance to flow, or the viscous nature, of the gel. Drozdov et al., derived an equation for the storage and loss moduli in polymer gels based on four parameters.^[^ ^25^ ^]^ However, at present, no models exist to predict the rheology of supramolecular gels from a SMILES string; where such a model that can accurately predict the rheological properties of gels would allow for the targeted synthesis of gels for a desired application.

Predicting gel properties is inherently a difficult challenge. Many low molecular weight gelator‐based gels are kinetically trapped materials. They are prepared by finding a set of conditions under which the LMWGs are soluble or dispersed as a micellar phase. Gelation is then triggered, reducing the solubility of the LMWG. This reduction in solubility leads to the formation of 1D structures, such as fibers, that entangle and crosslink in different ways that are typically dependent on how the gel is formed; therefore, the method and process of gel formation can affect the gel's properties. Here, we present models to predict the storage and loss moduli of di‐ and tri‐peptides, leveraging Bayesian learning to evaluate the inherent uncertainty due to the use of small‐data and hence the first example of successful a priori prediction of gel properties for such supramolecular systems.

## 2 Results and Discussion

### 2.1 Gelation

**Figure 1:**
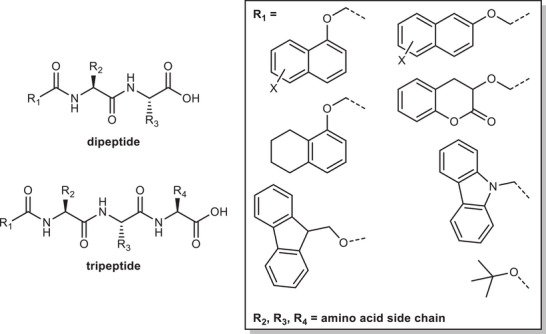
Generic structure of dipeptide and tripeptide LMWGs and the left‐hand‐side substituents used through this work (R1) which were synthesized and rheologically tested.

Gels were prepared from a library of functionalized peptides (see ESI for synthesis and characterization data; generic structures are shown in **Figure** **1**). As stated previously, the properties of gels formed from such LMWGs are highly sensitive to the method of gel formation and the process history. As such, we used well‐established protocols to ensure reproducible materials were formed, with all gels being prepared using a slow pH trigger with a final pH of ≈3.^[^ ^26^ ^]^ These gels are still kinetically trapped, but using a slow pH change removes the issues of mixing and processing the outcome, allowing us to prepare gels with reproducible properties. This is necessary to build effective models as a means of removing most issues of processing,^[^ ^26^, ^27^ ^]^ but we acknowledge that this means that the methodology described here will always by default for such systems be limited to a specific gelation method.

The rheological properties were determined directly in the gelation vials ensuring that no damage occurred on loading. Each system was measured in triplicate to ensure reproducibility. The values of *G*′ and *G*″ presented are at strains within the linear viscoelastic region. There are 33 molecules in the dataset of 90 points (28 molecules tested at gel concentrations of 2.5, 5, and 10 mg mL^−1^, 1 molecule tested at gel concentrations of 5 and 10 mg mL^−1^, 1 molecule tested at a gel concentration of 2.5 mg mL^−1^, and 3 molecules tested at a gel concentration of 5 mg mL^−1^). We calculated pairwise Tanimoto distances (a metric of dissimilarity commonly used for comparing molecular fingerprints^[^ ^28^ ^]^) between all LMWG and found the mean distance was 0.621 ± 0.17 indicating on average compounds are moderately dissimilar to each other.

### 2.2 Dataset Construction and Visualization

**Figure 2:**
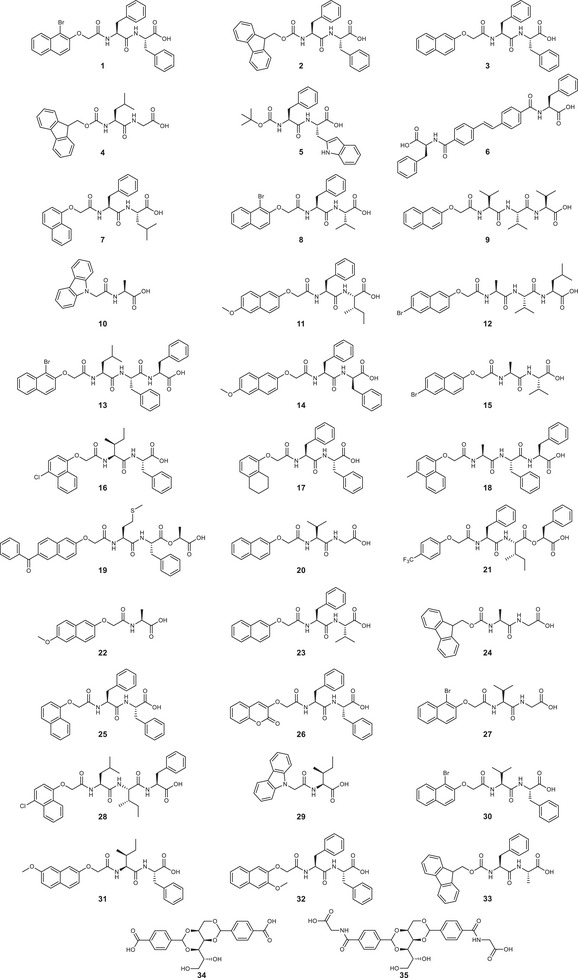
Structures of the LMWGs used to build predictive models in this work.

In any data set, there will be a varying dependence of model performance on dataset composition. Therefore, we have tuned and evaluated multiple models using multiple unique data splits. This helps us determine the generalisability of our approach. For each model, the dataset was split into a test set representing ≈15% of all molecules and a training set composed of 85%. The split was semi‐stratified to ensure that rheology values in the training set span the range of the entire dataset. We have also included concentration partners with any split. We first placed molecules with the highest and lowest *G*′ and *G*″ values into the training set at all measured concentrations. The remaining molecules were divided into six‐folds, each containing four or five molecules. We used these folds to build six datasets such that each fold acted as the test set exactly once, and the remaining five folds were added to the training set for model building and cross‐validated hyperparameter tuning. This process is summarized in Figure S2 (Supporting Information). The molecules used to build models used in this work are shown in **Figure** **2**.

**Figure 3:**
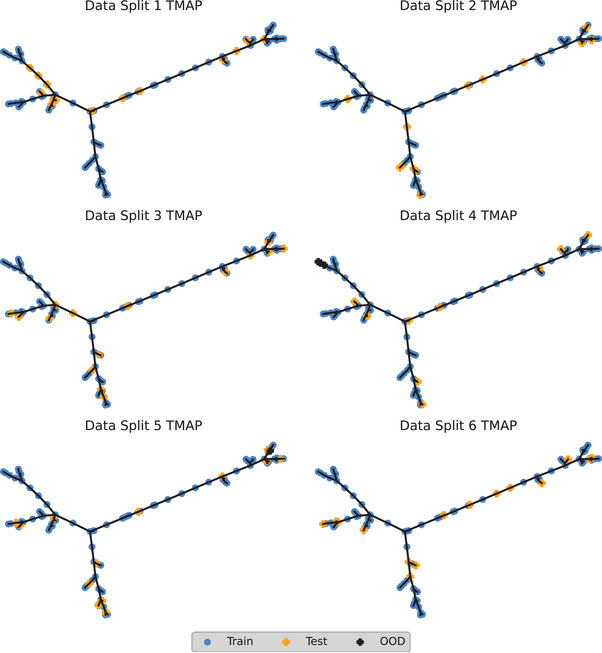
TMAP representation of compounds in training (blue circles 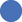) and test (orange cross 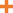) sets for each of the 6 data splits. Out Of applicability Domain (OOD) compounds are shown as black crosses (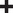) The distance between points represents similarity between the descriptor sets.

We used a tree manifold approximation and projection (TMAP) representation (**Figure** **3**) to visualize the relationship between the training set, testing set, and validation set points based on their descriptors. TMAP is a method of dimensionality reduction which represents clusters of similar points as branches, where distances between the points indicate similarity.^[^ ^29^ ^]^ Visually, the distribution of points in the TMAP space indicates that most test set examples are “close to” or between training set points; therefore, for most points, prediction is considered to be interpolation. Test points at the termini of branches indicate “isolation” from training set points, suggesting rheological predictions on these points might perform poorly. We observe differences in the distribution of test set examples across each unique data splits—discussed in Note S1 (Supporting Information).

For each model we define an applicability domain (AD) based on descriptor values and molecular similarity. Using our AD, we identified four out‐of‐domain points (denoted 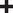 in Figure 3)—LWMG **6** in data split 4, and LMWG **33** in data split 5 at a gel concentration of 10 mg mL^−1^. The former was flagged at all concentrations due its minimum Tanimoto distance to any training set molecule exceeding our defined threshold. All three instances of this molecule are at a branch terminus, implying predictions for this point may be extrapolation—as molecule **6** is the only example of a stilbene LMWG in our dataset, identification as an outlier when not in the training set is rational. The latter AD outlier was flagged due to range checking of individual descriptor values. The flagged point exists within a sub‐branch and is very close to adjacent training set examples, and no structural feature of **33** is clearly out‐of‐domain. The remaining instances of test LMWGs at branch termini were not flagged by our AD approach for any data split.

We chose to use the Bayesian Additive Regression Tree (BART) algorithm for ML for two reasons. First, predictions made using BART provide quantification of uncertainty in the prediction.^[^ ^30^, ^31^ ^]^ Second, the BART algorithm inherently prevents overfitting by applying constraints to the structure and number of decision trees used in its construction; this is an important consideration when utilizing small datasets such as ours.^[^ ^30^, ^31^ ^]^ BART requires that the endpoint of the model (*G*′ and *G*″ in this work) must be normally distributed.^[^ ^32^ ^]^ Figure S3A,B (Supporting Information) show the distribution of *G*′ and *G*″ values in our dataset on a logarithmic scale as an approximate normal distribution. This is supported by Shapiro–Wilk tests and quantile–quantile plots which both confirmed the data is normally distributed (Note S1, Supporting Information).

### 2.3 Evaluation of BART Models

**Figure 4:**
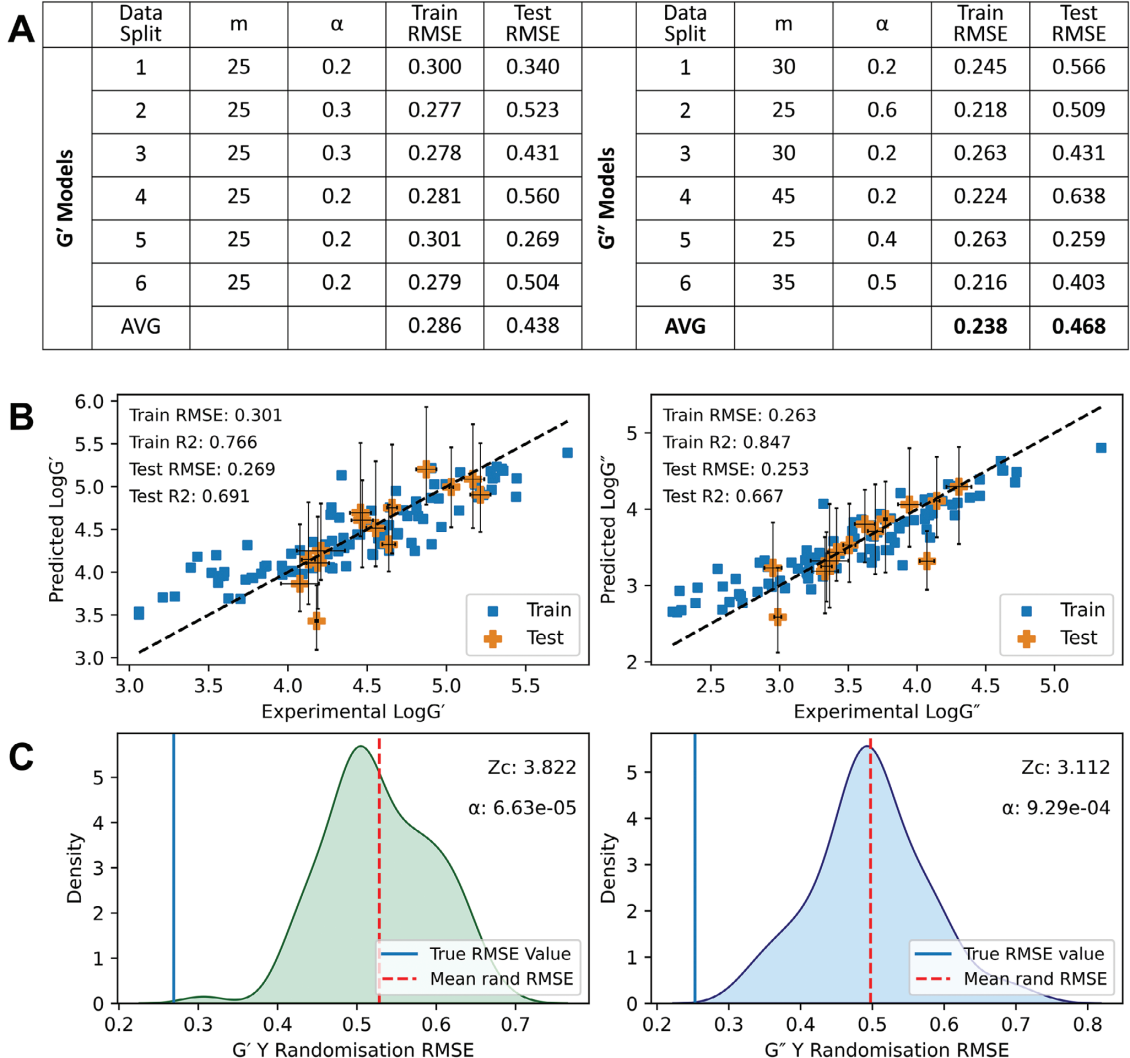
Summary of model performance of our BART models. A) Table summarising tuned hyperparameters and testing set performance metrics for each data split. B) Scatterplots of measured rheological values (*x*‐axis) plotted against BART‐predicted *G*′ and *G*″ (*y*‐axis) of the best‐performing data split. The line *y* = *x* is plotted as a dashed black line for reference. For the test set examples, the experimental error is indicated by horizontal error bars, and the 89% Bayesian credible interval is indicated by vertical error bars. C) KDE plots of the testing set RMSE scores resulting from the y randomization approach of the best‐performing data split 5. The RMSE value for true data is shown as a vertical, blue line. The mean RMSE value for randomized models is shown as a vertical, dashed, and red line. Plots of predictions and Y‐randomisation test KDE plots are included in the Supporting Information.

To investigate the dependence of model performance on dataset composition, we tuned and evaluated 6 models utilizing all compounds as the testing set exactly once. BART is a relatively new algorithm, so no consensus exists on the necessity to tune hyperparameters—the hyperparameters are the number of trees (m) and the probability of a node being terminal (or depth of tree, α) (see Note S2, Supporting Information). We used a five‐fold cross validation (5xCV) approach to screen hyperparameters m ∈ {10,15,.,50} and α ∈ {0.1,0.2,…,0.9}. To validate our data‐splitting and hyperparameter tuning approach we also evaluated alternative splitting approaches (Note S3, Supporting Information). We observed clear underfitting of training data with low values of m and α and believed that training data was being overfit with high values of alpha. We therefore restricted choice of hyperparameters to m ≥ 25 and α ∈ {0.2,0.3,…,0.8} for the final models, chosen by the lowest average RMSE value derived from cross‐validation of the training set examples. The results of the hyperparameter screening are shown in Table S3 and Figures S4 and S5 (Supporting Information). Optimized parameters are summarised in **Figure** **4**A.

For each *G*′ model, the optimal hyperparameter m was determined to be 25 while chosen values for α varied between 0.2 and 0.3. This indicates a trend for fewer, deeper trees affording more generalizable models for *G*′. For *G*″, a wider range of m and α values were selected. Values of m varied from 25 in data split 2 to 45 in data split 4. Similarly, chosen values of α varied between 0.2 and 0.6—like *G*′ a preference for deeper trees is observed.

Figures S6 and S7 (Supporting Information) show scatterplots of predicted versus experimental values for *G*′ and *G*″ for all models, the best‐performing model built on data split 5 is shown in Figure 4B. We defined a good model as having an R^2^ value of > 0.6 and a low root‐mean‐squared error (RMSE) on the testing set. Given that our data roughly covers 2.6 log units (*G*′: 3.2–5.8, *G*″: 2.2–4.7), we consider RMSE of 10% of this range (0.3 or below) as low. Training set points for all *G*′ and *G*″ models possessed R^2^ values greater than our threshold of 0.6 for a good model and most possessed low RMSE values; the best‐performing data split 4 has a training set RMSE of 0.301 however this is very close to our defined threshold value and can be considered adequate.

More variation is seen amongst testing set performance with RMSE values ranging from 0.269 to 0.589 for *G*′ and 0.253 to 0.662 for *G*″ highlighting dependence on model performance on dataset composition. Despite spanning a range of values, the average RMSE values across all splits were 0.438 and 0.468 respectively indicating moderate predictive performance and therefore some generalisability in each split. These average RMSE values compare favorably to the standard deviation of the *G*′ and *G*″ datasets (0.605 and 0.644, respectively) further supporting the predictive performance of these models. Owing to the smaller size of the testing set, R^2^ is likely an unreliable metric for comparison, however, it is noteworthy that the best‐performing data split five afforded good R^2^ values by our metrics and three of six folds for *G*′ and *G*″ afforded R^2^ values above 0.4.

Using BART, predictions are the mean value of a defined number of draws from the posterior function. We can therefore derive an 89% Bayesian credible interval (CI) for each point as a measure of uncertainty. We define a prediction as poor when the experimental value falls outside the CI. For *G*′ and *G*″ models built on each data split, the majority of points’ CI encompass experimental values, indicating reasonable predictive power. In training set performance, most non‐overlapping CI points correspond to low measured *G*′ and *G*″ values indicating poorer model performance on weaker gels. The fit of *G*″ is demonstrably better than *G*′ for all models with fewer non‐overlapping points; this is likely due to the selection of hyperparameter sets with larger m and α allowing closer fitting of training set values. The poorest performing *G*′ test sets, data splits 2 and 4, had the greatest number of non‐overlapping points while the best performing data split, 5, had just one non‐overlapping point. In *G*″, data splits 4 and 6 had four non‐overlapping points despite data split 6 possessing a moderate test set RMSE of 0.408.

We used y‐randomization tests to investigate whether the performance of our models could not be achieved using a random distribution of rheological properties. The density plots (Figure 4C) show the distribution of testing set RMSE values for 100 models trained on randomized rheological datasets for the best‐performing models (data split 5). The true RMSE values for data split 5 *G*′ and *G*″ models appear to be derived from a distinct distribution compared to the randomized models. We confirmed this by calculating Z scores (3.82 and 3.11 for *G*′ and *G*″, respectively) and corresponding p values (6.6e^−5^ and 9.3e^−4^, respectively), indicating a very low probability of the models’ true RMSE values belong to the same distribution as the randomised models.^[^ ^33^ ^]^ Y randomisation of all data splits (Figures S8 and S9, Supporting Information) showed similar results for all training set performances and most testing set performances—only the *G*′ test set for data split 2 and *G*″ test sets for data splits 1 and 4 performed poorly in Y randomization studies (Note S4, Supporting Information). Overall, these results suggest moderate to good generalisability of our trained models for most data splits.

Our AD flagged compound **33** in data split 5. Prediction of compound **33** in data split 5 possess low residuals in *G*′ and *G*″ models (0.27 and 0.33, respectively) and inclusion makes negligible difference to model metrics for *G*′ and a increases RMSE for *G*″ (Figure S10, Supporting Information). Similarly, the stilbene LMWG **6** in data split 4 was identified out of domain but still predicted with comparable residuals to other testing set points. TMAP visualization of data splits 4 and 5 scaled by residual value (Figure S11, Supporting Information) shows that the most poorly predicted points are not isolated from training set points. Therefore, simple domain checking or TMAP representation is not sufficient to flag molecules that may be poorly predicted. Similarly, the visual imbalance of test set distribution on the tree manifold for different splits does not appear to predict overall model‐building performance—though the top‐performing data split 5 has a good distribution. While our model shows good predictive power for test set molecules, with low RMSEs, it is important to consider our models’ inherent limitations. Our models take a simplified approach to predicting a complicated, multi‐stage supramolecular process. By using SMILES strings to generate simple physicochemical and 2D molecular descriptors, our models are not exposed to any characterization of intermolecular interactions between individual LMWGs. Therefore, our models must infer the effects of the presence and values of descriptors on gelation from the rheological endpoints. Other work has trained ML models on data derived from MD‐ and DFT‐based calculations, which capture the aggregation characteristics of self‐assembling peptides,^[^ ^8^, ^9^ ^]^ at a significantly greater computational cost.

We again note that these properties are inherently difficult to predict. Kinetic traps during the process of gelation can lead to many local minima. For this class of compound, it is not that a certain compound gives gels with specific *G*′ and *G*″, but rather a specific compound gives a gel with a specific *G*′ and *G*″ depending on the process of gelation. The data presented here shows that our models can effectively predict properties as long as one the same process is used for training and testing sets—we believe this is the first literature example of this.

### 2.4 Model Interpretation Using SHAP Values

We used model interpretation to understand how our models arrive at their predictions and to verify that the inferences made by the models are founded in rational gel design. The SHapley Additive exPlanations (SHAP) methodology uses coalitional game theory and local surrogate models to assign importance values to individual features in order to arrive at a given prediction.^[^ ^34^, ^35^ ^]^ For quantitative structure–property relationship (QSPR) models, the SHAP values assigned to physicochemical or fingerprint descriptors describe how a model assigns a particular endpoint to a molecule; this has been demonstrated in several published QSPR studies.^[^ ^36^, ^37^, ^38^ ^]^ As described previously, the models were built using physicochemical descriptors (number of rings), and Extended Connectivity and Functional Class FingerPrint descriptors (ECFPs and FCFPs, respectively).^[^ ^39^ ^]^ These descriptors were considered in the SHAP analysis.

**Figure 5:**
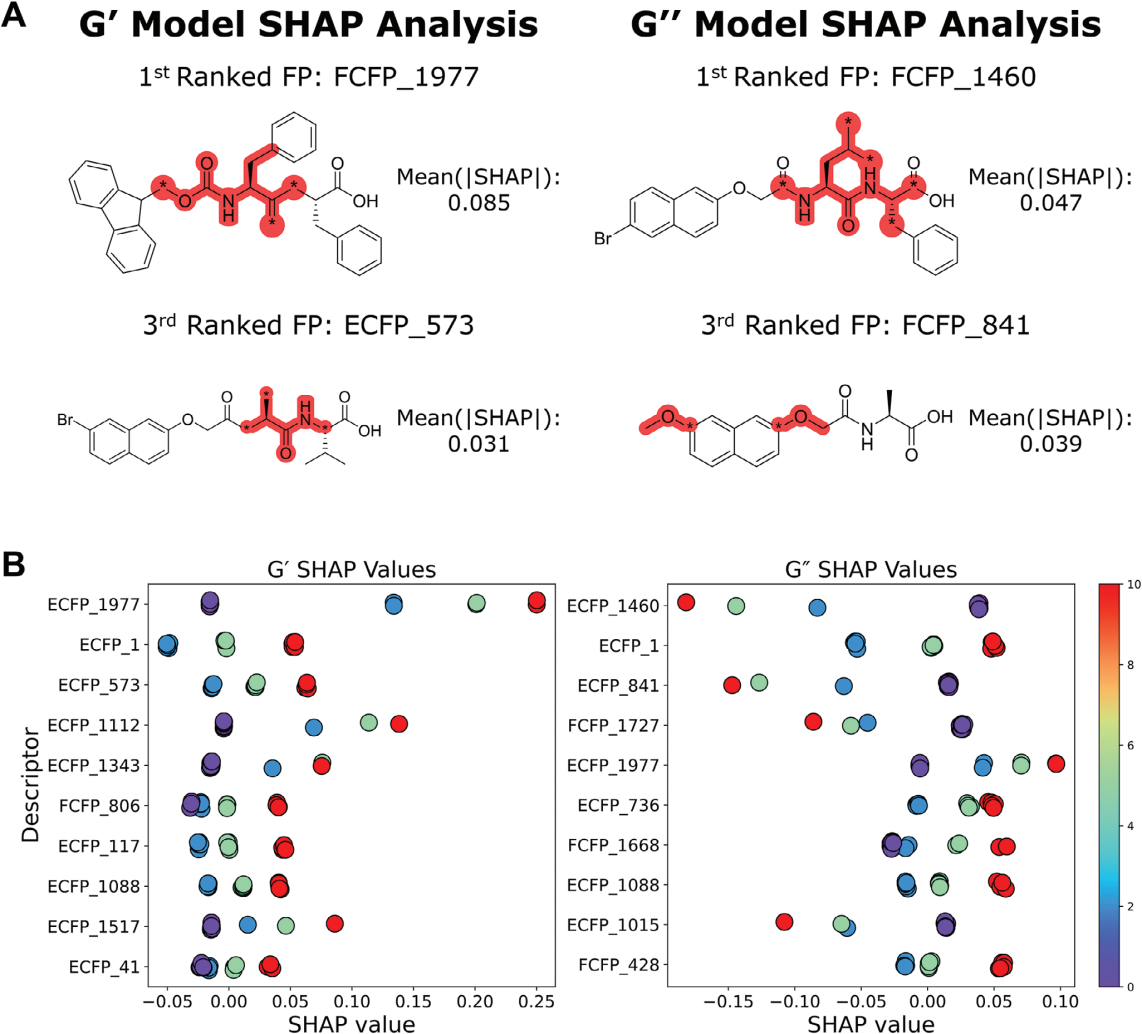
SHAP analysis of the predictions on unseen compounds by our BART models. A) Substructures corresponding to the first and third highest‐ranking fingerprints identified by SHAP analysis on the *G*′ and *G*″ BART models. Fingerprint descriptors are displayed in the context of a molecule containing the fingerprint with asterisks representing the connecting atom in the molecule. B) Categorical scatterplots displaying SHAP values for individual predictions of the combined validation and test sets. Points are coloured proportional to concentration and/or fingerprint presence.

**Figure** **5** summarises SHAP analysis for the *G*′ and *G*″ models on the test set from data split 5. The training set was excluded to assess predictions on unseen molecules. The structures of the two most important fingerprint descriptors for each model are shown in Figure 5A in the context of a molecule containing this fingerprint alongside their mean absolute SHAP values—the average effect that fingerprint has on the model outcome. The structures of other highly ranked fingerprint descriptors are shown in Figure S12 (Supporting Information). Figure 5B shows SHAP values assigned to the 12 most important descriptors for predictions ordered by their mean‐absolute SHAP value; for both *G*′ and *G*″ cases these include nine molecular fingerprint descriptors and the number of rings. Each marker represents the SHAP value (*X*‐axis) for a given descriptor (*Y*‐axis) for a single prediction. Markers for fingerprint descriptors are colored for interpretation—if a molecular fingerprint is present the marker is coloured by concentration (2.5, 5, 10 mg mL^−1^, on the color‐bar) and coloured purple if absent (0 on the color‐bar). Markers for a number of rings are colored proportional to the number of rings and concentration. The relationship between marker‐colour and SHAP value qualitatively describe the effects of descriptors on the LMWG's rheological properties. A full list of fingerprint descriptors used to build these models in SMARTS format and mean‐absolute SHAP values is given in Table S4 (Supporting Information).

Aromatic fingerprints, corresponding to either phenylalanine or the naphthalene core, are seen amongst the most important fingerprints including ECFP_1977, FCFP_806 ECFP_1112 and ECFP_1088, from the *G*′ model, and ECFP_1088 and ECFP_1977, from the *G*″ model—some also correspond to leucine. For most of these fingerprints we see that when these fingerprints are present the SHAP value is higher, suggesting that inclusion increases gel strength. At higher concentrations we also see higher SHAP values echoing the observed dependence of gel strength on concentration. The positive impact of these fingerprints can be attributed to aromatic rings promoting π–π stacking in gel formation, resulting in stronger gels,^[^ ^40^, ^41^ ^]^ or the steric bulk of leucine promoting aggregation. π–π interactions in biaryl‐containing LMWGs and vanadium metallogels have been previously characterised by NMR, FT‐IR and X‐ray methods.^[^ ^40^, ^41^ ^]^ Phenylalanine in LMWGs has been widely investigated with work explicitly referencing the potential for π–π interactions.^[^ ^29^, ^42^ ^]^

In contrast, for the third most important *G*″ descriptor, ECFP_841 (a methyl ether fragment, matched in methoxynapthyl), we see that the SHAP value decreases if this fingerprint is present and a decrease in SHAP value with increasing concentration. This implies that the methoxy group, through steric or electronic effects, disrupts gelation and reduces the *G*″ value. ECFP_841 is seen to have minimal effect in the *G*′ model. Unfavourable effects of methoxynaphthalene on gel strength has not been reported in the literature so could represent a new insight drawn from these models.

To investigate this, we synthesised and characterised a further methoxynapthyl containing (7MeO2NapFF, **36**, Figure S27, Table S8, Supporting Information). In comparison to its napthalene analogue, rheological properties were lower; the difference was greater for *G*″ than in *G*′ in agreement with the higher feature important in *G*″ SHAP analysis. Our *G*′ model predicted near identical values for **36** and **3** reflecting the low importance of this fingerprint in this model; the rank order of data points, however, was correctly predicted. The *G*″ model predicted larger differences in values and correctly identified the rank order of data points.

Valine or isoleucine fingerprints are among the most important descriptors. ECFP_573, ECFP_1517 and ECFP_1343 for the *G*′ model, and FCFP_1668 for the *G*″ model, have a positive correlation between SHAP and descriptor values. In contrast ECFP_1460, FCFP_1727 and ECFP_1015 in the *G*″ model show a negative correlation. This suggests a nuanced effect of the specific steric bulk of valine or isoleucine on gelation, sufficient to reduce *G*″ of the final gel while promoting *G*′. ECFP_1, corresponding to the alpha carbon in any amino acid excluding glycine is also included as the second most important descriptors in *G*′ and *G*″ models respectively. Other fingerprints corresponding to the amide core, ECFP_117, ECFP_41 and FCFP_806 also correspond to fragments of the amide backbone of these LMWG. While these descriptor are broadly applicable, this may suggest non‐amide or glycine containing LMWG, such as the stilbene **6** and 1,3:2,4‐dibenzylidene sorbitols **34** and **35**, produce gels with lower *G*′ and *G*″.

In both the *G*′ and *G*″ models, the number of rings within the LMWG is highly ranked among descriptors (14th and 15th for *G*′ and *G*″, respectively). This is likely due to the majority of rings in the set of molecules being aromatic, which enables π–π interactions between LMWGs;^[^ ^40^, ^41^ ^]^ this is supported by the positive correlation seen between the number of rings and SHAP value.

Overall, the SHAP methodology has enabled us to interrogate our predictive models to observe which features of LMWGs improve or adversely affect *G*′ and *G*″ rheological properties. By visualising the relationship between SHAP value and descriptor value, it affords us a human‐interpretable understanding of model outcomes. Furthermore, the key observations that aromatic groups and leucine promote stronger gelation is founded in experimental evidence, that aromatic and bulky aliphatic residues promote aggregation.^[^ ^29^, ^40^, ^41^, ^42^ ^]^ While this provides confidence in the predictions drawn from our models, our models also identified the counterintuitive influence of valine and isoleucine on *G*″ not presently understood in the literature, which may direct further investigation.

## 3 Conclusion

We present the first example a priori prediction of rheological properties of peptide‐based gelators using a Bayesian decision tree model to both generate predictions and provide a measurement of uncertainty borne from the use of small‐data by necessity. Models were built and optimized by 5xCV and evaluated using holdout testing datasets, as well as y‐randomization tests. We built, tuned, and evaluated 6 models based on different combinations of data splits to assess the expected dataset dependence due to using small data. The performance of the best data split derived *G*′ and *G*″ meets our good criteria, achieving the predetermined thresholds (R^2^ > 0.6, and RMSE < 0.3) on the testing set, while most other dataset splits showed moderate predictive performance—significantly better than random by y‐randomization test. For such gels, the mechanical properties required depend on the desired application. As a single example, in cell culturing applications, gel stiffness is known to control stem cell differentiation, and therefore different specific storage moduli are required depending on the target outcome^[^ ^20^, ^21^, ^22^, ^23^ ^]^ As such, an a priori prediction of gel properties is invaluable, allowing one to pre‐target which gels will provide the necessary properties as opposed the current requirement to iteratively prepare and test gels until one with the required properties is found.

SHAP methodology allowed us to evaluate the effect of individual descriptors on the rheological properties of the final gels. This revealed that aromatic moieties that engage in π‐stacking and bulky aliphatic amino acid side chains are likely to promote a stronger gel with greater *G*′ and *G*″ values. This also identified valine as counterintuitively modulating gel strength which we posit could be due to the specific steric bulk of the valine being sufficient to disrupt aggregation.

Owing to the complexity of the systems we are predicting for from simple, computationally cheap methods, these results are promising for future iterations of models trained with newly obtained data. We again highlight the difficulty in predicting properties from such kinetically trapped materials and our success here is a major step forward. We move beyond the useful, but limited, simple prediction of gelation to actual material properties, which are critical no matter the application. Our current approach has focussed on the mechanical properties of the gels, but similar approaches could be used for other properties of importance.

## 4 Experimental Section

### Synthesis

The library of gelators is based around di‐ and tri‐peptides, building from the previously reported results.^[^ ^14^ ^]^ All gelators were prepared by standard solution‐based approaches. The synthesis and characterization of new gelators is described in the Supporting Information.

### Gelation Testing

To produce a LMWG solution from which hydrogels can be prepared requires the gelator, one molar equivalent of 0.1 m aqueous NaOH, with respect to the gelator, and water such that the final concentration of LMWG is either 2.5, 5, or 10 mg mL^−1^. Each solution was stirred overnight at room temperature to form a homogeneous solution. The exceptions to this are those LMWGs containing Fmoc protecting groups, which were only stirred for ≈2 h (at which time they were visibly homogeneous) to reduce the risk of Fmoc cleavage. The pH of each solution was then adjusted to pH 10.5 ± 0.1 using a calibrated Hanna FC2020 pH probe. 2 mL of each solution was then pipetted into a 7 mL Sterilin vial containing glucono‐δ‐lactone (GdL). This was stirred briefly with a spatula to dissolve the GdL and then left undisturbed overnight to allow gelation to occur. Samples were inverted the following day to check for gelation. If invertible, gels were then analyzed using rheology. If the sample could not resist inversion, it was concluded that no gel was formed, and rheology was not performed. The final pH was also confirmed.

Strain sweeps were performed using an Anton Paar Physica MCR 301 rheometer using a vane (ST10) geometry and gap of 1.8 mm to measure 2 mL of gel in Sterilin vials. Strain tests were performed in triplicate at 10 rad s^−1^ from 0.01% to 1000% strain at 25 °C. The viscoelastic region was determined and ends at the strain value (%) where Gʹ deviates from linearity, indicating the point at which the gel begins to break, denoted as the break point. The presented data points represent the average of the triplicate, with the error bars as the standard deviation. To obtain *G*ʹ and *G*″ values for the prediction, *G*ʹ and *G*ʺ were averaged up to and including the break point for each gelator. The errors were also averaged to give the overall error in *G*ʹ and *G*ʺ. We used these values as their logarithms to base 10. The range of measured *G* was 1150–580333 Pa, and *G*″was 167–219000 Pa (log *G*' 3.06–5.76, and log *G*″ 2.22–5.34). The average *G*′ values and *G*″ values for di‐ and tripeptides are comparable (*G*′: 4.57 ± 0.51 and 4.42 ± 0.67, *G*″: 3.67 ± 0.59 and 3.38 ± 0.66 for di‐ and tripeptides, respectively).

### QSPR Methods

The molecules described were generated in silico using ChemDraw and converted to SMILES strings. Physicochemical descriptors were calculated using the RDKit module in Python (AlogP and the number of rings),^[^ ^43^ ^]^ and molecular solubility was calculated using the approach form Delaney et al. implemented in Python.^[^ ^44^ ^]^ The RDKit implementation of Morgan fingerprints with and without features (analogous to Extended Connectivity (ECFP) and Functional Class FingerPrints)^[^ ^39^ ^]^ using a radius of two bonds and encoding in 2048 bits was used. As the rheological values were attempted to predict were shown to scale with concentration, we multiply the descriptor values by the experimental concentration^[^ ^45^ ^]^ (Note S4, Supporting Information). These descriptors were used to build the models presented in this work.

Dataset splitting was achieved using the KFold splitting method as implemented in the Scikit‐Learn package as implemented in Python.^[^ ^46^ ^]^ Tree manifold approximation projections (TMAP) were used to visualize our high‐dimensional datasets using the tmap package as implemented in Python.^[^ ^29^ ^]^

The machine learning models were used using the BART algorithm using the PyMC3 module in Python.^[^ ^47^ ^]^ Model performance on the training, and test sets was assessed by coefficient of determination (R^2^) and root‐mean‐squared error (RMSE) between measured and calculated values. A compound in‐range of the applicability domain (AD) was considered if the following criteria were met: 1) the Tanimoto distance between a given molecule's entire ECFP fingerprint bit‐vector and its nearest training‐set neighbor was smaller than the mean Tanimoto distance between all training set molecules, and 2) the value of each individual descriptor, using the feature vectors after removal of correlated and zero variance features, falls within the range of the training set.

Model interpretation was achieved using SHapley Additive exPlanation (SHAP) values via the Shap package in Python (*version 0.40.0)*.^[^ ^34^ ^]^

### Code Availability

The dataset and code used to train the models used in this work and a Google‐Collab implementation of our models are available at https://doi.org/10.5281/zenodo.12795740.

## Conflict of Interest

The authors declare no conflict of interest.

## Supporting information



Supporting Information

## Data Availability

The authors declare that the data supporting the findings of this study are available within the paper and its Supplementary Information files.
