# Supplementary material for: Predicting the Mechanical Properties of Supramolecular Gels
Source: Adv Mater. 2025 Jan 9;37(8):2415031. doi: 10.1002/adma.202415031 (PMC11854865; doi:10.1002/adma.202415031)
Supplement: Supplementary file 1 — Supporting Information [file ADMA-37-2415031-s001.docx]

Supporting Information

Predicting the Mechanical Properties of Supramolecular Gels

Jack D. Simpson, Lisa Thomson, Christopher M. Woodley, Chloe M. Wallace, Bart Dietrich, Alex S. Loch, Dave J. Adams* and Neil G. Berry*

**Experimental**

Chemical shifts (*d*) are reported in parts per million (ppm) to the residual solvent peak (CDCl_3_: 7.26 ppm for ^1^H and 77.0 ppm for ^13^C; DMSO-d_6_: 2.50 ppm for ^1^H and 39.52 ppm for ^13^C). Coupling constants (*J*) are given to the nearest 1.0 Hertz. Peak multiplicities are reported as singlet (s), doublet (d), triplet (t), quartet (q), octet (oct.), multiplet (m), and/or broad (b). Peak assignments are reported as: H_Ar_ = aromatic H, H_indole_ = indolyl H or H_Nap_ = naphthalenyl H.

**Gelators**

The gelators used to build models in this work are shown in **Figure S1**.

**Figure S1.** LMWGs used in this work.

Compound **1** was synthesised as described below.

Compound **2** was synthesised as described elsewhere^[1]^.

Compound **3** was synthesised as described elsewhere^[2]^.

Compound **4** was synthesised as described elsewhere^[3]^.

Compound **5** was synthesised as described below.

Compound **6** was synthesised as described elsewhere^[4]^.

Compound **7** was synthesised as described below.

Compound **8** was synthesised as described below.

Compound **9** was synthesised as described elsewhere^[5]^.

Compound **10** was synthesised as described elsewhere^[6]^.

Compound **11** was synthesised as described below.

Compound **12** was synthesised as described below.

Compound **13** was synthesised as described below.

Compound **14** was synthesised as described elsewhere^[2]^.

Compound **15** was synthesised as described elsewhere^[2]^.

Compound **16** was synthesised as described below.

Compound **17** was synthesised as described elsewhere^[7]^.

Compound **18** was synthesised as described below.

Compound **19** was synthesised as described below.

Compound **20** was synthesised as described elsewhere^[2]^.

Compound **21** was synthesised as described below.

Compound **22** was synthesised as described elsewhere^[8]^.

Compound **23** was synthesised as described elsewhere^[2]^.

Compound **24** was synthesised as described elsewhere^[3]^.

Compound **25** was synthesised as described elsewhere^[2]^.

Compound **26** was synthesised as described elsewhere^[9]^.

Compound **27** was synthesised as described elsewhere^[10]^.

Compound **28** was synthesised as described below.

Compound **29** was synthesised as described elsewhere^[11]^.

Compound **30** was synthesised as described below.

Compound **31** was synthesised as described below.

Compound **32** was synthesised as described elsewhere^[11]^.

Compound **33** was synthesised as described elsewhere^[3]^.

Compound **34** was synthesised as described below.

Compound **35** was synthesised as described below.

Compound **36** was synthesised as described elsewhere.^[12]^

Compound **1** was prepared by the coupling of 1-bromo-2-naphthoxy acetic acid (prepared as described elsewhere^11^) with FFOMe.TFA (prepared as described elsewhere for the ethoxy protected FF^12^) to give 1Br2NapFFOMe using our established methods. 1Br2NapFFOMe was deprotected using LiOH in THF/water following our usual methods to give compound **1**.

**BocFFOMe**. *d*_H_ (400 MHz, DMSO-d_6_) 8.39 (0.2H, bd, *J* 8, NH), 8.33 (0.8H, d, *J* 8, NH), 7.32−7.14 (10H, m, H_Ar_), 6.85 (0.9H, d, *J* 9, NH), 6.41 (0.1H, bd, *J* 8, NH), 4.51 (1H, q, *J* 7, CH), 4.17 (0.8H, dt, *J* 10, 4, CH), 4.12−4.02 (0.2H, m, CH), 3.58 (3H, s, O-CH_3_), 3.05 (1H, dd, *J* 14, 6, CH_2_), 2.96 (1H, dd, *J* 14, 8.5, CH_2_), 2.88 (1H, dd, *J* 14, 4, CH_2_), 2.67 (1H, dd, *J* 14, 10, CH_2_), 1.28 (7.5H, s, CH_3_), 1.14 (1.5H, s, CH_3_). *d*_C_ (100 MHz, DMSO-d_6_) 171.83, 171.80, 155.1, 138.0, 137.0, 129.1, 129.1, 128.2, 128.0, 126.6, 126.1, 78.0, 55.5, 53.5, 51.8, 37.4, 36.7, 28.1, 27.7. HRMS (ESI-MS) *m/z*: [M + H]^+^ calcd for C_24_H_31_N_2_O_5_: 427.2227 (100%); found: 427.2230 (100%).

**FFOMe.TFA**. *d*_H_ (400 MHz, DMSO-d_6_) 9.01 (1H, d, *J* 8, NH), 8.15 (3H, bs, NH_3_), 7.36−7.19 (10H, m, H_Ar_), 4.57 (1H, td, *J* 8, 6, CH), 4.05 (1H, dd, *J* 8, 5, CH), 3.61 (3H, s, CH_3_), 3.09 (2H, td, *J* 14, 6, 1, CH_2_), 2.95 (2H, ddd, *J* 16, 14, 8, CH_2_). *d*_C_ (100 MHz, DMSO-d_6_) 171.1, 168.2, 136.7, 134.7, 129.5, 129.1, 128.5, 128.4, 127.2, 126.7, 53.8, 53.1, 52.0, 36.9, 36.6. HRMS (ESI-MS) *m/z*: [M]^+^ calcd for C_19_H_23_N_2_O_3_: 427.2227 (100%); found: 427.2230 (100%).

**1Br2NapFFOMe**. *d*_H_ (400 MHz, DMSO-d_6_) 8.68 (1H, d, *J* 8, NH), 8.08 (1H, dd, *J* 9, 1, H_Nap_), 8.01 (1H, d, *J* 9, NH), 7.95 (1H, d, *J* 8, H_Nap_), 7.89 (1H, d, *J* 9, H_Nap_), 7.64 (1H, ddd, *J* 8, 7, 1, H_Nap_), 7.48 (1H, ddd, *J* 8, 7, 1, H_Nap_), 7.30−7.14 (11H, m, H_Nap_ and H_Ar_), 4.74−4.65 (3H, m, O-CH_2_ and CH), 4.54 (1H, ddd, *J* 9, 8, 6, CH), 3.61 (3H, s, O-CH_3_), 3.05 (2H, ddd, *J* 14, 10, 5, CH_2_), 2.96 (1H, dd, *J* 14, 9, CH_2_), 2.82 (1H, dd, *J* 14, 9, CH_2_). *d*_C_ (100 MHz, DMSO-d_6_) 171.7, 170.7, 166.8, 152.2, 137.1, 137.0, 132.1, 129.6, 129.3, 129.2, 129.0, 128.3, 128.24, 128.17, 128.0, 126.6, 126.4, 125.2, 124.7, 115.2, 107.6, 67.8, 53.6, 52.9, 51.9, 37.8, 36.6. HRMS (ESI-MS) *m/z*: [M + H]^+^ calcd for C_31_H_30_BrN_2_O_5_: 589.1333 (100%); found: 589.1337 (100%).

**Compound 1**. *d*_H_ (400 MHz, DMSO-d_6_) 12.89 (1H, bs, COOH), 8.51 (1H, d, *J* 8, NH), 8.08 (1H, d, *J* 8.5, H_Nap_), 8.00 (1H, d, *J* 9, NH), 7.94 (1H, d, *J* 8, H_Nap_), 7.88 (1H, d, *J* 9, H_Nap_), 7.64 (1H, t, *J* 8, H_Nap_), 7.47 (1H, t, *J* 7.5, H_Nap_), 7.30−7.12 (11H, m, H_Nap_ and H_Ar_), 4.74−4.63 (3H, m, O-CH_2_, and CH), 4.52−4.45 (1H, m, CH), 3.08 (2H, ddd, *J* 18, 13, 5, CH_2_), 2.94 (1H, dd, *J* 13, 9, CH_2_), 2.82 (1H, dd, *J* 14, 9, CH_2_). *d*_C_ (100 MHz, DMSO-d_6_) 172.7, 170.6, 166.8, 152.2, 137.4, 137.2, 132.1, 129.6, 129.3, 129.2, 129.1, 128.3, 128.2 (2C), 128.0, 126.4, 126.3, 125.3, 124.7, 115.2, 107.6, 67.9, 53.5, 52.9, 37.8, 36.7. HRMS (ESI-MS) *m/z*: [M + Na]^+^ calcd for C_30_H_27_BrN_2_NaO_5_: 597.0996 (100%); found: 597.0998 (100%).

Compound **5** was prepared by the coupling of BocF with WOMe.HCl using isobutylchloroformate in chloroform as per our established procedures to give BocFWOMe^2^. Deprotection with LiOH in THF/water following our established methods gave compound **5**.^2^

**BocFWOMe**. *d*_H_ (400 MHz, DMSO-d_6_) 10.89 (1H, s, NH_indole_), 8.39−8.25 (1H, m, NH), 7.50 (1H, d, *J* 8, H_Ar_), 7.34 (1H, d, *J* 8, H_Ar_), 7.31−7.13 (6H, m, H_indole_ and H_Ar_), 7.07 (1H, t, *J* 8, H_Ar_), 7.00 (1H, t, *J* 7, H_Ar_), 6.87 (0.8H, d, *J* 9, NH), 6.42 (0.2H, d, *J* 10, NH), 4.63−4.51 (1H, m, CH), 4.27−4.06 (1H, m, CH), 3.56 (3H, s, O-CH_3_), 3.14 (2H, qd, *J* 15, 7, CH_2_), 2.97−2.85 (1H, m, CH_2_), 2.75−2.59 (1H, m, CH_2_), 1.29 (7.5H, s, CH_3_), 1.14 (1.5H, s, CH_3_). *d*_C_ (100 MHz, DMSO-d_6_) 172.2, 171.8, 155.1, 138.0, 136.1, 129.2, 127.9, 127.1, 126.1, 123.7, 121.0, 118.4, 118.0, 111.4, 109.1, 78.0, 55.5, 53.0, 51.8, 37.4, 28.1, 27.1. HRMS (ESI-MS) m/z: [M + Na]^+^ calcd for C_26_H_31_N_3_NaO_5_: 488.2156 (100%); found: 488.2176 (100%).

**Compound 5**. *d*_H_ (400 MHz, DMSO-d_6_) 12.61 (1H, bs, COOH), 10.87 (1H, s, NH_indole_), 8.17 (0.2H, d, *J* 7, NH), 8.09 (0.8H, d, *J* 8, NH), 7.55 (1H, d, *J* 8, H_Ar_), 7.34 (1H, d, *J* 8, H_Ar_), 7.28−7.13 (6H, m, H_ArF_ and CH_indole_), 7.07 (1H, t, *J* 8, H_Ar_), 6.99 (1H, t, *J* 7, H_Ar_), 6.88 (0.8H, d, *J* 9, NH), 6.41 (0.2H, d, *J* 8, NH), 4.58−4.47 (1H, m, CH), 4.23−4.05 (1H, m, CH), 3.20 (1H, dd, *J* 15, 5, CH_2_), 3.09 (1H, dd, *J* 15, 8, CH_2_), 2.95 (1H, dd, *J* 14, 4, CH_2_), 2.69 (1H, dd, *J* 14, 10, CH_2_), 1.28 (7.5H, s, CH_3_), 1.12 (1.5H, s, CH_3_). *d*_C_ (100 MHz, DMSO-d_6_) 173.2, 171.6, 155.1, 138.1, 136.1, 129.2, 128.0, 127.3, 126.1, 123.7, 120.9, 118.4, 118.2, 111.3, 109.5, 78.0, 55.7, 52.9, 37.4, 28.1, 27.1. HRMS (ESI-MS) *m/z*: [M + Na]^+^ calcd for C_25_H_29_N_3_NaO_5_: 474.1999 (100%); found: 474.2008 (100%).

Compound **7** was synthesised as follows. BocFLOMe was prepared by the coupling of BocF with LOMe.HCl using our established protocols^2^. This was deprotected with TFA in chloroform to give FLOMe.TFA. FLOMe.TFA was then coupled with 1-naphthoxyacetic acid, followed by deprotection with LiOH in THF/water following our established protocols^2^.

**BocFLOMe**. *d*_H_ (400 MHz, DMSO-d_6_) 8.33 (0.2H, bd, *J* 7.5, NH), 8.24 (0.8H, d, *J* 8, NH), 7.34−7.16 (5H, m, H_Ar_), 6.88 (0.8H, d, *J* 9, NH), 6.46 (0.2H, bd, *J* 7, NH), 4.33 (1H, ddd, *J* 10, 8, 5, CH), 4.23−4.05 (1H, m, CH), 3.61 (3H, s, O-CH_3_), 2.94 (1H, dd, *J* 14, 4, CH_2_), 2.77−2.61 (1H, m, CH_2_), 1.71−1.44 (3H, m, CH_2_-CH-CH_3_), 1.29 (7.5H, s, CH_3_), 1.23 (1.5H, s, CH_3_), 0.90 (3H, d, *J* 7, CH_3_), 0.84 (3H, d, *J* 6, CH_3_). *d*_C_ (100 MHz, DMSO-d_6_) 172.8, 171.9, 155.2, 138.1, 129.2, 128.0, 126.1, 78.0, 55.4, 51.8, 50.1, 39.5, 37.2, 28.1 (0.8C), 27.8 (0.2C), 24.0, 22.8, 21.2. HRMS (ESI-MS) m/z: [M + Na]^+^ calcd for C_21_H_32_N_2_NaO_5_: 415.2203 (100%); found: 415.2211 (100%).

**FLOMe.TFA**. *d*_H_ (400 MHz, DMSO-d_6_) 8.84 (1H, d, *J* 8, NH), 8.16 (3H, bs, NH_3_), 7.37−7.25 (5H, m, H_Ar_), 4.35 (1H, ddd, *J* 9, 8, 6, CH), 4.05 (1H, dd, *J* 8, 6, CH), 3.63 (3H, s, O-CH_3_), 3.12 (1H, dd, *J* 14, 6, CH_2_), 2.94 (1H, dd, *J* 14, 8, CH_2_), 1.70−1.49 (3H, m, CH_2_-CH-CH_3_), 0.91 (3H, d, *J* 6.5, CH_3_), 0.87 (3H, d, *J* 6, CH_3_). *d*_C_ (100 MHz, DMSO-d_6_) 172.1, 168.2, 134.7, 129.5, 128.5, 127.2, 53.2, 52.0, 50.4, 39.5, 36.8, 24.0, 22.7, 21.3. HRMS (ESI-MS) *m/z*: [M + H]^+^ calcd for C_16_H_25_N_2_O_3_: 293.1860 (100%); found: 293.1866 (100%).

**1NapFLOMe**. *d*_H_ (400 MHz, DMSO-d_6_) 8.52 (1H, d, *J* 8, NH or H_Ar_), 8.19−8.12 (2H, m, NH or H_Ar_), 7.89−7.85 (1H, m, H_Ar_), 7.57−7.47 (3H, m, NH or H_Ar_), 7.33 (1H, t, *J* 8, H_Ar_), 7.29−7.18 (5H, m, NH or H_Ar_), 6.72 (1H, *J* 8, NH or H_Ar_), 4.75−4.68 (1H, m, CH), 4.64 (2H, d, *J* 5, O-CH_2_), 4.37−4.30 (1H, m, CH), 3.64 (3H, s, CH_3_), 3.08 (1H, dd, *J* 14, 4, CH), 2.92 (1H, dd, *J* 14, 10, CH), 1.69−1.49 (3H, m, CH_2_-CH), 0.90 (3H, d, *J* 6, CH_3_), 0.85 (3H, d, *J* 6, CH_3_). *d*_C_ (100 MHz, DMSO-d_6_) 172.6, 170.7, 167.0, 153.0, 137.2, 133.9, 129.1, 127.9, 127.3, 126.4, 126.2, 125.8, 125.2, 124.7, 121.5, 120.5, 105.6, 67.1, 52.9, 51.7, 50.3, 37.4, 24.1, 22.6, 21.2. HRMS (ESI-MS) *m/z*: [M + Na]^+^ calcd for C_28_H_32_N_2_NaO_5_: 499.2203 (100%); found: 499.2203 (100%).

**Compound 7**. *d*_H_ (400 MHz, DMSO-d_6_) 12.64 (1H, bs, COOH), 8.40 (1H, d, *J* 8, NH), 8.16−8.09 (2H, m, H_Nap_), 7.87 (1H, dd, *J* 7, 2, H_Nap_), 7.57−7.46 (3H, m, H_Nap_ and/or H_Ar_), 7.33 (1H, t, *J* 8, H_Nap_), 7.29−7.18 (5H, m, H_Nap_ and/or H_Ar_), 6.72 (1H, d, *J* 8, NH), 4.72 (1H, td, *J* 9, 4, CH), 4.68−4.59 (2H, m, O-CH_2_), 4.31−4.23 (1H, m, CH), 3.10 (1H, dd, *J* 14, 4, CH_2_), 2.92 (1H, dd, *J* 14, 9, CH_2_), 1.71−1.49 (3H, m, CH_2_-CH-CH_3_), 0.90 (3H, d, J 6, CH_3_), 0.85 (3H, d, J 6, CH_3_). *d*_C_ (100 MHz, DMSO-d_6_) 173.9, 170.8, 167.1, 153.1, 137.4, 133.9, 129.3, 128.0, 127.4, 126.5, 126.3, 125.9, 125.3, 124.7, 121.6, 120.6, 105.6, 67.1, 53.0, 50.3, 37.5, 24.3, 22.8, 21.3. HRMS (ESI-MS) *m/z*: [M + H]^+^ calcd for C_27_H_31_N_2_O_5_: 463.2227 (100%); found: 463.2227 (100%).

Compound **8** was synthesised as follows. BocFVOMe was prepared by the coupling of BocF with VOMe.HCl using our established protocols^2^. This was deprotected with TFA in chloroform to give FVOMe.TFA. FVOMe.TFA was then coupled with 1-bromo-2-naphthoxyacetic acid, followed by deprotection with LiOH in THF/water following our established protocols^2^.

**BocFVOMe**. *d*_H_ (400 MHz, DMSO-d_6_) 8.18 (0.2H, bd, *J* 8, NH), 8.09 (0.8H, d, *J* 8, NH), 7.37−7.15 (5H, m, H_Ar_), 6.93 (0.9H, d, *J* 9, NH), 6.47 (0.1H, bd, *J* 9, NH), 4.31−4.18 (2H, m, CH), 3.63 (3H, s, O-CH_3_), 2.94 (1H, dd, *J* 14, 4, CH_2_), 2.73 (1H, dd, *J* 14, 10.5, CH_2_), 2.05 (1H, oct., *J* 7, CH-CH_3_), 1.29 (7.5H, s, CH_3_), 1.24 (1.5H, s, CH_3_), 0.89 (6H, dd, *J* 9, 7, CH_3_). *d*_C_ (100 MHz, DMSO-d_6_) 172.1, 171.9, 155.2, 138.1, 129.2, 128.0, 126.2, 78.0, 57.2, 55.4, 51.7, 37.2, 30.1, 28.1, 18.9, 18.1. HRMS (ESI-MS) *m/z*: [M + Na]^+^ calcd for C_20_H_30_N_2_NaO_5_: 401.2047 (100%); found: 401.2058 (100%).

**FVOMe.TFA**. *d*_H_ (400 MHz, DMSO-d_6_) 8.70 (1H, d, *J* 8, NH), 8.17 (3H, bs, NH_3_), 7.36−7.23 (5H, m, H_Ar_), 4.22 (1H, dd, *J* 8, 6, CH), 4.13 (1H, dd, *J* 8, 6, CH), 3.63 (3H, s, O-CH_3_), 3.08 (1H, dd, *J* 14, 6, CH_2_), 2.95 (1H, dd, *J* 14, 8, CH_2_), 2.04 (1H, oct., *J* 7, CH-CH_3_), 0.90 (6H, t, *J* 7, CH_3_). *d*_C_ (100 MHz, DMSO-d_6_) 171.1, 168.3, 134.7, 129.5, 128.5, 127.1, 57.6, 53.1, 51.9, 37.0, 30.1, 18.8, 18.2. HRMS (ESI-MS) *m/z*: [M + H]^+^ calcd for C_15_H_23_N_2_O_3_: 279.1703; found: 279.1713.

**1Br2NapFVOMe**. *d*_H_ (400 MHz, CDCl_3_) 8.46 (1H, d, *J* 8, NH), 8.08 (1H, dd, *J* 8.5, 1, H_Nap_), 8.06 (1H, d, *J* 9, NH), 7.94 (1H, d, *J* 8, H_Nap_), 7.90 (1H, d, *J* 9, H_Nap_), 7.64 (1H, ddd, *J* 8, 7, 1, H_Nap_), 7.47 (1H, ddd, *J* 8, 7, 1, H_Nap_), 7.26−7.17 (6H, m, H_Nap_ and H_Ar_), 4.82 (1H, td, *J* 9, 4.5, CH), 4.76−4.68 (2H, m, O-CH_2_), 4.22 (1H, dd, *J* 8, 6, CH), 3.65 (3H, s, O-CH_3_), 3.07 (1H, dd, *J* 14, 4.5, CH_2_), 2.87 (1H, dd, *J* 14, 9, CH_2_), 2.06 (1H, oct., *J* 7, CH-CH_3_), 0.90 (3H, d, *J* 7, CH_3_), 0.88 (3H, d, *J* 7, CH_3_). *d*_C_ (100 MHz, DMSO-d_6_) 171.8, 171.0, 166.9, 152.2, 137.1, 132.1, 129.6, 129.3, 129.2, 128.3, 128.2, 128.0, 126.4, 125.3, 124.7, 115.2, 107.6, 67.9, 57.5, 52.9, 51.7, 37.9, 29.9, 18.9, 18.3. HRMS (ESI-MS) *m/z*: [M + Na]^+^ calcd for C_27_H_29_N_2_NaO_5_Br: 563.1152 (100%); found: 563.1139 (100%).

**Compound 8**. *d*_H_ (400 MHz, DMSO-d_6_) 12.71 (1H, bs, COOH), 8.31 (1H, d, *J* 8.5, NH), 8.08 (1H, d, *J* 8.5, NH or H_Nap_), 8.04 (1H, d, *J* 8.5, NH or H_Nap_), 7.94 (1H, d, *J* 8, H_Nap_), 7.89 (1H, d, *J* 9, H_Nap_), 7.64 (1H, ddd, *J* 8, 7, 1, H_Nap_), 7.48 (1H, ddd, *J* 8, 7, 1, H_Nap_), 7.26−7.16 (6H, m, H_Nap_ and H_ArF_), 4.82 (1H, td, *J* 9, 4, CH), 4.75−4.67 (2H, m, O-CH_2_), 4.19 (1H, dd, *J* 8.5, 6, CH), 3.08 (1H, dd, *J* 14, 4, CH_2_), 2.87 (1H, dd, *J* 14, 9, CH_2_), 2.08 (1H, oct., *J* 7, CH-CH_3_), 0.91 (3H, d, *J* 4, CH_3_), 0.89 (3H, d, *J* 4, CH_3_). *d*_C_ (100 MHz, DMSO-d_6_) 172.8, 170.9, 166.9, 152.2, 137.2, 132.1, 129.6, 129.3, 129.2, 128.3, 128.2, 128.0, 126.3, 125.3, 124.7, 115.2, 107.6, 67.9, 57.2, 52.9, 37.9, 29.9, 19.1, 18.0. HRMS (ESI-MS) *m/z*: [M + Na]^+^ calcd for C_26_H_27_N_2_NaO_5_Br: 549.0996 (100%); found: 549.0975 (100%).

Compound **11** was synthesised as follows. BocFIOMe was prepared by the coupling of BocF with IOMe.HCl using our established protocols^2^. This was deprotected with TFA in chloroform to give FIOMe.TFA. FIOMe.TFA was then coupled with 6-methoxy-2-naphthoxyacetic acid, followed by deprotection with LiOH in THF/water following our established protocols^2^.

**BocFIOMe**. *d*_H_ (400 MHz, DMSO-d_6_) 8.20 (0.2H, bd, *J* 8, NH), 8.09 (0.8H, bd, *J* 8, NH), 7.42−7.13 (5H, m, H_Ar_), 6.93 (0.9H, d, *J* 9, NH), 6.46 (0.1H, bd, *J* 8.5, NH), 4.30−4.21 (2H, m, CH), 3.62 (3H, s, O-CH_3_), 2.94 (1H, dd, *J* 14, 4, CH_2_), 2.73 (1H, dd, *J* 14, 10, CH_2_), 1.86−1.74 (1H, m, CH-CH_3_), 1.47−1.38 (1H, m, CH_2_-CH_3_), 1.32−1.12 (10H, m, CH_2_-CH_3_ and CH_3_), 0.90−0.80 (6H, m, CH_3_). *d*_C_ (100 MHz, DMSO-d_6_) 172.0, 171.9, 155.2, 138.1, 129.2, 128.0, 126.1, 78.0, 56.2, 55.4, 51.6, 37.2, 36.4, 28.1, 24.6, 15.3, 11.1. HRMS (ESI-MS) *m/z*: [M + Na]^+^ calcd for C_21_H_32_N_2_NaO_5_: 415.2203 (100%); found: 415.2214 (100%).

**FIOMe.TFA**. *d*_H_ (400 MHz, DMSO-d_6_) 8.74 (1H, d, *J* 8, NH), 8.24 (3H, bs, NH_3_), 7.35−7.23 (5H, m, H_Ar_), 4.25 (1H, dd, *J* 8, 6.5, CH), 4.14 (1H, t, *J* 7, CH), 3.62 (3H, s, O-CH_3_), 3.07 (1H, dd, *J* 14, 6, CH_2_), 2.96 (1H, dd, *J* 14, 7, CH_2_), 1.84−1.71 (1H, m, CH-CH_3_), 1.49−1.37 (1H, m, CH_2_), 1.24−1.12 (1H, m, CH_2_), 0.88−0.82 (6H, m, CH_3_). *d*_C_ (100 MHz, DMSO-d_6_) 171.6, 168.7, 135.2, 130.0, 128.9, 127.6, 57.0, 53.5, 52.3, 37.4, 36.9, 25.2, 15.8, 11.6. HRMS (ESI-MS) *m/z*: [M + H]^+^ calcd for C_16_H_25_N_2_O_3_: 293.1860 (100%); found: 293.1880 (100%).

**6OMe2NapFIOMe**. *d*_H_ (400 MHz, DMSO-d_6_) 8.44 (1H, d, *J* 8, NH), 8.18 (1H, d, *J* 8, NH or H_Ar_), 7.73 (1H, d, *J* 10, NH or H_Ar_), 7.64 (1H, d, *J* 9, NH or H_Ar_), 7.27 (1H, d, *J* 1, H_Ar_), 7.25−7.11 (8H, m, H_Ar_), 4.75 (1H, td, *J* 9, 4, CH), 4.51 (2H, s, O-CH_2_), 4.24 (1H, dd, *J* 8, 6, CH), 3.84 (3H, s, O-CH_3_), 3.64 (3H, s, O-CH_3_), 3.02 (1H, dd, *J* 14, 4, CH_2_), 2.88 (1H, dd, *J* 14, 9.5, CH_2_), 1.83−1.73 (1H, m, CH-CH_3_), 1.44−1.34 (1H, m, CH_2_-CH_3_), 1.23−1.10 (1H, m, CH_2_-CH_3_), 0.82 (6H, t, *J* 8, CH_3_).

**Compound 11**. *d*_H_ (400 MHz, DMSO-d_6_) 12.91 (1H, bs, COOH), 8.27 (1H, d, *J* 8, NH), 8.18 (1H, d, *J* 8.5, NH or H_Ar_), 7.73 (1H, d, *J* 10, NH or H_Ar_), 7.64 (1H, d, *J* 9, NH or H_Ar_), 7.27 (1H, d, *J* 3, H_Ar_), 7.24 (2H, dd, *J* 8, 2, H_Ar_), 7.21−7.11 (6H, m, H_Ar_), 4.74 (1H, td, *J* 9, 4, CH), 4.52 (2H, s, O-CH_2_), 4.20 (1H, dd, *J* 8, 6, CH), 3.84 (3H, s, O-CH_3_), 3.04 (1H, dd, *J* 14, 4, CH_2_), 2.89 (1H, dd, *J* 14, 10, CH_2_), 1.84−1.73 (1H, m, CH-CH_3_), 1.47−1.36 (1H, m, CH_2_-CH_3_), 1.23−1.11 (1H, m, CH_2_-CH_3_), 0.87−0.79 (6H, m, CH_3_). *d*_C_ (100 MHz, DMSO-d_6_) 172.9, 170.9, 167.5, 155.9, 153.9, 137.6, 129.7, 129.3, 129.2, 128.2, 127.9, 126.3, 118.8, 118.7, 107.7, 106.1, 66.8, 56.6, 55.1, 53.2, 37.5, 36.5, 24.7, 15.6, 11.4. HRMS (ESI-MS) *m/z*: [M + Na]^+^ calcd for C_28_H_32_N_2_NaO_6_: 515.2153; found: 515.2177.

Compound **12** was synthesised as follows. First, 6Br2NapA was prepared as described elsewhere^2^. BocVLOMe was prepared by coupling BocV with LOMe.HCl using our standard methods^2^. This was deprotected with TFA to give VLOMe.TFA using our established protocols^2^. 6Br2NapA was coupled with VLOMe.TFA to give 6Br2NapAVLOMe, followed by deprotection with LiOH in THF/water following our established protocols^2^.

**BocVLOMe**. *d*_H_ (400 MHz, DMSO-d_6_) 8.14 (1H, d, *J* 8, NH), 6.64 (0.8H, d, *J* 9, NH), 6.24 (0.2H, bs, NH), 4.34−4.26 (1H, m, CH), 3.80−3.73 (1H, m, CH), 3.61 (0.3H, s, O-CH_3_), 3.60 (2.7H, s, O-CH_3_), 1.95−1.84 (1H, m, CH-CH_3_), 1.72−1.43 (3H, m, CH_2_-CH-CH_3_), 1.37 (9H, s, CH_3_), 0.91−0.79 (12H, m, CH_3_). *d*_C_ (100 MHz, DMSO-d_6_) 172.8, 171.6, 155.3, 77.9, 59.6, 51.7, 50.0, 39.6, 30.3, 28.1, 24.0, 22.8, 21.1, 19.1, 18.2. HRMS (ESI-MS) *m/z*: [M + Na]^+^ calcd for C_17_H_32_N_2_NaO_5_: 367.2203 (100%); found: 367.2217 (100%).

**VLOMe.TFA**. *d*_H_ (400 MHz, DMSO-d_6_) 8.76 (1H, d, *J* 7, NH), 8.15 (3H, bs, NH_3_), 4.33 (1H, ddd, *J* 10, 7, 5, CH), 3.65 (1H, d, *J* 6, CH), 3.63 (3H, s, O-CH_3_), 2.11 (1H, oct., *J* 6.5, CH-CH_3_), 1.74−1.46 (3H, m, CH_2_-CH-CH_3_), 0.95 (6H, t, *J* 6.5, CH_3_), 0.91 (3H, d, *J* 6.5, CH_3_), 0.86 (3H, d, *J* 6.5, CH_3_). *d*_C_ (100 MHz, DMSO-d_6_) 172.4, 168.1, 158.0 (1C, d, ^2^*J*_C-F_ 31), 117.3 (1C, d, ^1^*J*_C-F_ 300), 57.1, 51.9, 50.5, 39.5, 29.9, 24.1, 22.7, 21.2, 18.1, 17.4. HRMS (ESI-MS) *m/z*: [M + H]^+^ calcd for C_12_H_25_N_2_O_3_: 245.1860; found: 245.1867.

**6Br2NapAVLOMe**. *d*_H_ (400 MHz, DMSO-d_6_) 8.30 (1H, d, *J* 7, NH), 8.22 (1H, d, *J* 8, NH), 8.12 (1H, d, *J* 2, H_Nap_), 7.90 (1H, d, *J* 9, NH), 7.85 (1H, d, *J* 9, H_Nap_), 7.77 (1H, d, *J* 9, H_Nap_), 7.57 (1H, dd, *J* 9, 2, H_Nap_), 7.33 (1H, d, *J* 3, H_Nap_), 7.30 (1H, dd, *J* 9, 3, H_Nap_), 4.69−4.61 (2H, m, O-CH_2_), 4.47 (2H, quin., *J* 7, CH), 4.27 (1H, ddd, *J* 10, 7, 5, CH), 4.18 (1H, dd, *J* 9, 7, CH), 3.60 (3H, s, O-CH_3_), 1.94 (1H, oct., *J* 7, CH-CH_3_), 1.67−1.44 (3H, m, CH_2_-CH-CH_3_), 1.24 (3H, d, *J* 7, CH_3_), 0.88 (3H, d, *J* 6, CH_3_), 0.86−0.78 (9H, m, CH_3_). *d*_C_ (100 MHz, DMSO-d_6_) 172.7, 171.8, 171.0, 166.9, 156.0, 132.7, 129.9, 129.4, 129.3, 129.0, 128.7, 119.7, 116.6, 107.5, 66.8, 57.3, 51.7, 50.2, 47.8, 39.3, 30.7, 24.2, 22.7, 21.2, 19.0, 18.3, 18.0. HRMS (ESI-MS) *m/z*: [M + Na]^+^ calcd for C_27_H_36_BrN_3_NaO_6_: 600.1680 (100%); found: 600.1687 (97%).

**Compound 12**. *d*_H_ (400 MHz, DMSO-d_6_) 12.52 (1H, bs, COOH), 8.25 (1H, d, *J* 8, NH), 8.18 (1H, d, *J* 8, NH), 8.12 (1H, d, *J* 2, H_Nap_), 7.93 (1H, d, *J* 9, NH or H_Nap_), 7.85 (1H, d, *J* 9, NH or H_Nap_), 7.77 (1H, d, *J* 9, NH or H_Nap_), 7.57 (1H, dd, *J* 9, 2, H_Nap_), 7.35−7.27 (2H, m, NH or H_Nap_), 4.69−4.61 (2H, m, O-CH_2_), 4.47 (1H, quin., *J* 7, CH), 4.24−4.15 (2H, m, CH_V_), 1.94 (1H, sex., *J* 7, CH-CH_3_), 1.68−1.43 (3H, m, CH_2_-CH-CH_3_), 1.23 (3H, d, *J* 7, CH_3_), 0.88 (3H, d, *J* 6.5, CH_3_), 0.85−0.76 (9H, m, CH_3_). *d*_C_ (100 MHz, DMSO-d_6_) 174.1, 172.1, 171.2, 167.4, 156.2, 132.9, 130.1, 129.5, 129.3, 128.9, 119.9, 116.8, 107.7, 66.9, 57.7, 50.4, 48.2, 30.9, 24.5, 23.0, 21.4, 19.3, 18.4, 18.2. HRMS (ESI-MS) *m/z*: [M + Na]^+^ calcd for C_26_H_34_BrN_3_NaO_6_: 586.1523 (100%); found 586.1531 (100%).

Compound **13** was synthesised as follows. First, 1Br2NapL was prepared as described elsewhere.^11^ FFOMe.TFA was prepared as described previously^11,12^. 1Br2NapL was coupled with FFOMe.TFA to give 1Br2NapLFFOMe, followed by deprotection using LiOH in THF/water following our established protocols^2^ to give compound **13**.

**1Br2NapLFFOMe**. *d*_H_ (400 MHz, DMSO-d_6_) 8.45 (1H, d, *J* 7, NH), 8.17 (1H, d, *J* 8, NH), 8.10 (1H, d, *J* 8.5, H_Nap_), 7.99−7.90 (3H, m, NH and H_Nap_), 7.65 (1H, ddd, *J* 8, 7, 1, H_Nap_), 7.39 (1H, d, J 9, H_Nap_), 7.29−7.12 (10H, m, H_Ar_), 4.82−4.72 (2H, m, O-CH_2_), 4.58 (1H, td, *J* 9, 5, CH), 4.47 (1H, q, *J* 7, CH), 4.37 (1H, td, J 9, 6, CH), 3.56 (3H, s, O-CH_3_), 3.06−2.90 (3H, m, CH_2_), 2.76 (1H, dd, *J* 13, 9.5, CH_2_), 1.54−1.43 (1H, m, CH-CH_3_), 1.40−1.30 (2H, m, CH_2_-CH-CH_3_), 0.81 (6H, dd, *J* 6.5, 3.5, CH-CH_3_). *d*_C_ (100 MHz, DMSO-d_6_) 171.6, 171.2, 171.0, 166.8, 152.3, 137.5, 136.9, 132.1, 129.7, 129.2, 129.1, 129.0, 128.3, 128.22, 128.19, 127.9, 126.5, 126.2, 125.3, 124.7, 115.3, 107.6, 68.0, 53.6, 53.3, 51.8, 50.6, 41.3, 37.4, 36.5, 24.1, 23.0, 21.6. HRMS (ESI-MS) *m/z*: [M + Na]^+^ calcd for C_37_H_40_BrN_3_NaO_6_: 724.1993 (100%); found 724.2004 (100%).

**Compound 13**. *d*_H_ (400 MHz, DMSO-d_6_) 12.76 (1H, bs, COOH), 8.25 (1H, d, *J* 8, NH), 8.16 (1H, d, *J* 8.5, NH), 8.10 (1H, d, *J* 8.5, H_Nap_), 7.98−7.90 (3H, m, NH and H_Nap_), 7.65 (1H, ddd, *J* 8, 7, 1, H_Nap_), 7.48 (1H, ddd, *J* 8, 7, 1, H_Nap_), 7.38 (1H, d, *J* 9, H_Nap_), 7.28−7.17 (9H, m, H_Ar_), 7.16−7.11 (1H, m, H_Ar_), 4.81−4.72 (2H, m, O-CH_2_), 4.57 (1H, td, *J* 10, 4.5, CH), 4.44 (1H, td, *J* 8, 5, CH), 4.37 (1H, td, *J* 8, 6, CH), 3.06 (1H, dd, *J* 14, 5.5, CH_2_), 3.01 (1H, dd, *J* 14, 4.5, CH_2_), 2.93 (1H, dd, *J* 14, 8, CH_2_), 2.76 (1H, dd, *J* 14, 10, CH_2F_), 1.53−1.43 (1H, m, CH-CH_3_), 1.40−1.32 (2H, m, CH_2_-CH-CH_3_), 0.81 (3H, d, *J* 3, CH_3_), 0.79 (3H, d, *J* 3, CH_3_). *d*_C_ (100 MHz, DMSO-d_6_) 172.6, 171.2, 170.9, 166.8, 152.3, 137.6, 137.3, 132.2, 129.7, 129.23, 129.16, 129.08, 128.3, 128.18, 128.16, 127.9, 126.4, 126.2, 125.3, 124.7, 115.3, 107.6, 68.0, 53.5, 53.4, 50.6, 41.3, 37.4, 36.6, 24.1, 23.0, 21.6.

Compound **16** was prepared as follows. 4-Chloro-1-naphthoxy acetic acid, prepared as described elsewhere^11^, was coupled with IFOEt.TFA (prepared as described elsewhere^11^) followed by deprotection with LiOH in THF/water following our established methods^2^.

**4Cl1NapIFOEt**. *d*_H_ (400 MHz, DMSO-d_6_) 8.56 (1H, d, *J* 7, NH), 8.29 (1H, ddd, *J* 8, 1, 1, H_Nap_), 8.13 (1H, ddd, *J* 8.5, 1, 1, H_Nap_), 7.99 (1H, d, *J* 9, NH), 7.74 (1H, ddd, *J* 8, 7, 1, H_Nap_), 7.67 (1H, ddd, *J* 8, 7, 1, H_Nap_), 7.57 (1H, d, *J* 8, H_Nap_), 7.27−7.15 (5H, m, H_Ar_), 6.88 (1H, d, *J* 8, H_Nap_), 4.82−4.73 (2H, m, O-CH_2_-C=O), 4.47 (1H, ddd, *J* 9, 7, 6, CH), 4.33 (1H, dd, *J* 9, 7, CH), 4.01 (2H, q, *J* 7, O-CH_2_-CH_3_), 3.02 (1H, dd, *J* 14, 6, CH_2_), 2.94 (1H, dd, *J* 14, 9, CH_2_), 1.79−1.68 (1H, m, CH-CH_3_), 1.43−1.33 (1H, m, CH-CH_2_-CH_3_), 1.08 (3H, t, *J* 7, O-CH_2_-CH_3_), 1.05−0.95 (1H, m, CH-CH_2_-CH_3_), 0.81 (3H, d, *J* 7, CH-CH_3_), 0.78 (3H, t, *J* 7, CH_2_-CH­_3_). *d*_C_ (100 MHz, DMSO-d_6_) 171.1, 170.7, 166.8, 152.6, 137.0, 130.4, 129.0, 128.1 (2C), 126.5 (2C), 126.0, 125.9, 123.6, 122.28, 122.27, 106.1, 67.2, 60.4, 56.1, 53.5, 37.0, 36.5, 24.0, 15.1, 13.9, 10.9. HRMS (ESI-MS) *m/z*: [M + Na]^+^ calcd for C_29_H_33_ClN_2_NaO_5_: 547.1970 (100%); found: 547.1984 (100%).

**Compound 16**. *d*_H_ (400 MHz, DMSO-d_6_) 12.69 (1H, s, COOH), 8.39 (1H, d, *J* 8, NH_F_), 8.29 (1H, d, *J* 8, H_Nap_), 8.13 (1H, d, *J* 8, H_Nap_), 7.95 (1H, d, *J* 9, NH_I_), 7.74 (1H, ddd, *J* 8, 7, 1, H_Nap_), 7.67 (1H, ddd, *J* 8, 7, 1, H_Nap_), 7.57 (1H, d, *J* 8, H_Nap_), 7.26−7.13 (5H, m, H_Ar_), 6.88 (1H, d, *J* 8, H_Nap_), 4.82−4.73 (2H, m, O-CH_2_), 4.46 (1H, ddd, *J* 9, 8, 5, CH_F_), 4.32 (1H, dd, *J* 9, 7, NH_I_-CH_I_), 3.06 (1H, dd, *J* 14, 5, CH_2F_), 2.89 (1H, dd, *J* 14, 9, CH_2F_), 1.79−1.69 (1H, m, CH_I_-CH_2I_), 1.42−1.31 (1H, m, CH_2I_-CH_3I_), 1.05−0.92 (1H, m, CH_2I_-CH_3I_), 0.80 (3H, d, *J* 7, CH_I_-CH_3I_), 0.77 (3H, t, *J* 7, CH_2I_-CH_3I_). *d*_C_ (100 MHz, DMSO-d_6_) 172.7 (C=O_F_), 170.7 (C=O_I_), 166.8 (CH_2_-C=O), 152.6 (C_Nap_-O), 137.5 (C_Ar_), 130.4 (C_Nap_), 129.0 (CH_Ar_), 128.1 (2C, CH_Nap_ and CH_Ar_), 126.5 (CH_Nap_), 126.4 (CH_Ar_), 126.1 (CH_Nap_), 125.9 (C_Nap_), 123.6 (CH_Nap_), 122.29 (CH_Nap_ or C_Nap_), 122.28 (CH_Nap_ or C_Nap_), 106.1 (CH_Nap_), 67.2 (O-CH_2_), 56.2 (NH_I_-CH_I_), 53.3 (CH_F_), 37.1 (CH_I_-CH_2I_), 36.6 (CH_2F_), 24.0 (CH_2I_-CH_3I_), 15.2 (CH_I_-CH_3I_), 10.9 (CH_2I_-CH_3I_). HRMS (ESI-MS) *m/z*: [M + Na]^+^ calcd for C_27_H_29_ClN_2_NaO_5_: 519.1657 (100%); found: 519.1664 (100%).

Compound **18** was synthesised as follows. First, BocAFFOEt was prepared by coupling BocA with FFOEt.TFA (synthesised as described elsewhere^2^) using our established protocols^2^. This was deprotected with TFA in chloroform to give AFFOEt.TFA. AFFOEt.TFA was coupled with 4-methyl-1-naphthoic acid, followed by deprotection with LiOH in THF/water following our established protocols^2^.

**BocAFFOEt**. *d*_H_ (400 MHz, DMSO-d_6_) 8.60−8.53 (0.2H, m, NH), 8.49 (0.8H, d, *J* 7, NH), 7.73 (1H, d, *J* 8, NH), 7.33−7.13 (10H, m, H_Ar_), 6.88 (0.8H, d, *J* 8, NH), 6.50−6.39 (0.1H, m, NH), 4.60−4.50 (1H, m, CH), 4.45 (1H, q, *J* 7, CH), 4.02 (2H, q, *J* 7, CH_2_-CH_3_), 3.94−3.83 (1H, m, CH), 3.06−2.90 (3H, m, CH_2_), 2.76 (1H, dd, *J* 14, 9, CH_2_), 1.35 (7.5H, s, CH_3_), 1.18 (1.5H, s, CH_3_), 1.08 (3H, t, *J* 7, CH_2_-CH_3_), 1.04 (3H, d, *J* 7, CH_3_). *d*_C_ (100 MHz, DMSO-d_6_) 172.2, 171.1, 171.0, 154.9, 137.4, 136.9, 129.3, 129.0, 128.2, 127.9, 126.6, 126.2, 78.1, 60.5, 53.6, 53.1, 49.8, 37.7 36.8, 28.2, 18.2, 13.9. HRMS (ESI-MS) *m/z*: [M + H]^+^ calcd for C_28_H_38_N_3_O_6_: 512.2755; found: 512.2764.

**AFFOEt.TFA**. *d*_H_ (400 MHz, DMSO-d_6_) 8.63 (1H, d, *J* 7, NH), 8.55 (1H, d, *J* 8, NH), 7.96 (3H, bs, NH_3_), 7.34−7.18 (10H, m, H_Ar_), 4.64−4.55 (1H, m, CH), 4.51−4.43 (1H, m, CH), 4.03 (2H, q, *J* 7, CH_2_-CH_3_), 3.74 (1H, q, *J* 7, CH), 3.07−2.91 (3H, m, CH_2_), 2.76 (1H, dd, *J* 14, 10, CH_2_), 1.28 (3H, d, *J* 7, CH_3_), 1.10 (3H, t, *J* 7, CH_2_-CH_3_). *d*_C_ (100 MHz, DMSO-d_6_) 171.2, 170.9, 169.45, 158.3 (1C, q, ^2^*J*_C-F_ 31), 137.5, 137.1, 129.2, 129.1, 128.3, 128.2, 126.6, 126.4, 117.3 (1C, q, ^1^*J*_C-F_ 300), 60.6, 54.1, 53.7, 48.0, 37.5, 36.7, 17.2, 14.0. HRMS (ESI-MS) *m/z*: [M]^+^ calcd for C_23_H_30_N_3_O_4_: 412.2231; found: 412.2241.

**4Me1NapAFFOEt**. *d*_H_ (500 MHz, DMSO-d_6_) 8.54−8.47 (2H, m, NH), 8.23 (1H, d, *J* 8, H_Ar_), 8.06 (1H, d, *J* 8, H_Ar_), 7.97 (1H, d, *J* 8, NH), 7.61−7.56 (1H, m, H_Ar_), 7.55−7.50 (1H, m, H_Ar_), 7.47 (1H, d, *J* 7, H_Ar_), 7.39 (1H, d, *J* 7, H_Ar_), 7.29−7.14 (10H, m, H_Ar_), 4.64 (1H, m, CH), 4.53−4.44 (2H, m, CH), 4.02 (2H, q, *J* 7, CH_2_-CH_3_), 3.06−2.93 (3H, m, CH_2_), 2.83 (1H, dd, *J* 14, 9, CH_2_), 2.68 (3H, s, CH_3_), 1.24 (3H, d, *J* 7, CH_3_), 1.08 (3H, t, *J* 7, CH_2_-CH_3_). *d*_C_ (125 MHz, DMSO-d_6_) 172.0, 171.2, 171.1, 168.5, 137.6, 137.0, 136.3, 132.7, 132.1, 129.9, 129.3, 129.1, 128.3, 128.0, 126.6, 126.3, 126.2, 126.2, 126.1, 125.4, 125.2, 124.3, 60.5, 53.8, 53.4, 49.0, 37.6, 36.8, 19.3, 17.8, 13.9. HRMS (ESI-MS) *m/z*: [M + H]^+^ calcd for C_35_H_38_N_3_O_5_: 580.2806; found: 580.2815.

**Compound 18**. *d*_H_ (400 MHz, DMSO-d_6_) 12.81 (1H, bs, COOH), 8.51 (1H, d, *J* 7, NH), 8.31 (1H, d, *J* 8, NH), 8.24−8.20 (1H, m, H_Ar_), 8.08−8.04 (1H, m, H_Ar_), 7.95 (1H, d, *J* 8, NH), 7.58 (1H, ddd, *J* 8, 7, 1, H_Ar_), 7.52 (1H, ddd, *J* 8, 7, 1, H_Ar_), 7.46 (1H, d, *J* 7, H_Ar_), 7.40−7.36 (1H, m, H_Ar_), 7.29−7.12 (10H, m, H_Ar_), 4.59 (1H, td, *J* 9, 5, CH), 4.53−4.40 (2H, m, CH), 3.07 (1H, dd, *J* 14, 5, CH_2_), 3.04 (1H, dd, *J* 14, 4, CH_2_), 2.93 (1H, dd, *J* 14, 9, CH_2_), 2.82 (1H, dd, *J* 14, 9, CH_2_), 2.68 (3H, s, CH_3_), 1.23 (3H, d, *J* 7, CH_3_). *d*_C_ (100 MHz, DMSO-d_6_) 172.7, 172.0, 170.9, 168.5, 137.6, 137.4, 126.3, 132.7, 132.1, 130.0, 129.3, 129.1, 128.2, 128.0, 126.5, 126.3, 126.2, 126.1, 125.4, 125.2, 124.2, 53.5, 53.5, 49.0, 37.6, 36.8, 19.3, 17.8. HRMS (ESI-MS) *m/z*: [M + H]^+^ calcd for C_33_H_34_N_3_O_5_: 552.2493; found: 552.2503.

Compound **19** was prepared as follows. First, 6-benzoylnaphth-2-ol was reacted with tert-butyl chloroacetate, followed by deprotection with TFA to give 2‐[(6‐benzoylnaphthalen‐2‐yl)oxy]acetic acid following our established procedures.^11^ BocFAOMe was prepared by coupling BocF and AOMe.HCl. After Boc deprotection, FAOMe.TFA was coupled with BocM. Boc deprotection afforded MFAOMe.TFA, which was coupled to 2‐[(6‐benzoylnaphthalen‐2‐yl)oxy]acetic acid, followed by deprotection with LiOH in THF/water following our established protocols^2^.

***tert*-Butyl 2-[(6-benzoylnaphthalen-2-yl)oxy]acetate**. *d*_H_ (400 MHz, DMSO-d_6_) 8.27 (1H, d, *J* 1, H_Ar_), 8.06 (1H, d, *J* 9, H_Ar_), 7.94 (1H, d, *J* 9, H_Ar_), 7.84 (1H, dd, *J* 9, 2, H_Ar_), 7.81−7.77 (2H, m, H_Ar_), 7.72−7.68 (1H, m, H_Ar_), 7.62−7.56 (2H, m, H_Ar_), 7.38 (1H, d, *J* 2, H_Ar_), 7.29 (1H, dd, *J* 9, 3, H_Ar_), 4.84 (2H, s, O-CH_2_), 1.45 (9H, s, CH_3_). *d*_C_ (100 MHz, DMSO-d_6_) 195.6, 167.5, 157.7, 137.6, 136.4, 132.4, 132.3, 131.5, 131.4, 129.6, 128.6, 127.4, 127.2, 126.0, 119.4, 107.3, 81.6, 65.1, 27.7. HRMS (ESI-MS) *m/z*: [M + H]^+^ calcd for C_23_H_23_O_4_: 363.1591; found: 363.1600.

**2-[(6-Benzoylnaphthalen-2-yl)oxy]acetic acid**. *d*_H_ (400 MHz, DMSO-d_6_) 13.11 (1H, bs, COOH), 8.27 (1H, d, *J* 1, H_Ar_), 8.05 (1H, d, *J* 9, H_Ar_), 7.95 (1H, d, *J* 9, H_Ar_), 7.84 (1H, dd, *J* 9, 2, H_Ar_), 7.81−7.77 (2H, m, H_Ar_), 7.72−7.68 (1H, m, H_Ar_), 7.61−7.57 (2H, m H_Ar_), 7.41 (1H, d, *J* 2, H_Ar_), 7.30 (1H, dd, *J* 9, 3, H_Ar_), 4.87 (2H, s, CH_2_). *d*_C_ (100 MHz, DMSO-d_6_) 195.6, 169.8, 157.8, 137.6, 136.4, 132.4, 132.2, 131.5, 131.3, 129.6, 128.5, 127.4, 127.2, 126.0, 119.5, 107.1, 64.6. HRMS (ESI-MS) *m/z*: [M + Na]^+^ calcd for C_19_H_14_NaO_4_: 329.0784; found: 329.0782.

**BocFAOMe**. *d*_H_ (400 MHz, DMSO-d_6_) 8.44−8.40 (0.2H, m, NH), 8.37 (0.8H, d, *J* 7, NH), 7.33−7.24 (4H, m, H_Ar_), 7.23−7.15 (1H, m, H_Ar_), 6.87 (0.8H, d, *J* 9, NH), 6.44 (0.2H, d, *J* 9, NH), 4.35 (1H, m, CH), 4.22−4.14 (0.8H, m, CH), 4.12−4.05 (0.2H, m, CH), 3.62 (3H, s, O-CH_3_), 2.97 (1H, dd, *J* 14, 4, CH_2_), 2.70 (1H, dd, *J* 14, 11, CH_2_), 1.30 (3H, d, *J* 7, CH_3A_), 1.28 (7.5H, s, CH_3_), 1.22 (1.5H, s, CH_3_). *d*_C_ (100 MHz, DMSO-d_6_) 173., 171.8, 155.2, 138.2, 129.2, 128.0, 126.1, 77.9, 55.4, 51.9, 47.6, 37.4, 28.1, 16.9. HRMS (ESI-MS) *m/z*: [M + Na]^+^ calcd for C_18_H_26_N_2_NaO_5_: 373.1734; found: 373.1735.

**FAOMe.TFA**. *d*_H_ (400 MHz, DMSO-d_6_) 8.93 (1H, d, *J* 7, NH), 8.19 (3H, bs, NH_3_), 7.38−7.21 (5H, m, H_Ar_), 4.41 (1H, m, CH), 4.06−3.98 (1H, m, CH), 3.63 (3H, s, O-CH_3_), 3.12 (1H, dd, *J* 14, 6, CH_2_), 2.94 (1H, dd, *J* 14, 8, CH_2_), 1.31 (3H, d, *J* 7, CH_3_). *d*_C_ (100 MHz, DMSO-d_6_) 172.3, 168.0, 158.4 (1C, q, ^2^*J*_C-F_ 31), 134.9, 129.6, 128.5, 127.2, 117.2 (1C, q, ^1^*J*_C-F_ 300), 53.3, 52.1, 47.8, 36.9, 16.9. HRMS (ESI-MS) *m/z*: [M + Na]^+^ calcd for C_13_H_18_N_2_NaO_3_: 273.1210; found: 273.1212.

**BocMFAOMe**. *d*_H_ (400 MHz, DMSO-d_6_) 8.59−8.53 (0.2H, m, NH), 8.48 (0.8H, d, *J* 7, NH), 7.79 (1H, d, *J* 8, NH), 7.29−7.14 (5H, m, H_Ar_), 6.96 (0.8H, d, *J* 8, NH), 6.57−6.47 (0.2H, m, NH), 4.62−4.52 (1H, m, CH), 4.30−4.23 (1H, m, CH), 3.96−3.87 (0.8H, m, CH), 3.86−3.78 (0.2H, m, CH), 3.62 (3H, s, O-CH_3_), 3.01 (1H, dd, *J* 14, 4, CH_2_), 2.78 (1H, dd, *J* 14, 9, CH_2_), 2.37−2.24 (2H, m, S-CH_2_), 1.99 (3H, s, S-CH_3_), 1.74−1.60 (2H, m, S-CH_2_-CH_2_), 1.36 (7.5H, s, CH_3_), 1.28 (3H, d, *J* 7, CH_3_), 1.22 (1.5H, s, CH_3_). *d*_C_ (100 MHz, DMSO-d_6_) 172.8, 171.3, 170.8, 155.2, 137.4, 129.3, 127.9, 126.2, 78.2, 53.8, 53.1, 51.9, 47.6, 37.7, 31.8, 29.6, 28.1, 16.8, 14.6. HRMS (ESI-MS) *m/z*: [M + Na]^+^ calcd for C_23_H_35_N_3_NaO_6_S: 504.2139; found: 504.2149.

**MFAOMe.TFA**. *d*_H_ (400 MHz, DMSO-d_6_) 8.66 (1H, d, *J* 8, NH), 8.64 (1H, d, *J* 7, NH), 8.09 (3H, bs, NH_3_), 7.35−7.26 (4H, m, H_Ar_), 7.25−7.19 (1H, m, H_Ar_), 4.63 (1H, m, CH), 4.32−4.25 (1H, m, CH), 3.80 (1H, t, *J* 6, CH), 3.62 (3H, s, O-CH_3_), 3.07 (1H, dd, *J* 14, 4, CH_2_), 2.80 (1H, dd, *J* 14, 10, CH_2_), 2.50−2.44 (2H, m, S-CH_2_), 2.05 (3H, s, S-CH_3_), 2.01−1.92 (2H, m, S-CH_2_-CH_2_), 1.30 (3H, d, *J* 7, CH_3_). *d*_C_ (100 MHz, DMSO-d_6_) 172.8, 170.8, 168.2, 158.3 (1C, q, ^2^*J*_C-F_ 31), 137.6, 129.2, 128.2, 126.5, 117.2 (1C, q, ^1^*J*_C-F_ 300), 54.2, 51.9, 51.5, 47.69, 37.1, 31.2, 27.9, 16.9, 14.4. HRMS (ESI-MS) *m/z*: [M]^+^ calcd for C_18_H_28_N_3_O_4_S: 382.1795; found: 382.1806.

**6Bn2NapMFAOMe**. *d*_H_ (400 MHz, DMSO-d_6_) 8.44 (1H, d, *J* 7, NH), 8.27 (1H, d, *J* 1, H_Ar_), 8.23 (1H, d, *J* 8, NH), 8.14 (1H, d, *J* 8, NH), 8.06 (1H, d, *J* 9, H_Ar_), 7.94 (1H, d, *J* 9, H_Ar_), 7.84 (1H, dd, *J* 9, 2, H_Ar_), 7.81−7.76 (2H, m, H_Ar_), 7.73−7.67 (1H, m, H_Ar_), 7.62−7.56 (2H, m, H_Ar_), 7.39 (1H, d, *J* 2, H_Ar_), 7.33 (1H, dd, *J* 9, 3, H_Ar_), 7.27−7.12 (5H, m H_Ar_), 4.75 (1H, d, *J* 15, O-CH_2_), 4.69 (1H, d, *J* 15, O-CH_2_), 4.60−4.52 (1H, m, CH), 4.41−4.34 (1H, m, CH), 4.30−4.21 (1H, m, CH), 3.61 (3H, s, O-CH_3_), 3.04 (1H, dd, *J* 14, 4, CH_2_), 2.78 (1H, dd, *J* 14, 10, CH_2_), 2.36−2.25 (2H, m, S-CH_2_-CH_2_), 1.95 (3H, s, S-CH_3_), 1.89−1.71 (2H, m, S-CH_2_), 1.28 (3H, d, *J* 7, CH_3A_). *d*_C_ (100 MHz, DMSO-d_6_) 195.6, 172.9, 170.9, 170.6, 167.3, 157.7, 137.6, 137.6, 136.4, 132.4, 132.3, 131.5, 131.3, 129.6, 129.2, 128.6, 128.0, 127.4, 127.3, 126.3, 126.0, 119.6, 107.3, 66.8, 53.4, 51.9, 51.8, 47.7, 37.3, 31.8, 29.4, 16.8, 14.5. HRMS (ESI-MS) *m/z*: [M + Na]^+^ calcd for C_37_H_39_N_3_NaO_7_S: 670.2581; found: 670.2590.

**Compound 19**. *d*_H_ (400 MHz, DMSO-d_6_) 12.63 (1H, bs, COOH), 8.30 (1H, d, *J* 7, NH), 8.27 (1H, d, *J* 1, H_Ar_), 8.24 (1H, d, *J* 8, NH), 8.13 (1H, d, *J* 8, NH), 8.06 (1H, d, *J* 9, H_Ar_), 7.94 (1H, d, *J* 9, H_Ar_), 7.84 (1H, dd, *J* 9, 2, H_Ar_), 7.81−7.76 (2H, m, H_Ar_), 7.72−7.67 (1H, m, H_Ar_), 7.62−7.56 (2H, m, H_Ar_), 7.39 (1H, d, *J* 2, H_Ar_), 7.33 (1H, dd, *J* 9, 3, H_Ar_), 7.28−7.18 (4H, m, H_Ar_), 7.18−7.11 (1H, m, H_Ar_), 4.74 (1H, d, *J* 15, O-CH_2_), 4.69 (1H, d, *J* 15, O-CH_2_), 4.60−4.52 (1H, m, CH), 4.41−4.33 (1H, m, CH), 4.23−4.14 (1H, m, CH), 3.06 (1H, dd, *J* 14, 4, CH_2_), 2.77 (1H, dd, *J* 14, 10, CH_2_), 2.35−2.27 (2H, m, S-CH_2_-CH_2_), 1.95 (3H, s, S-CH_3_), 1.92−1.71 (2H, m, S-CH_2_), 1.28 (3H, d, *J* 7, CH_3_). *d*_C_ (125 MHz, DMSO-d_6_) 195.6, 174.0, 170.7, 170.6, 167.3, 157.7, 137.7, 137.6, 136.4, 132.4, 132.3, 131.5, 131.4, 129.6, 129.2, 128.6, 128.0, 127.4, 127.3, 126.2, 126.0, 119.6, 107.3, 66.8, 53.5, 51.8, 47.6, 37.3, 31.8, 29.4, 17.1, 14.6. HRMS (ESI-MS) *m/z*: [M + H]^+^ calcd for C_36_H_38_N_3_O_7_S: 656.2425; found: 656.2435.

Compound **21** was prepared as follows. 4-Trifluoromethylphenol was coupled with chloro-tert-butylacetate followed by deprotection with TFA in chloroform following our established procedures^2^. Phenylalanine methyl ester was coupled to the resulting carboxylic acid, followed by deprotection with LiOH in THF/water following our established procedures^2^. The resulting product was then coupled to IFOEt.TFA (synthesised as described elsewhere^11^), followed by deprotection using LiOH in THF/water following our established protocols^2^.

**CF_3_PhCH_2_COO^t^Bu**. *d*_H_ (400 MHz, CDCl_3_) 7.55 (2H, d, *J* 9, H_Ar_-C-CF_3_), 6.95 (2H, d, *J* 9, O-C-H_Ar_), 4.56 (2H, s, CH_2_), 1.49 (9H, s, CH_3_). *d*_C_ (100 MHz, CDCl_3_) 167.3, 160.3, 126.9 (1C, q, ^3^*J*_C-F_ 4), 124.8 (1C, q, ^1^*J*_C-F_ 271), 123.6 (1C, q, ^2^*J*_C-F_ 33), 114.5, 82.7, 65.5, 27.9. HRMS (ESI-MS) *m/z*: [M + H]^+^ calcd for C_13_H_16_F_3_O_3_: 277.1046 (100%); found: 277.1048 (100%).

**CF_3_PhOH**. *d*_H_ (400 MHz, CDCl_3_) 12.52 (1H, bs, COOH), 7.65 (2H, d, *J* 8, CF_3_-C-CH_Ar_), 7.10 (2H, d, *J* 8, O-C-CH_Ar_), 4.79 (2H, s, CH_2_). *d*_C_ (100 MHz, CDCl_3_) 169.7, 160.6, 126.9 (1C, q, ^3^*J*_C-F_ 4), 125.8 (1C, t, ^1^*J*_C-F_ 271), 121.5 (1C, q, ^2^*J*_C-F_ 32), 115.0, 64.5. HRMS (ESI-MS) *m/z*: [M − H]^−^ calcd for C_9_H_6_F_3_O_3_: 219.0275 (100%); found: 219.0276 (100%).

**CF_3_PhFOMe**. *d*_H_ (400 MHz, DMSO-d_6_) 8.58 (1H, d, *J* 8, NH), 7.62 (2H, d, *J* 9, H_Ar_-C-CF_3_), 7.28−7.17 (5H, m, H_Ar_), 7.01 (2H, d, *J* 9, H_Ar_-C-O), 4.64−4.53 (3H, m, O-CH_2_ and CH), 3.63 (3H, s, CH_3_), 3.10 (1H, dd, *J* 14, 5, CH_2_), 2.98 (1H, dd, J 14, 10, CH_2_). *d*_C_ (100 MHz, DMSO-d_6_) 171.6, 167.3, 160.5, 137.1, 129.0, 128.2, 126.8 (1C, q, ^3^*J*_C-F_ 4), 126.5,124.5 (1C, d, ^1^*J*_C-F_ 271), 121.6 (1C, q, ^2^*J*_C-F_ 32), 115.1, 66.5, 53.2, 52.0, 36.3. HRMS (ESI-MS) *m/z*: [M + H]^+^ calcd for C_19_H_19_F_3_NO_4_: 382.1261 (100%); found: 382.1269 (100%).

**CF_3_PhFOH**. *d*_H_ (400 MHz, DMSO-d_6_) 12.88 (1H, s, COOH), 8.40 (1H, d, *J* 8, NH), 7.61 (2H, d, *J* 9, CH_Ar_-C-CF_3_), 7.27−7.17 (5H, m, H_Ar_), 7.01 (2H, d, J 9, CH_Ar_-C-O), 4.58 (2H, s, O-CH_2_), 4.51 (1H, ddd, *J* 10, 8, 5, CH), 3.12 (1H, dd, *J* 14, 5, CH_2_), 2.96 (1H, dd, *J* 14, 10, CH_2_). *d*_C_ (100 MHz, DMSO-d_6_) 172.6, 167.1, 160.5, 137.5, 129.1, 128.2, 126.8 (1C, q, ^3^*J*_C-F_, 4), 126.4, 124.5 (1C, d, ^1^*J*_C-F_ 271), 121.6 (1C, q, ^2^J_C-F_ 32), 115.1, 66.5, 53.1, 36.4. HRMS (ESI-MS) *m/z*: [M + H]^+^ calcd for C_18_H_17_F_3_NO_4_: 368.1104 (100%); found: 368.1110 (100%).

**CF_3_PhFIFOMe**. *d*_H_ (400 MHz, DMSO-d_6_) 8.47 (1H, d, *J* 7, NH), 8.23 (1H, d, *J* 8, NH), 8.00 (1H, d, *J* 9, NH), 7.58 (2H, d, *J* 8, CF_3_-C-CH_Ar_), 7.28−7.14 (10H, m, H_Ar_), 6.96 (2H, d, *J* 8, CH_Ar_-C-O), 4.65 (1H, ddd, *J* 10, 8.5, 4, CH), 4.54 (2H, s, O-CH_2_), 4.52−4.47 (1H, m, CH), 4.24 (1H, dd, *J* 9, 7.5, CH), 3.56 (3H, s, O-CH_3_), 3.04 (1H, dd, *J* 14, 6, CH_2_), 2.99−2.90 (2H, m, CH_2_), 2.78 (1H, dd, *J* 14, 10, CH_2_), 1.74−1.62 (1H, m, CH-CH_3_), 1.44−1.32 (1H, m, CH_2_-CH_3_), 1.08−0.94 (1H, m, CH_2_-CH_3_), 0.83−0.75 (3H, m, CH_3_). *d*_C_ (100 MHz, DMSO-d_6_) 171.7, 170.9, 170.5, 166.8, 160.5, 137.6, 137.1, 129.2, 128.9, 128.2, 127.9, 126.8 (1C, q, ^3^*J*_C-F_ 4), 126.5, 126.2, 124.5 (1C, d, ^1^*J*_C-F_ 271), 121.7, 115.0, 66.6, 56.5, 53.44, 53.36, 51.7, 37.4, 37.0, 36.5, 24.0, 15.0, 10.9. HRMS (ESI-MS) *m/z*: [M + Na]^+^ calcd for C_34_H_38_F_3_N_3_NaO_6_: 664.2605 (100%); found: 664.2630 (100%).

**Compound** **21**. *d*_H_ (400 MHz, DMSO-d_6_) 12.69 (1H, bs, COOH), 8.27−8.22 (2H, m, NH), 8.01 (1H, d, *J* 9, NH), 7.57 (2H, d, *J* 8, CF_3_-C-CH_Ar_), 7.27−7.13 (10H, m, H_Ar_), 6.96 (2H, d, *J* 8, CH_Ar_-C-O), 4.65 (1H, ddd, *J* 10, 8, 4, CH), 4.54 (2H, s, O-CH_2_), 4.46 (1H, ddd, *J* 9, 8, 5, CH), 4.23 (1H, dd, *J* 9, 7, CH), 3.06 (1H, dd, *J* 14, 5, CH_2_), 2.97−2.88 (2H, m, CH_2_), 2.78 (1H, dd, *J* 14, 10, CH_2_), 1.74−1.64 (1H, m, CH-CH3), 1.44−1.34 (1H, m, CH_2_-CH_3_), 1.06−0.95 (1H, m, CH_2_-CH_3_), 0.83−0.74 (6H, m, CH_3_). *d*_C_ (100 MHz, DMSO-d_6_) 172.7, 170.8, 170.5, 166.8, 160.5, 137.6, 137.5, 129.2, 129.0, 128.1, 127.9, 126.8 (1C, q, ^3^*J*_C-F_ 4), 126.3, 126.1, 124.5 (1C, q, ^1^*J*_C-F_ 271, 121.5 (1C, q, ^2^*J*_C-F_ 32), 115.0, 66.6, 56.6, 53.35, 53.27, 37.4, 37.0, 36.6, 24.1, 15.1, 11.0. HRMS (ESI-MS) *m/z*: [M + Na]^+^ calcd for C_33_H_36_F_3_N_3_NaO_6_: 650.2448 (100%); found: 650.2455 (100%).

Compound **28** was prepared by the coupling of IFOMe.TFA with 4Cl1NapL (both synthesised as described elsewhere^11^), followed by deprotection using LiOH in THF/water following our established protocols^2^.

**4Cl1NapLIFOMe.** *d*_H_ (400 MHz, DMSO-d_6_) 8.46 (1H, d, *J* 7, NH), 8.33 (1H, d, *J* 8, H_Nap_), 8.27 (1H, d, *J* 8.5, H_Nap_), 8.12 (1H, d, *J* 8.5, NH), 7.86 (1H, d, *J* 9, NH), 7.73 (1H, ddd, *J* 8, 7, 1, H_Nap_), 7.65 (1H, ddd, *J* 8, 7, 1, H_Nap_), 7.56 (1H, d, *J* 8, H_Nap_), 7.29−7.13 (5H, m, H_Ar_), 6.88 (1H, d, *J* 8, H_Nap_), 4.81−4.73 (2H, m, O-CH_2_), 4.51−4.41 (2H, m, CH), 4.22 (1H, t, *J* 8, CH), 3.55 (3H, s, O-CH_3_), 3.02 (1H, dd, *J* 14, 6, CH_2_), 2.93 (1H, dd, *J* 14, 9, CH_2_), 1.72−1.61 (1H, m, CH), 1.59−1.43 (2H, m, CH_2_ and/or CH), 1.43−1.30 (2H, m, CH_2_ and/or CH), 1.06−0.92 (1H, m, CH_2_), 0.87−0.72 (12H, m, CH_3_). *d*_C_ (100 MHz, DMSO-d_6_) 171.7, 171.4, 171.0, 170.0, 152.7, 137.1, 130.4, 128.9, 128.2, 128.1, 126.5, 126.4, 126.1, 125.9, 123.6, 122.5, 122.3, 106.1, 67.4, 56.3, 53.4, 51.7, 50.8, 40.8, 36.9, 36.4, 24.2, 24.0, 23.1, 21.5, 15.0, 10.9. HRMS (ESI-MS) *m/z*: [M + Na]^+^ calcd for C_34_H_41_ClN_3_NaO_6_: 646.2654 (100%); found: 646.2671 (100%).

**Compound 28**. *d*_H_ (400 MHz, DMSO-d_6_) 12.80 (1H, bs, COOH), 8.33 (1H, d, *J* 8, H_Ar_), 8.28 (1H, d, *J* 8, NH), 8.19 (1H, d, *J* 8, NH), 8.12 (1H, d, *J* 8, H_Ar_), 7.88 (1H, d, *J* 9, NH), 7.73 (1H, ddd, *J* 8, 7, 1, H_Ar_), 7.65 (1H, ddd, *J* 8, 7, 1, H_Ar_), 7.56 (1H, d, *J* 8, H_Ar_), 7.26−7.12 (5H, m, H_Ar_), 6.88 (1H, d, *J* 8, H_Ar_), 4.79 (1H, d, *J* 15, O-CH_2_), 4.75 (1H, d, *J* 14.5, O-CH_2_), 4.49−4.36 (2H, m, CH), 4.20 (1H, dd, *J* 9, 8, CH), 3.05 (1H, dd, *J* 14, 5, CH_2_), 2.89 (1H, dd, *J* 14, 9, CH_2_), 1.75−1.61 (1H, m, CH), 1.59-1.44 (2H, m, CH_2_ and/or CH), 1.44-1.30 (2H, m, CH_2_ and/or CH), 1.06-0.91 (1H, m, CH_2_), 0.84 (3H, d, *J* 6.5, CH_3_), 0.82 (3H, d, *J* 6.5, CH_3_), 0.77 (3H, d, *J* 6.8, CH-CH_3_), 0.75 (3H, t, *J* 7, CH_2_-CH_3_). *d*_C_ (100 MHz, DMSO-d_6_) 172.7, 171.3, 170.8, 167.0, 152.7, 137.6, 130.4, 129.0, 128.1, 128.1, 126.4, 126.3, 126.1, 125.9, 123.6, 122.5, 122.3, 106.1, 67.4, 56.6, 53.4, 50.8, 40.8, 36.9, 36.6, 24.2, 24.1, 23.1, 21.5, 15.2, 10.9. HRMS (ESI-MS) *m/z*: [M + Na]^+^ calcd for C_33_H_40_ClN_3_NaO_6_: 632.2498 (100%); found: 663.2488 (100%).

Compound **30** was synthesised as follows. BocV was coupled with FOMe.HCl followed by deprotection with TFA following our established methods to give VFOMe.TFA. 1-Bromo-2-naphthoxyacetic acid was coupled with VFOMe.TFA to give 1Br-2-NapVFOMe, followed by deprotection with LiOH in THF/water following our established protocols^2^.

**BocVFOMe**. *d*_H_ (400 MHz, DMSO-d_6_) 8.25 (0.9H, d, *J* 8, NH), 7.63 (0.1H, d, *J* 8, NH), 7.32−7.13 (5H, m, H_Ar_), 6.56 (0.9H, d, *J* 9, NH), 6.18 (0.1H, bs, NH), 4.49 (1H, td, *J* 9, 6, CH), 3.78 (1H, dd, *J* 9, 7, CH), 3.61 (0.5H, s, O-CH_3_), 3.57 (2.5H, s, O-CH_3_), 3.02 (1H, dd, *J* 14, 6, CH_2_), 2.92 (1H, dd, *J* 14, 9, CH_2_), 1.90−1.73 (1H, m, CH-CH_3_), 1.37 (7.5H, s, CH_3_), 1.30 (1.5H, s, CH_3_), 0.83 (0.5H, dd, *J* 7, 3, CH_3_), 0.76 (5.5H, dd, *J* 7, 3, CH_3_). *d*_C_ (100 MHz, DMSO-d_6_) 171.8, 171.4, 155.2, 137.1, 129.0, 128.2, 126.5, 78.0, 59.5, 53.4, 51.7, 36.6, 30.5, 28.2, 19.0, 18.1. HRMS (ESI-MS) *m/z*: [M + Na]^+^ calcd for C_20_H_30_N_2_NaO_5_: 401.2047 (100%); found: 401.2058 (100%).

**VFOEt.TFA.** *d*_H_ (400 MHz, DMSO-d_6_) 8.87 (1H, d, *J* 7, NH), 8.07 (3H, bs, NH_3_), 7.33−7.21 (5H, m, H_Ar_), 4.56 (1H, ddd, *J* 8.5, 7, 6, CH), 3.64 (1H, d, *J* 5, CH), 3.60 (3H, s, O-CH_3_), 3.07 (1H, dd, *J* 14, 6, CH_2_), 2.98 (1H, dd, *J* 14, 9, CH_2_), 2.17−2.06 (1H, m, CH-CH_3_), 0.95 (3H, d, *J* 7, CH_3_), 0.91 (3H, d, *J* 7, CH_3_). *d*_C_ (100 MHz, DMSO-d_6_) 171.3, 168.2, 136.7, 129.0, 128.3, 126.7, 57.0, 53.9, 51.9, 36.4, 29.8, 18.2, 17.1. HRMS (ESI-MS) *m/z*: [M + H]^+^ calcd for C_15_H_23_N_2_O_3_: 279.103 (100%); found: 279.1710 (100%).

**1Br2NapVFOMe**. *d*_H_ (400 MHz, DMSO-d_6_) 8.61 (1H, d, *J* 7, NH), 8.10 (1H, d, *J* 8.5, H_Nap_), 7.98 (1H, d, *J* 9, H_Nap_), 7.95 (1H, d, *J* 8.5, H_Nap_), 7.82 (1H, d, *J* 9, NH), 7.65 (1H, ddd, *J* 8.5, 7, 1, H_Nap_), 7.48 (1H, ddd, *J* 8, 7, 1, H_Nap_), 7.42 (1H, d, *J* 9, H_Nap_), 7.29−7.16 (5H, m, H_Ar_), 4.87−4.78 (2H, m, O-CH_2_), 4.49 (1H, ddd, *J* 9, 7, 6, CH), 4.35 (1H, dd, *J* 9, 6, CH), 3.57 (1H, dd, *J* 14, 5, CH_3_), 3.04 (1H, dd, *J* 14, 6, CH_2_), 2.93 (1H, dd, *J* 14, 9, CH_2_), 2.01 (1H, oct., *J* 7, CH-CH_3_), 0.85 (3H, d, *J* 7, CH_3_), 0.80 (3H, d, *J* 7, CH_3_). *d*_C_ (100 MHz, DMSO-d_6_) 171.8, 170.6, 166.9, 152.1, 137.1, 132.2, 129.7, 129.3, 129.0, 128.4, 128.3, 126.6, 125.3, 124.7, 115.2, 107.5, 67.9, 56.4, 53.6, 51.8, 36.5, 31.2, 19.1, 17.5. HRMS (ESI-MS) *m/z*: [M + H]^+^ calcd for C_27_H_29_BrN_2_NaO_5_: 563.1152 (100%); found: 563.1158 (100%).

**Compound 30**. *d*_H_ (400 MHz, DMSO-d_6_) 12.70 (1H, bs, COOH), 8.43 (1H, d, *J* 8, NH), 8.10 (1H, d, *J* 9, H_Nap_), 7.98 (1H, d, *J* 9, H_Nap_), 7.95 (1H, d, *J* 8, H_Nap_), 7.80 (1H, d, *J* 9, NH), 7.65 (1H, ddd, *J* 8, 7, 1, H_Nap_), 7.48 (1H, ddd, *J* 8, 7, 1, H_Nap_), 7.43 (1H, d, *J* 9, H_Nap_), 7.28−7.15 (5H, m, H_Ar_), 4.87−4.77 (2H, m, O-CH_2_), 4.45 (1H, td, *J* 8, 5, CH), 4.36 (1H, dd, *J* 9, 6, CH), 3.07 (1H, dd, *J* 14, 5, CH_2_), 2.89 (1H, dd, *J* 14, 9, CH_2_), 2.02 (1H, oct., *J* 6, CH-CH_3_), 0.85 (3H, d, *J* 7, CH_3_), 0.80 (3H, d, *J* 7, CH_3_). *d*_C_ (100 MHz, DMSO-d_6_) 172.7, 170.4, 166.8, 152.1, 137.5, 132.1, 129.7, 129.3, 129.0, 128.3, 128.2, 128.1, 126.4, 125.2, 124.7, 115.2, 107.5, 67.9, 56.4, 53.4, 36.5, 31.2, 19.1, 17.4. HRMS (ESI-MS) *m/z*: [M + H]^+^ calcd for C_26_H_27_BrN_2_O_5_: 529.1160 (100%); found: 529.1163 (98%).

Compound **31** was synthesised as follows. IFOMe.TFA (prepared as described for IFOEt.TFA elsewhere^11^) was coupled with 7-methoxy-2-naphthoxyacetic acid, followed by deprotection with LiOH in THF/water followed our established protocols^2^.

**7OMe2NapIFOMe**. *d*_H_ (400 MHz, DMSO-d_6_) 8.57 (1H, d, *J* 7, NH), 7.90 (1H, d, *J* 9, NH), 7.76 (1H, d, *J* 6, H_Nap_), 7.73 (1H, d, *J* 6, H_Nap_), 7.26−7.12 (7H, m, H_Nap_ and H_Ar_), 7.04 (1H, dd, *J* 9, 2.5, H_Nap_), 7.00 (1H, dd, *J* 9, 2.5, H_Nap_), 4.69−4.60 (2H, m, O-CH_2_), 4.48 (1H, ddd, *J* 9, 7, 6, CH), 4.32 (1H, dd, *J* 9, 7, CH), 3.84 (3H, s, O-CH_3_), 3.55 (3H, s, O-CH_3_), 3.02 (1H, dd, *J* 13, 6, CH_2_), 2.92 (1H, dd, *J* 14, 9, CH_2_), 1.79−1.68 (1H, m, CH-CH_3_), 1.36 (1H, dtt, *J* 15, 7, 4, CH_2_-CH_3_), 1.06−0.95 (1H, m, CH_2_-CH_3_), 0.80 (3H, d, *J* 7, CH-CH_3_), 0.76 (3H, t, *J* 7, CH_2_-CH_3_). *d*_C_ (100 MHz, CDCl_3_) 171.7, 170.8, 167.2, 157.8, 156.1, 137.0, 135.5, 129.1, 129.0, 128.9, 128.2, 126.5, 124.0, 116.1, 115.7, 106.8, 105.3, 66.7, 56.0, 55.0, 53.5, 51.7, 36.9, 36.4, 24.0, 15.1, 10.9. HRMS (ESI-MS) *m/z*: [M + Na]^+^ calcd for C_29_H_34_N_2_NaO_6_: 529.2309 (100%); found: 529.2319 (100%).

**Compound 31**. *d*_H_ (400 MHz, DMSO-d_6_) 12.70 (1H, bs, COOH), 8.43 (1H, d, *J* 8, NH), 7.92 (1H, d, *J* 9, NH), 7.74 (2H, dd, *J* 9, 6, H_Nap_), 7.24−7.12 (7H, m, H_Nap_ and H_Ar_), 7.04 (1H, dd, *J* 8, 2.5, H_Nap_), 7.00 (1H, dd, *J* 9, 2.5, H_Nap_), 4.68−4.59 (2H, m, O-CH_2_), 4.43 (1H, ddd, *J* 9, 8, 5, CH), 4.31 (1H, dd, *J* 9, 7, CH), 3.83 (3H, s, O-CH_3_), 3.04 (1H, dd, *J* 14, 5, CH_2_), 2.87 (1H, dd, *J* 14, 9, CH_2_), 1.79−1.69 (1H, m, CH), 1.41−1.31 (1H, m, CH_2_), 1.04−0.92 (1H, m, CH_2_), 0.79 (3H, d, *J* 7, CH-CH_3_), 0.74 (3H, t, *J* 7, CH_2_-CH_3_). *d*_C_ (100 MHz, DMSO-d_6_) 172.8, 170.8, 167.2, 157.8, 156.1, 137.5, 135.5, 129.2, 129.0, 128.1, 126.4, 124.1, 116.1, 115.7, 106.8, 105.3, 66.7, 56.2, 55.1, 53.4, 37.0, 36.6, 24.0, 15.2, 10.9. HRMS (ESI-MS) *m/z*: [M + Na]^+^ calcd for C_28_H_32_N_3_NaO_6_: 515.2153 (100%); found: 515.2162 (100%).

Compound **34** was synthesised following a slightly modified procedure. First, DBS-CO_2_Me was prepared by coupling methyl 4-formylbenzoate and D-sorbitol following an established procedure.^13^  Additional purification was achieved by sonicating the crude solid in HCl (1M) for 1 h, followed by collection at the filter and washing with water. The solid was then sonicated in acetone for 1 h, and the now white solid collected at the filter, washed with water and acetone, and dried to give DBS-CO_2_Me. This was deprotected following an established procedure.^13^ Additional purification was achieved by dissolving the solid in boiling DMF and precipitating from acetone. The precipitate was collected at the filter, washed with acetone, and dried to give the crude product. The crude solid was sonicated in MeCN, collected at the filter, washed with MeCN, and dried to give **DBS-CO_2_H** as a white solid.

**DBS-CO_2_Me**. *d*_H_ (400 MHz, DMSO-d_6_) 7.99 (4H, dd, *J* 8, 3, H_Ar_), 7.62 (4H, t, *J* 8, H_Ar_), 5.77 (2H, s, Ar-CH), 4.93 (1H, d, *J* 6, CH-OH), 4.46 (1H, t, *J* 6, CH_2_-OH), 4.29–4.17 (3H, m, CH-CH-CH-OH and Ar-CH-O-CH_2_), 4.02 (1H, d, *J* 2, CH), 3.90 (1H, dd, *J* 9, 2, CH-CH-OH), 3.86 (6H, s, CH_3_), 3.79 (1H, dtd, *J* 8, 5, 1, CH-OH), 3.63 (1H, ddd, *J*, 11, 6, 2, 1/2CH_2_-OH), 3.47 (1H, quin. *J* 6, 1/2CH_2_-OH). *d*_C_ (101 MHz, DMSO-d_6_) 166 (2C, C=O), 143.3 (C_Ar_), 143.0 (C_Ar_), 129.75 (C_Ar_), 129.70 (C_Ar_), 129.0 (CH_Ar_), 128.9 (CH_Ar_), 126.5 (2C, CH_Ar_), 98.5 (Ar-CH), 98.3 (Ar-CH), 77.6 (CH-CH-OH), 70.1 (CH), 69.3 (Ar-CH-O-CH_2_), 68.5 (CH-CH-CH-OH), 67.6 (CH-OH), 62.5 (CH_2_­-OH), 52.1 (2C, CH_3_). HRMS (ESI-QTOF) *m/z*: [M + Na]^+^ calcd for C_24_H_26_NaO_10_ 497.1418 (100%); found: 497.1416 (100%).

**Compound** **34**. *d*_H_ (400 MHz, DMSO-d_6_) 13.02 (2H, bs, COOH), 7.95 (4H, dd, *J* 8, 3, H_Ar_), 7.58 (4H, t, *J* 8, H_Ar_), 5.75 (2H, s, Ar-CH), 4.99 (1H, bs, CH-OH), 4.55 (1H, bs, CH_2_-OH), 4.27–4.16 (3H, m, CH-CH-CH-OH and Ar-CH-O-CH_2_), 4.01 (1H, s, CH), 3.90 (1H, d, *J* 9, CH-CH-OH), 3.78 (1H, bs, CH-OH), 3.62 (1H, bd, *J* 10, 1/2CH_2_-OH), 3.51–3.42 (1H, m, 1/2CH_2_-OH). *d*_C_ (101 MHz, DMSO-d_6_) 167.1 (2C, COOH), 142.9 (C_Ar_), 142.7 (C_Ar_), 131.04 (C_Ar_), 130.98 (C_Ar_), 129.2 (CH_Ar_), 129.1 (CH_Ar_), 126.3 (2C, CH_Ar_), 98.7 (Ar-CH), 98.6 (Ar-CH), 77.6 (CH-CH-OH), 70.2 (CH), 69.4 (Ar-CH-O-CH_2_), 68.6 (CH-CH-CH-OH), 67.7 (CH-OH), 62.6 (CH_2_-OH). HRMS (ESI-QTOF) *m/z*: [M − H]^−^ calcd for C_22_H_21_O_10_ 445.1140 (100%); found 455.1138 (100%).

Compound **35** was synthesised following a slightly modified procedure. First, DBS-CO_2_H was coupled with GOMe.HCl following an established procedure.^13^ Additional purification was achieved by sonicating the crude solid in MeCN, collecting at the filter, washing with MeCN and drying to give DBS-GOMe as a white solid. This was deprotected with LiOH in MeOH/water following our established protocols^2^. Additional purification was achieved by sonicating the crude solid sequentially in MeCN, an MeCN:water mixture (1:1), and MeOH, in which the solid was collected at the filter, washed with MeOH, and dried to give **DBS-G** as a white solid.

**DBS-GOMe**. *d*_H_ (400 MHz, DMSO-d_6_) 8.98 (2H, t, *J* 6, NH), 7.89 (4H, dd, *J* 8, 2, H_Ar_), 7.57 (4H, t, *J* 9, H_Ar_), 5.74 (2H, s, Ar-CH), 4.91 (1H, d, *J* 6, CH-OH), 4.45 (1H, t, *J* 6, CH_2_-OH), 4.27–4.17 (3H, m, CH-CH-CH-OH and Ar-CH-O-CH_2_), 4.02 (5H, d, J 6, CH_2G_ and CH), 3.89 (1H, dd, *J* 9, 2, CH-CH-OH), 3.84–3.76 (1H, m, CH-OH), 3.67–3.59 (7H, m, O-CH_3_ and 1/2CH_2_-OH), 3.47 (1H, quin., *J* 5, 1/2CH_2_-OH). *d*_C_ (100 MHz, DMSO-d_6_) 170.4 (2C, C=O_G_), 166.28 (Ar-C=O), 166.26 (Ar-C=O), 141.7 (C_Ar_), 141.4 (C_Ar_), 133.79 (C_Ar_), 133.72 (C_Ar_), 127.1 (CH_Ar_), 127.0 (CH_Ar_), 126.1 (2C, CH_Ar_), 98.73 (Ar-CH), 98.68 (Ar-CH), 77.6 (CH-CH-OH), 70.2 (CH), 69.3 (Ar-CH-O-CH_2_), 68.5 (CH-CH-CH-OH), 67.6 (CH-OH), 62.6 (CH_2_-OH), 51.7 (2C, O-CH_3_), 41.2 (2C, CH_2G_). HRMS (ESI-QTOF) *m/z*: [M + HCOO]^−^ calcd for C_29_H_33_N_2_O_14_ 633.1937 (100%); found 633.1920 (100%).

**Compound 35**. *d*_H_ (400 MHz, DMSO-d_6_) 12.60 (2H, bs, COOH), 8.86 (2H, t, *J* 6, NH), 7.89 (4H, dd, *J* 8, 2, H_Ar_), 7.56 (4H, t, *J* 9, H_Ar_), 5.74 (2H, s, Ar-CH), 4.91 (1H, bs, CH-OH), 4.45 (1H, bs, CH_2_-OH), 4.25–4.18 (3H, m, CH-CH-CH-OH and Ar-CH-O-CH_2_), 4.00 (1H, s, CH), 3.97–3.86 (5H, m, CH_2G_ and CH-CH-OH), 3.83–3.79 (1H, m, CH-OH), 3.67–3.60 (1H, m, 1/2CH_2_-OH), 3.52–3.43 (1H, m, 1/2CH_2_-OH). *d*_C_ (100 MHz, DMSO-d_6_) 171.4 (2C, COOH), 166.3 (Ar-C=O), 166.2 (Ar-C=O), 141.6 (C_Ar_), 141.4 (C_Ar_), 134.1 (C_Ar_), 134.0 (C_Ar_), 127.1 (CH_Ar_), 127.0 (CH_Ar_), 126.2 (2C, CH_Ar_), 98.84 (Ar-CH), 98.79 (Ar-CH), 77.7 (CH-CH-OH), 70.3 (CH), 69.4 (Ar-CH-O-CH_2_), 68.6 (CH-CH-CH-OH), 67.7 (CH-OH), 62.7 (CH_2_-OH), 41.3 (2C, CH_2G_). HRMS (ESI-QTOF) *m/z*: [M − H]^−^ calcd for C_26_H_27_N_2_O_12_ 559.1569; found 559.1567 (100%).

# Supplementary Notes

**Supplementary Note 1**

The distribution of testing set examples across Tree Manifold Approximation and Projection (TMAP, **Fig. 3** in the main text) differs between data splits – data splits 1 and 2 having test set points concentrated on two of three branches in descriptor space. Data splits 3-6 have comparable coverage of testing set examples over the entire tree manifold. This may represent balance between good test set coverage for model evaluation and diversity within the training set.

We used the Shapiro-Wilk test which tests the null hypothesis that the data is not normally distributed. For the G′ dataset, the Shapiro-Wilk statistic (W) was calculated as 0.99 with a p-value of 0.38. For the G″ dataset, W was calculated as 0.99 with a p-value of 0.65. For both, the p-value is greater than the cut-off of 0.05 indicating a failure to reject the null-hypothesis and a high likelihood that these data are normally distributed. Additionally, plotting of our training set G′ and G″ values in a quantile-quantile (q-q) plot demonstrates a strong linear relationship between our values and theoretical quantiles (R2 = 0.99 for both G′ and G″, **Fig. S3C,D**).

**Supplementary Note 2**

The original paper on BART states that the default parameters of the original implementation could be used without tuning but also states that, if chosen to do so, hyperparameters should be tuned by a cross validated approach^[15]^. There exists literature precedent for tuning of m which has a marked effect on the feature importance within the algorithm^[16,17]^. It is also noted that for small datasets, the value of m should be kept low – for relatively small datasets such as ours, PyMC3 uses a reduced number of trees (m = 50) by default. The importance of tuning α has not widely explored in the literature and there is also no consensus on the necessity of tuning this hyperparameter.

We found that for both datasets average 5xCV RMSE increased with increasing number of trees while training set RMSE was observed to decrease (**Fig. S6,S7**); this can be rationalised by overfitting of the training data with a large number of trees. For 5xCV RMSE, no generalisable trend was seen for alpha-values across different numbers of trees. Trianing set RMSE was observed to decrease with decreasing α suggesting a larger number of shallow trees may lead to overfitting of training data. For our data, however, fitting with low numbers of trees and high values of alpha resulted in underfitting of training data. We therefore restricted choice of hyperparameters to m >= 25 and α ∈ {0.2,0.3,…,0.8} for the final models.

**Supplementary Note 3**

We have used semi-stratified splitting to construct six unique datasets with which to build and tune BART models for the prediction of G′ and G″. In our models, we scale our descriptors by concentration in alignment with the near-linear dependence of LogG′ and LogG″ on concentration of LMWG. We then tuned hyperparameters based on 5xCV of training set examples. To further validate our method of dataset construction and hyperparameter tuning, we investigated method 1) an alternative non-stratified splitting method; method 2) semi-stratified sampling with concentration included as a descriptor; and method 3) alternative semi-stratified splitting into 10 unique training, test and validation sets.

Datasets in the non-stratified models are constructed identically to those described in the main text except LMWG the highest and lowest G′/G″ values are not placed in the training set by default. In models with concentration as a descriptor, dataset construction is identical to the models presented in the main text but descriptor values are not scaled by concentration and concentration is provided as a descriptor for each example. Briefly, for the validation set tuning method we reserve LMWG with highest and lowest values of G′/G″ and split the reduced set of molecules into 10-folds. We use 8 folds to form a training set with reserved LMWG, and the remaining folds for validation and testing sets respectively. We repeat this process such that each fold is used as the validation set and testing set exactly once. For each method, we screened hyperparameters m ∈ {10,15,..,50} and α ∈ {0.1,0.2,…,0.9} and present the best performing model as measured by ether 5xCV RMSE (methods one and two), or validation set RMSE (method three). Metrics arising from these models are presented in **Table S3** and **Table S5**. Plots of predictions and the results of Y-randomisation are presented in **Figures S13-S26.**

For G′, the semi-stratified method yielded the lowest average RMSE (0.438), followed by alternative methods 2 (0.459), 3 (0.476), and 1 (0.483), indicating that semi-stratified dataset construction leads to more generalizable models, with concentration scaling offering no improvement. No individual test set RMSE for G′ was below 0.3 for any alternative methods. For G″, the lowest average test set RMSE was observed with validation set tuning method 3 (0.463), followed by method 2 (0.463) then the semi-stratified method (0.468) and method 1 performing the most poorly (0.482). Two models for G″ (8 and 9), produced by method 3, had RMSE values below 0.3 – compared to only one model by the semi-stratified construction methods. Similar trends to G′ were seen for method 1, where average performance was poorer than the semi-stratified method. Most models produced using concentration as a descriptor had a lower RMSE than equivalent models produced by the main text model though in most cases the difference is small. For all methods, we note that even in models with RMSE > 0.5 we see most of the validation set predictions’ Bayesian confidence interval overlap with the experimental value when plotting predicted versus experimentally derived rheological values. Results of Y-randomisation for each method showed that the performance of the majority of dataset splits were unlikely to be due to change – for method 1 6/6 G′ and 6/6 G″ Y-randomisation studies had a p-value <0.05 while method 2 had 5/6 and 3/6 and method 3 had 4/10 and 5/10, for G′ and G″, respectively. These results are comparable to the main text model.

The best performing models produced by these splitting methods are comparable to our selected semi-stratified method. Furthermore, we observe little effect of inclusion of concentration as a descriptor over our descriptor scaling method. Overall, we believe that our approach to dataset selection and hyperparameter tuning is not inferior any alternative method trialled. We opted to use five-fold cross-validation for hyperparameter tuning, as it enables training the model with more examples compared to a holdout validation set.

**Supplementary Note 4**

The Y randomisation results for G′ show five out of six test set performances had a less than 2% probability of being due to chance while data split 2 had a Z score of 0.29 and an associated p-value of 0.39 suggesting a strong probability the true test set performance could be due to chance. The Y randomisation results for G″ were better with the lowest Z score obtained for data split 4 at 1.13 and a p-value of 0.13 suggesting a 13% probability of the true test set performance being due to chance.

# Supplementary Figures


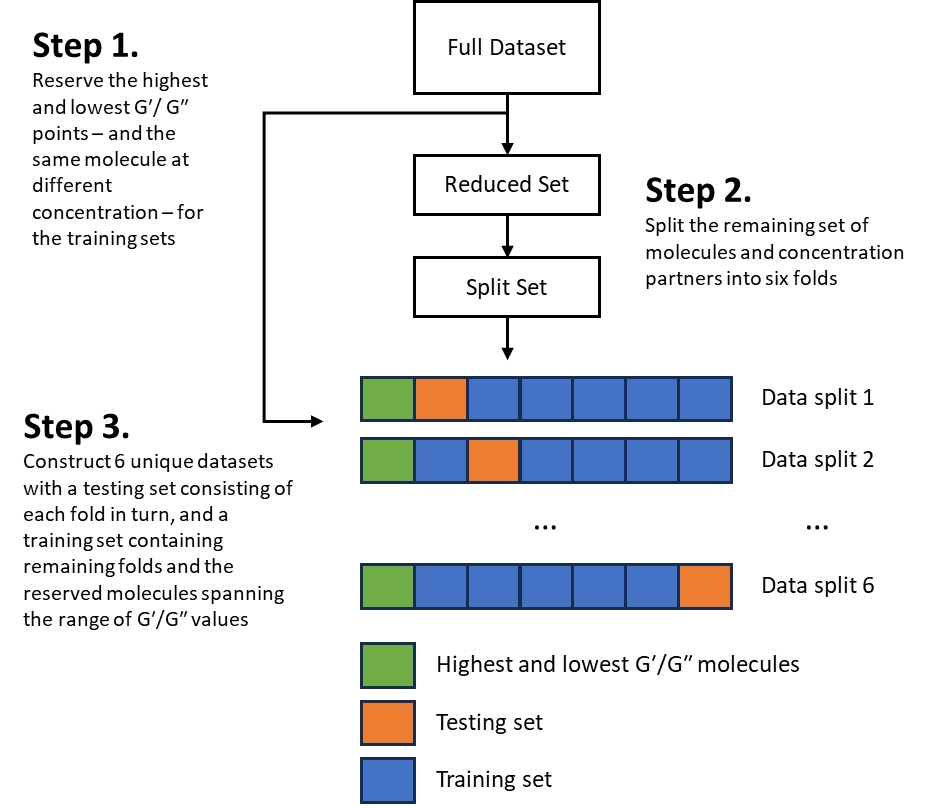
**Figure S2:** Flowchart demonstrating the data splitting procedure carried out in this work.


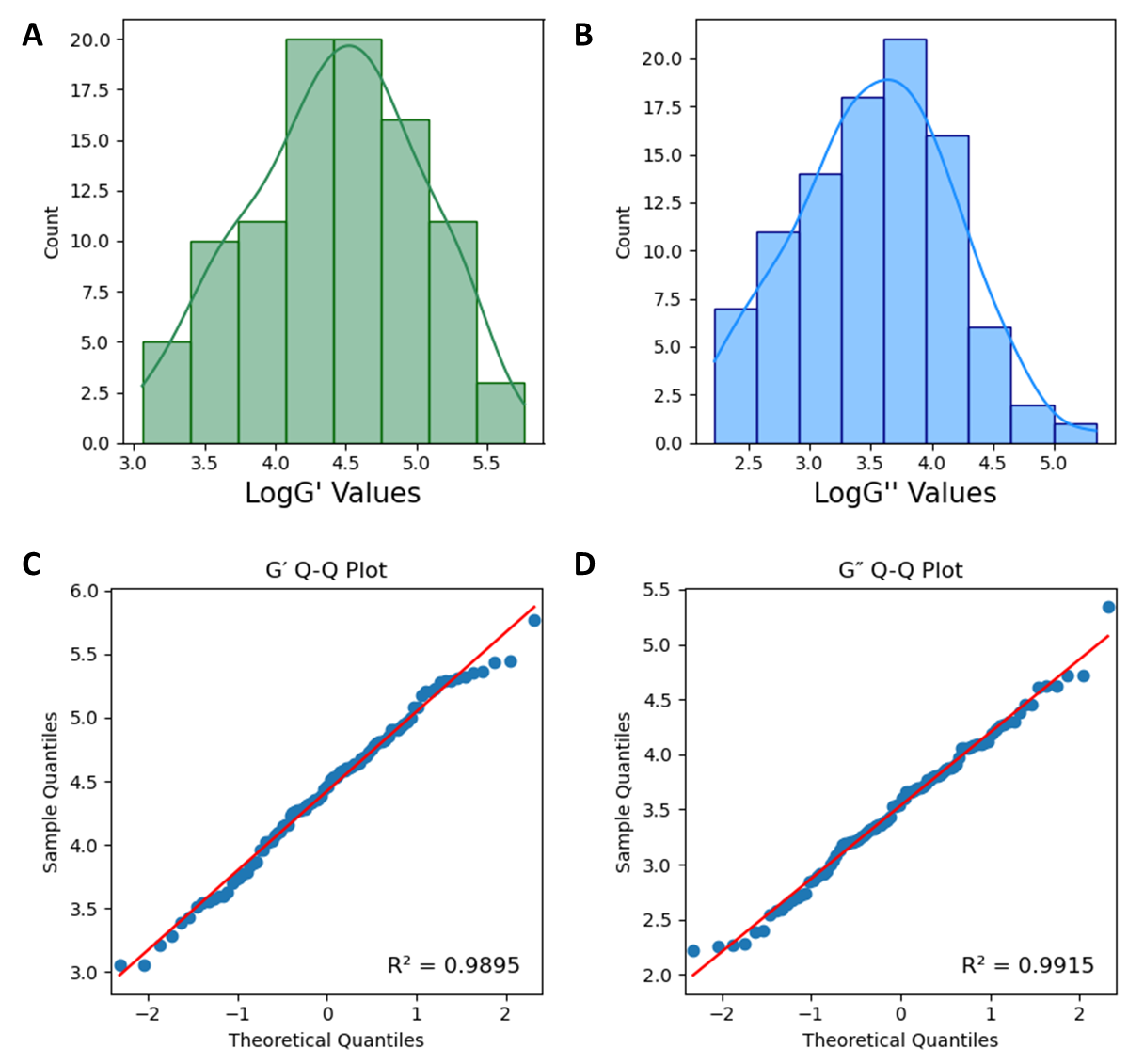


**Figure S3:** Quantile-quantile plots of G′ and G″ values of the training dataset against datapoints drawn from a theoretical normal distribution.


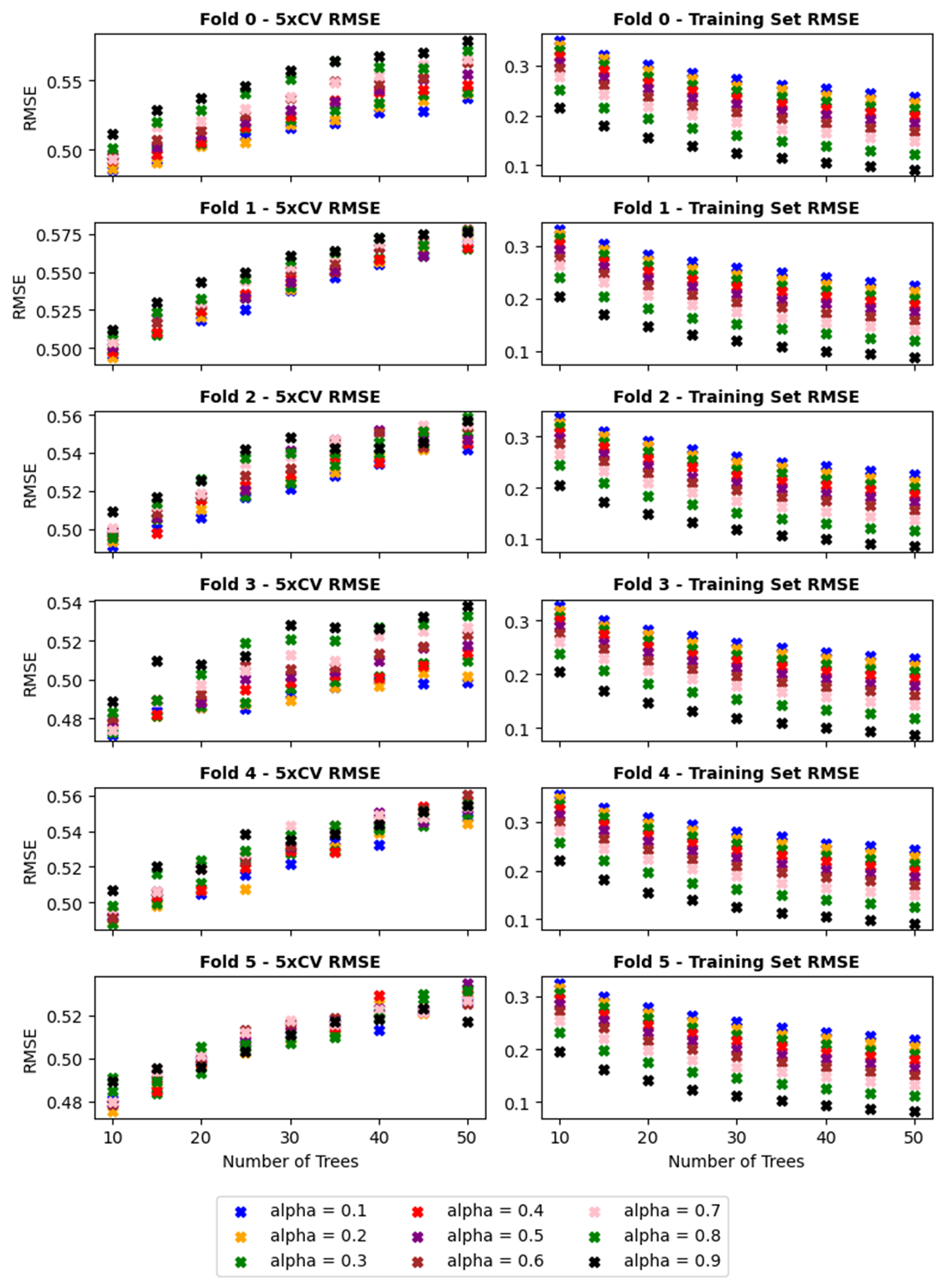


**Figure S4:** Plots summarizing hyperparameter tuning for each of six produced G′ BART models. Plots of average 5-fold Cross Validation (5xCV) RMSE values (left column) and training set RMSE values (right columns) against the hyperparameter “number of trees”. Points are coloured by value of the hyperparameter “alpha”.


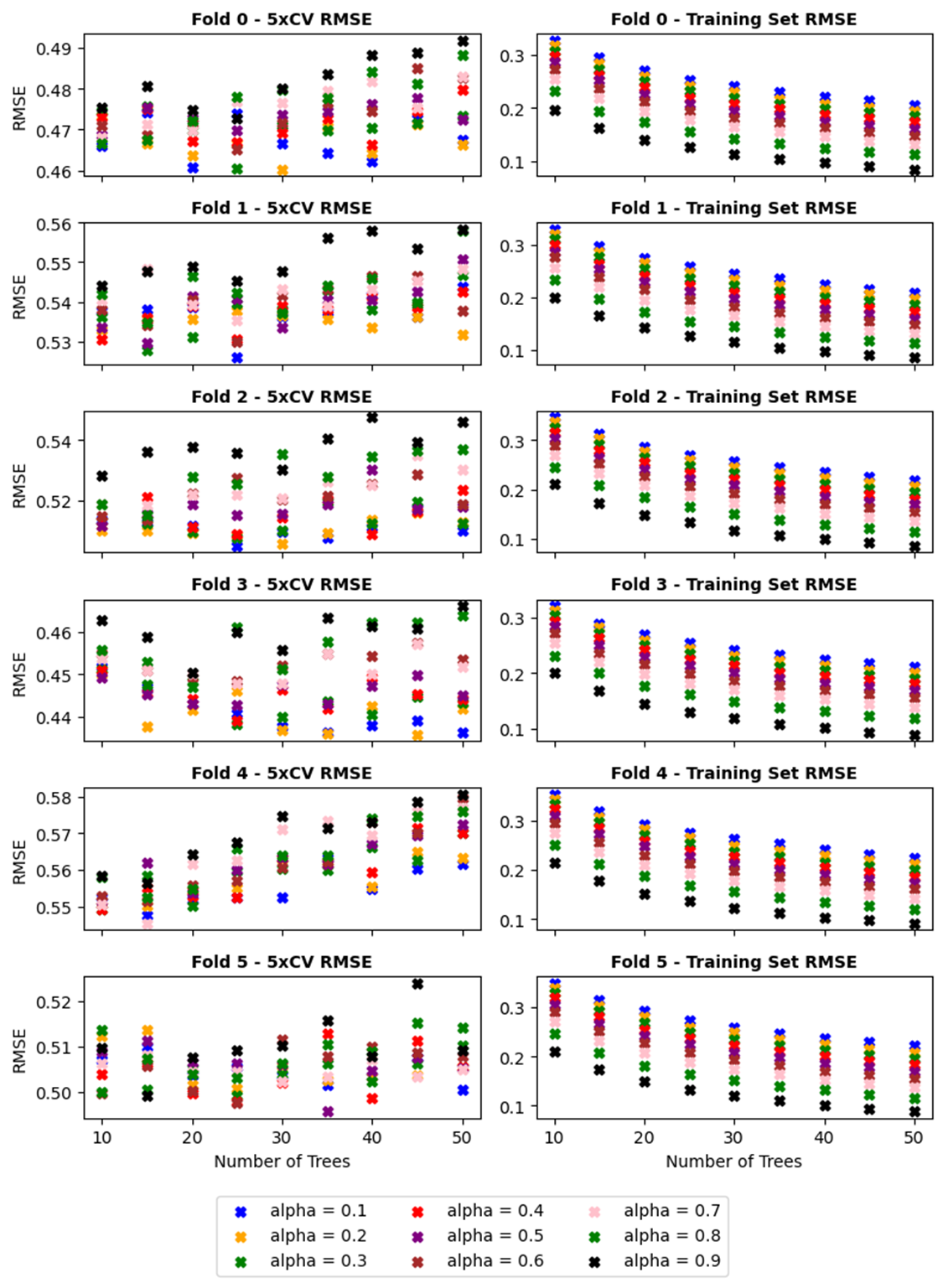


**Figure S5:** Plots summarizing hyperparameter tuning for each of six produced G″ BART models. Plots of average 5-fold Cross Validation (5xCV) RMSE values (left column) and training set RMSE values (right columns) against the hyperparameter “number of trees”. Points are coloured by value of the hyperparameter “alpha”.


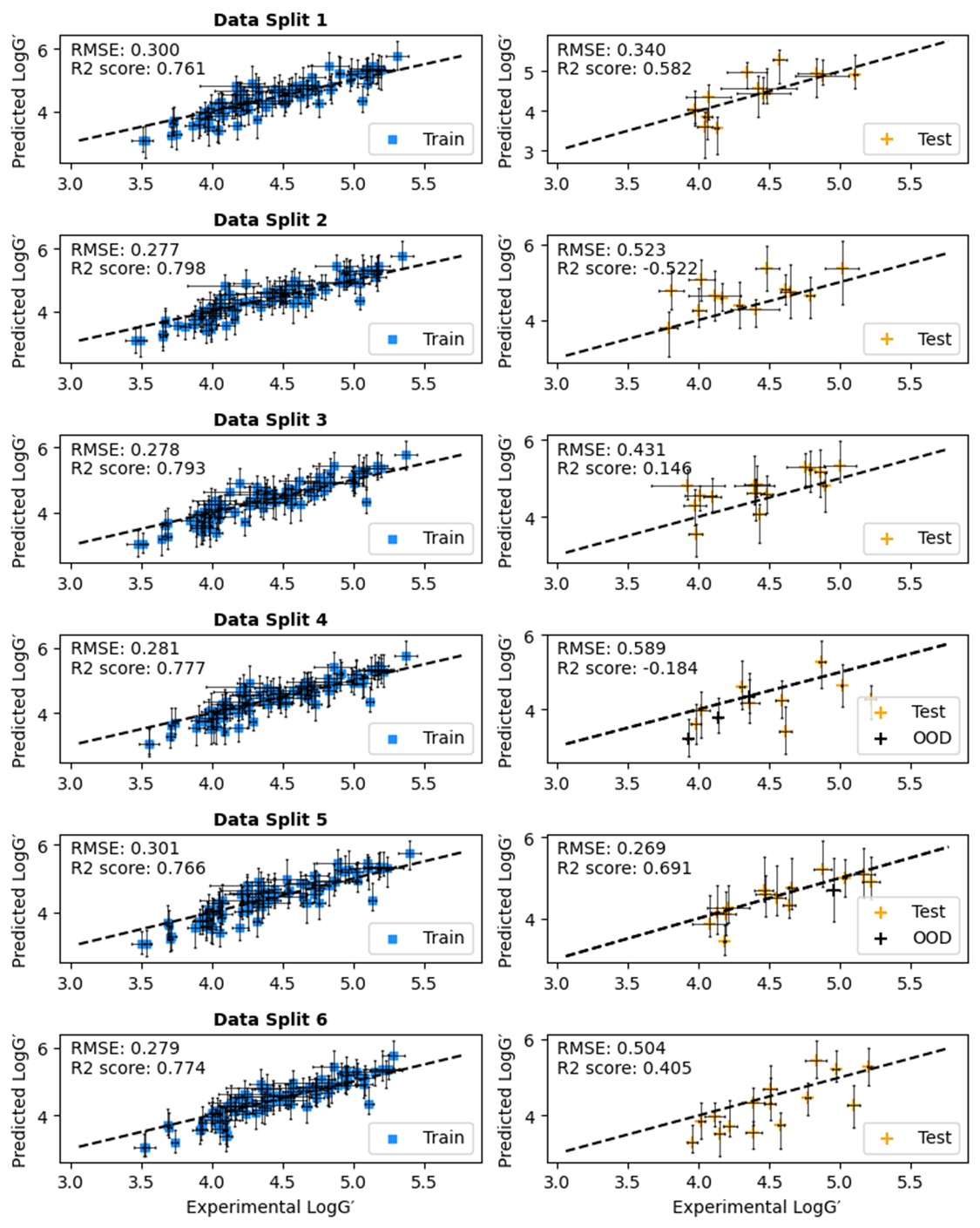


**Figure S6:** Scatterplots of measured rheological values (x-axis) plotted against BART-predicted G′(y-axis) for each of the six models produced from unique data splits. The line y=x is plotted as a dashed black line for reference. Experimental error is indicated by horizontal error bars and the 89% Bayesian credible interval is indicated by vertical error bars.


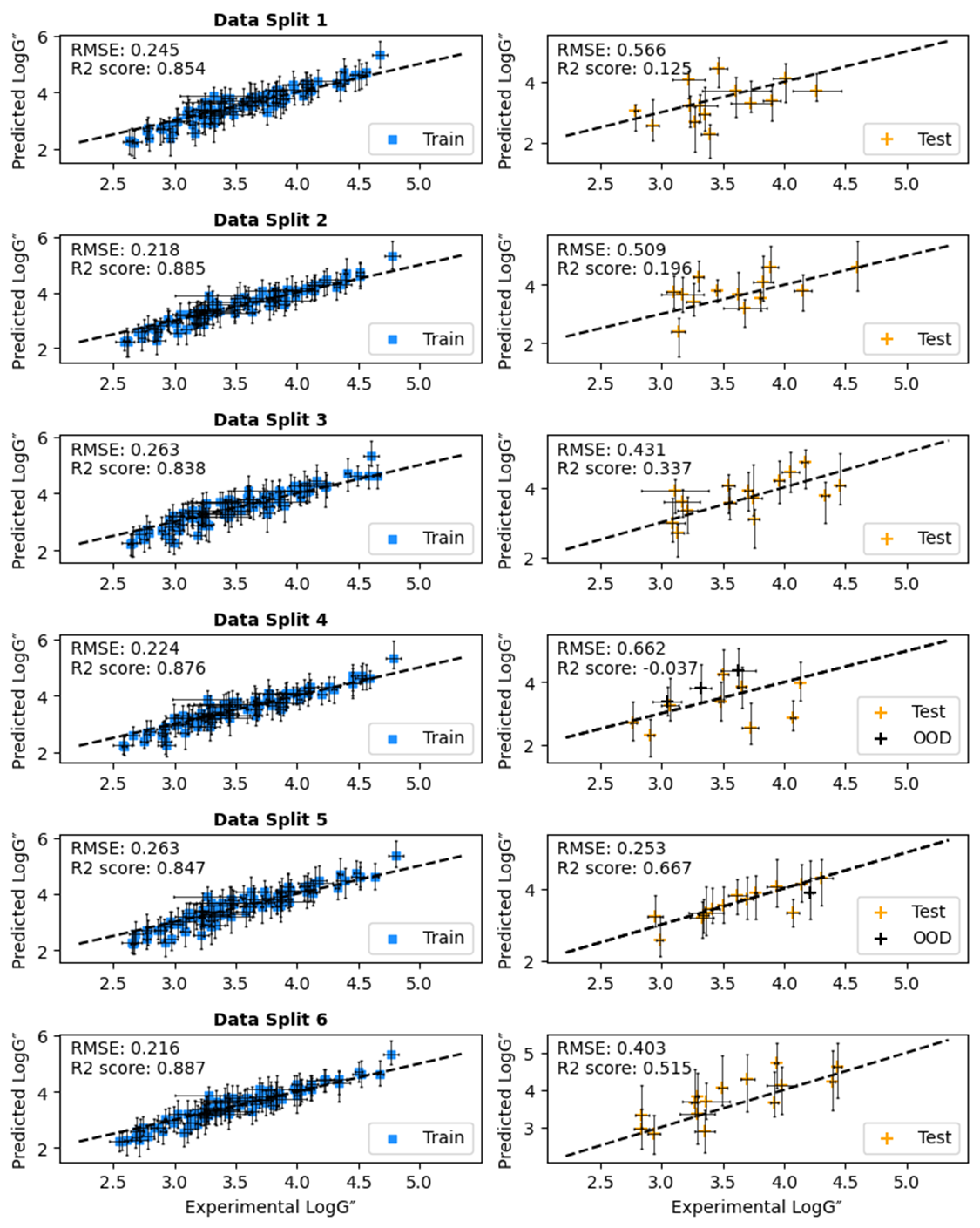


**Figure S7:** Scatterplots of measured rheological values (x-axis) plotted against BART-predicted G″ (y-axis) for each of the six models produced from unique data splits. The line y=x is plotted as a dashed black line for reference. Experimental error is indicated by horizontal error bars and the 89% Bayesian credible interval is indicated by vertical error bars.


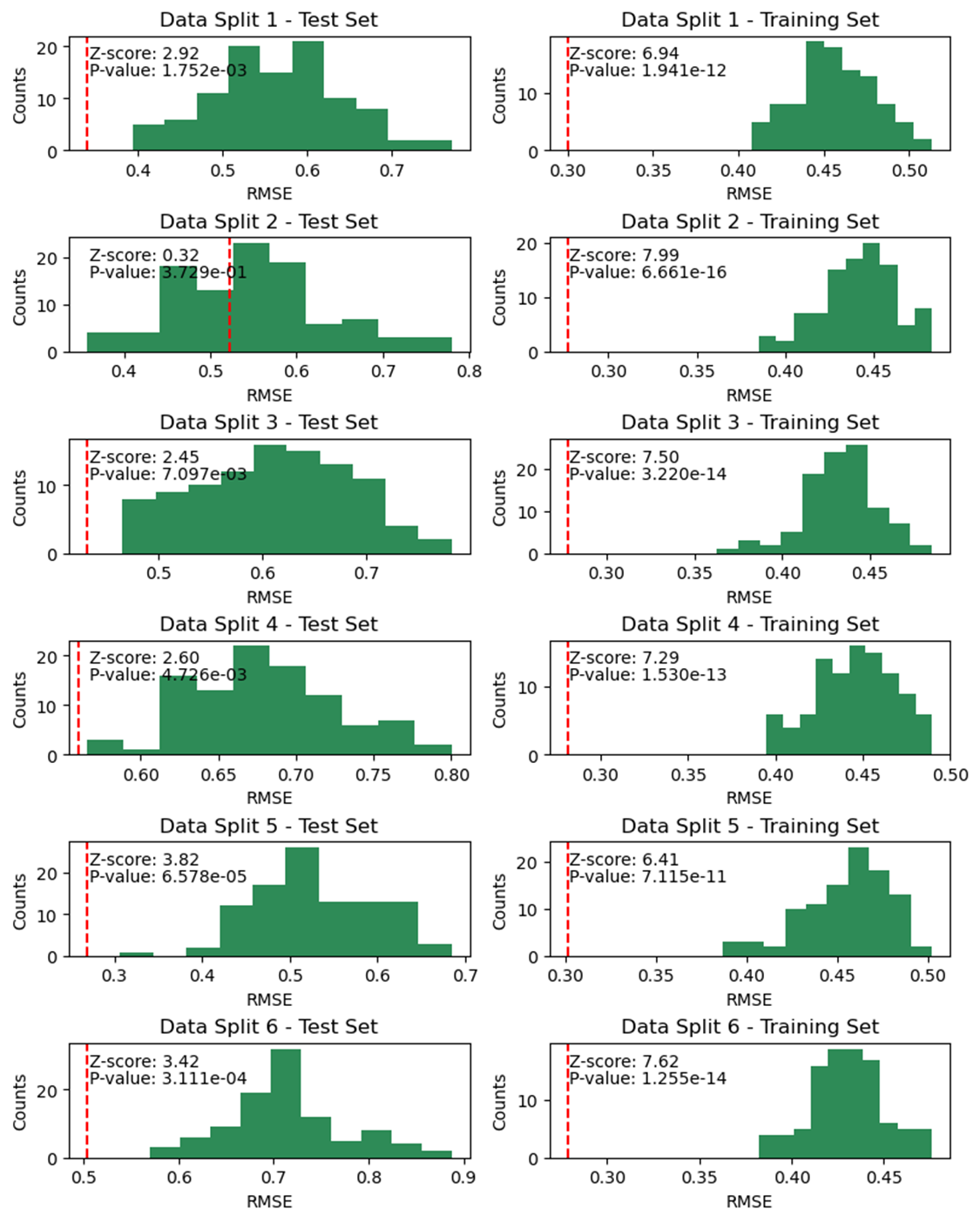


**Figure S8:** Histograms of the test set (left column) and training set (right column) RMSE scores resulting from the y randomization approach for each of six models produced for prediction of LogG′. The RMSE value for true data is shown as a vertical, red, dashed line.


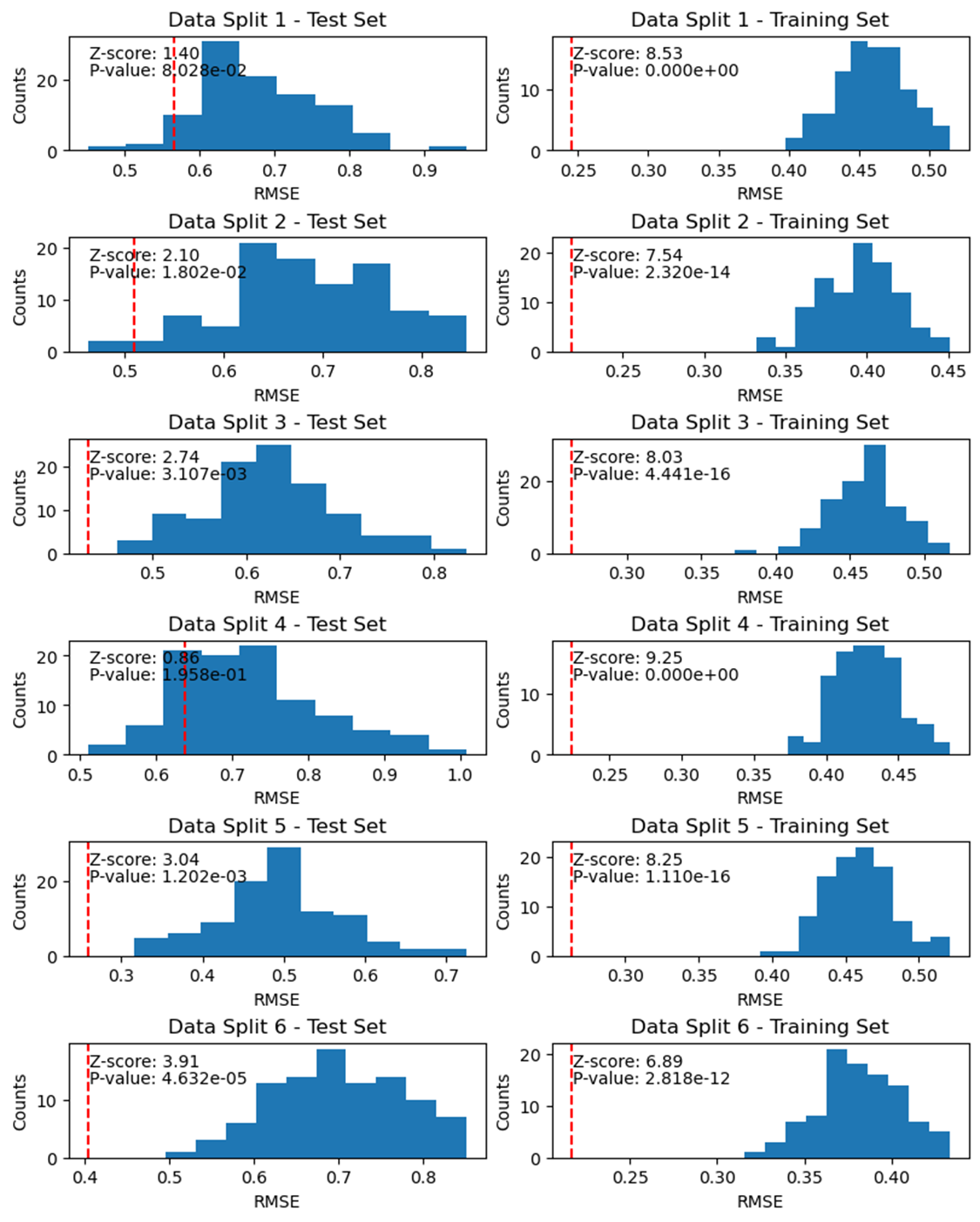


**Figure S9:** KDE plots of the test set (left column) and training set (right column) RMSE scores resulting from the y randomization approach for each of six models produced for prediction of LogG″. The RMSE value for true data is shown as a vertical, red, dashed line.


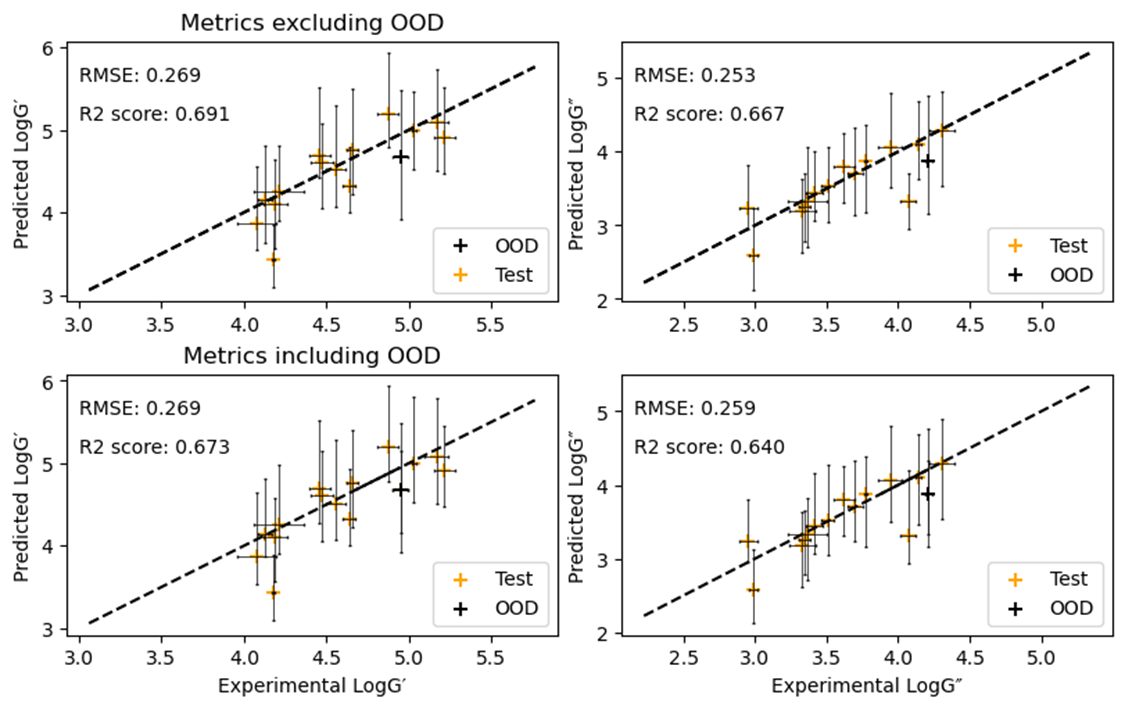


**Figure S10:** Comparison of model performance metrics excluding and including out of domain points in calculations. Scatterplots of measured testing set rheological values (x-axis) plotted against BART-predicted G″ (y-axis) for the best performing data split 5. The line y=x is plotted as a dashed black line for reference. Experimental error is indicated by horizontal error bars and the 89% Bayesian credible interval is indicated by vertical error bars.


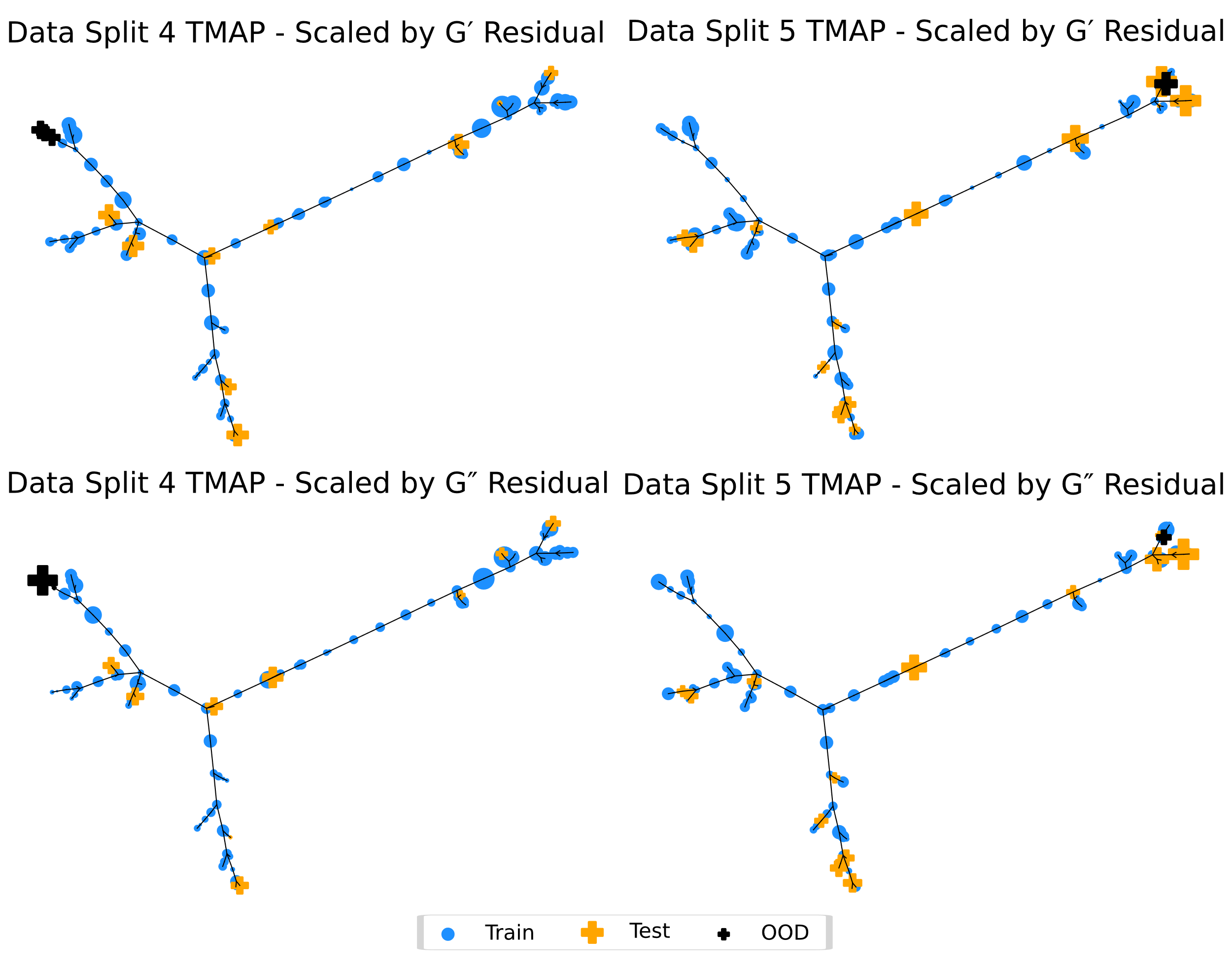


**Figure S11:** TMAP representations of the LMWG dataset with data points scaled by residuals for LogG′ and LogG″ predictions.


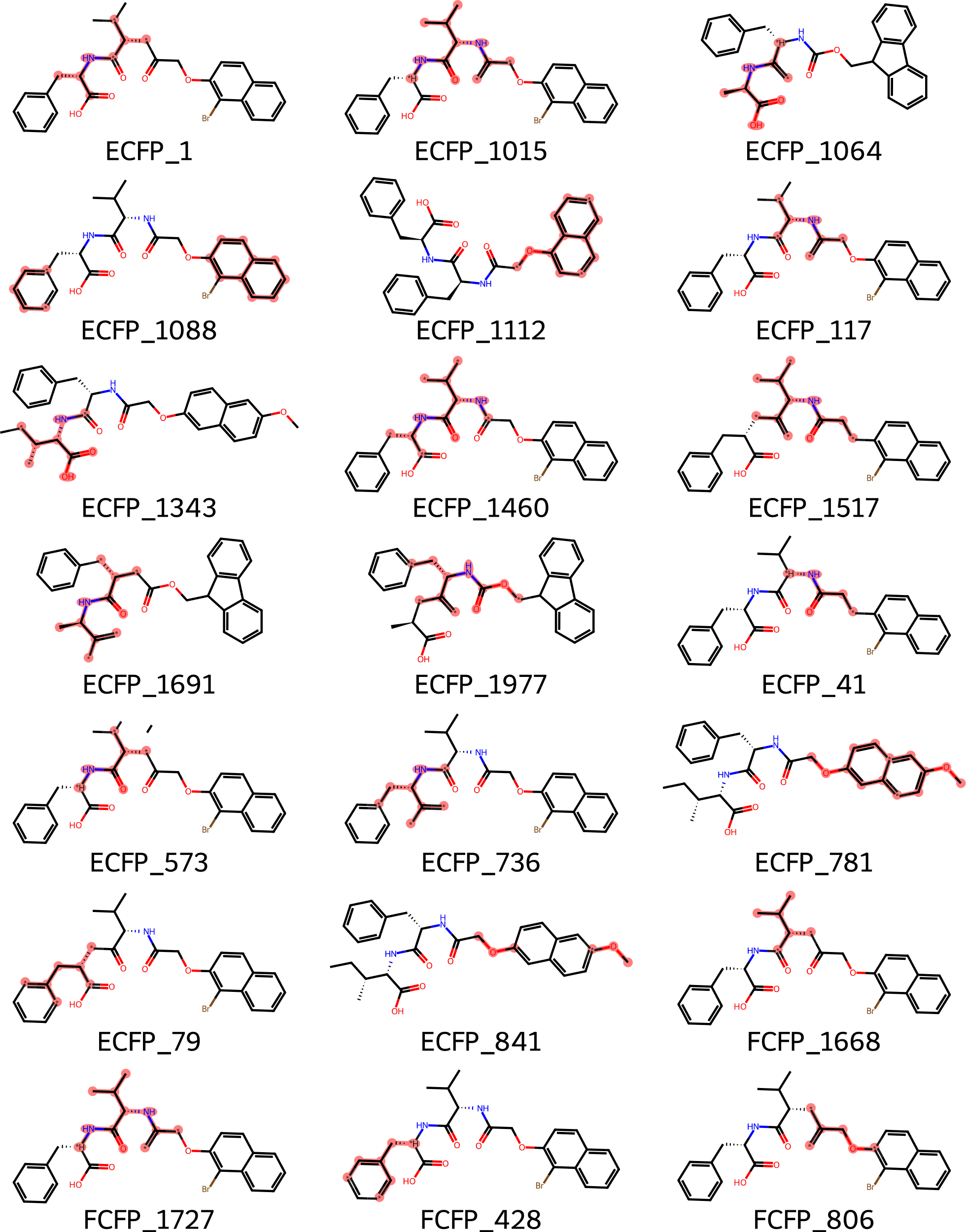


**Figure S12:** Structures of highly ranked fingerprints for validation and test set predictions in the G′ and G″. Fingerprint fragments are highlighted in red on a molecule containing the fingerprint. Connecting atoms are depicted as asterisks.

## Alternative Modelling Method Figures


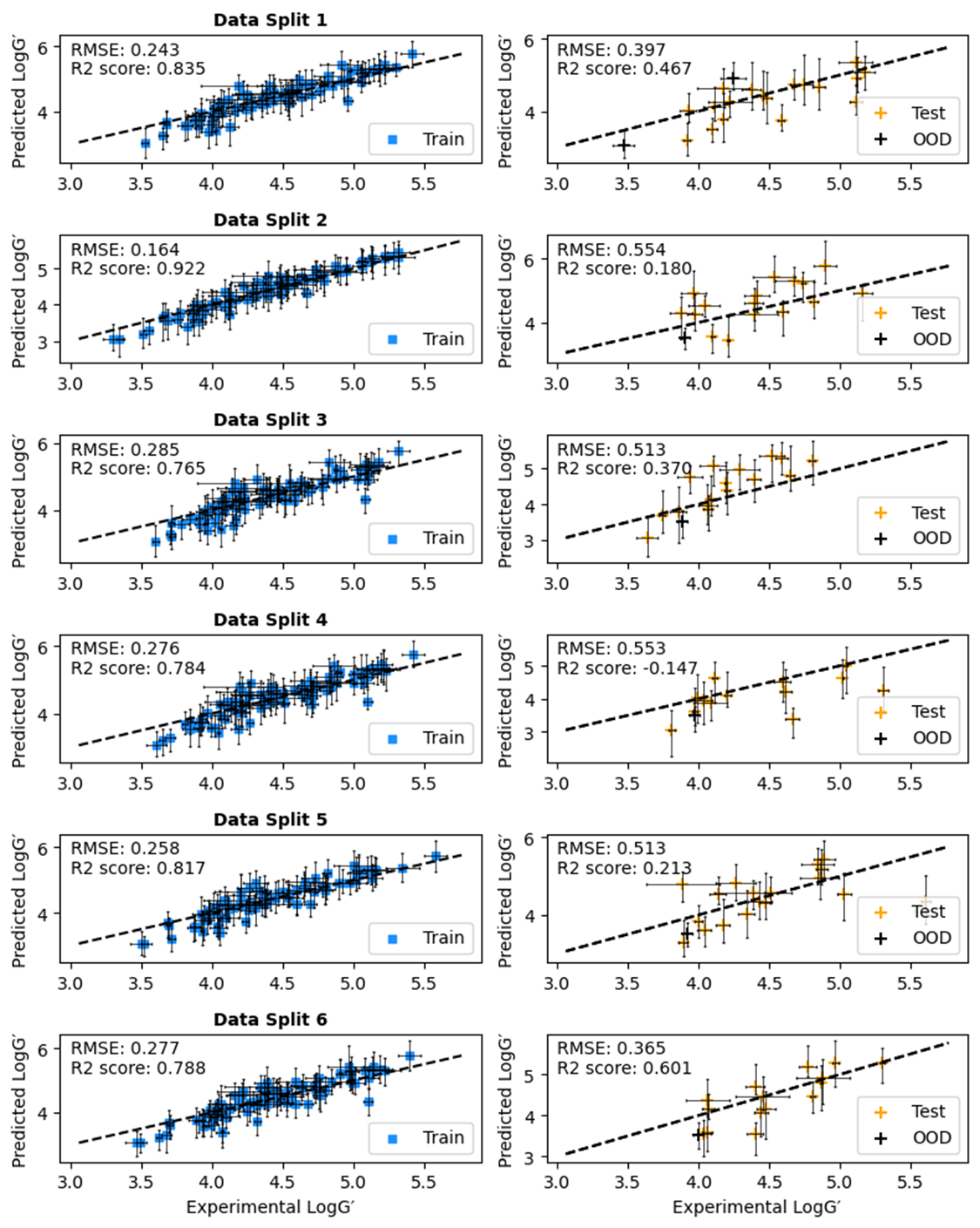


**Figure S13:** Prediction summaries for models produced using non-stratified dataset construction. Scatterplots of measured rheological values (x-axis) plotted against BART-predicted G′(y-axis) for each of the six models produced from unique data splits. The line y=x is plotted as a dashed black line for reference. Experimental error is indicated by horizontal error bars and the 89% Bayesian credible interval is indicated by vertical error bars.


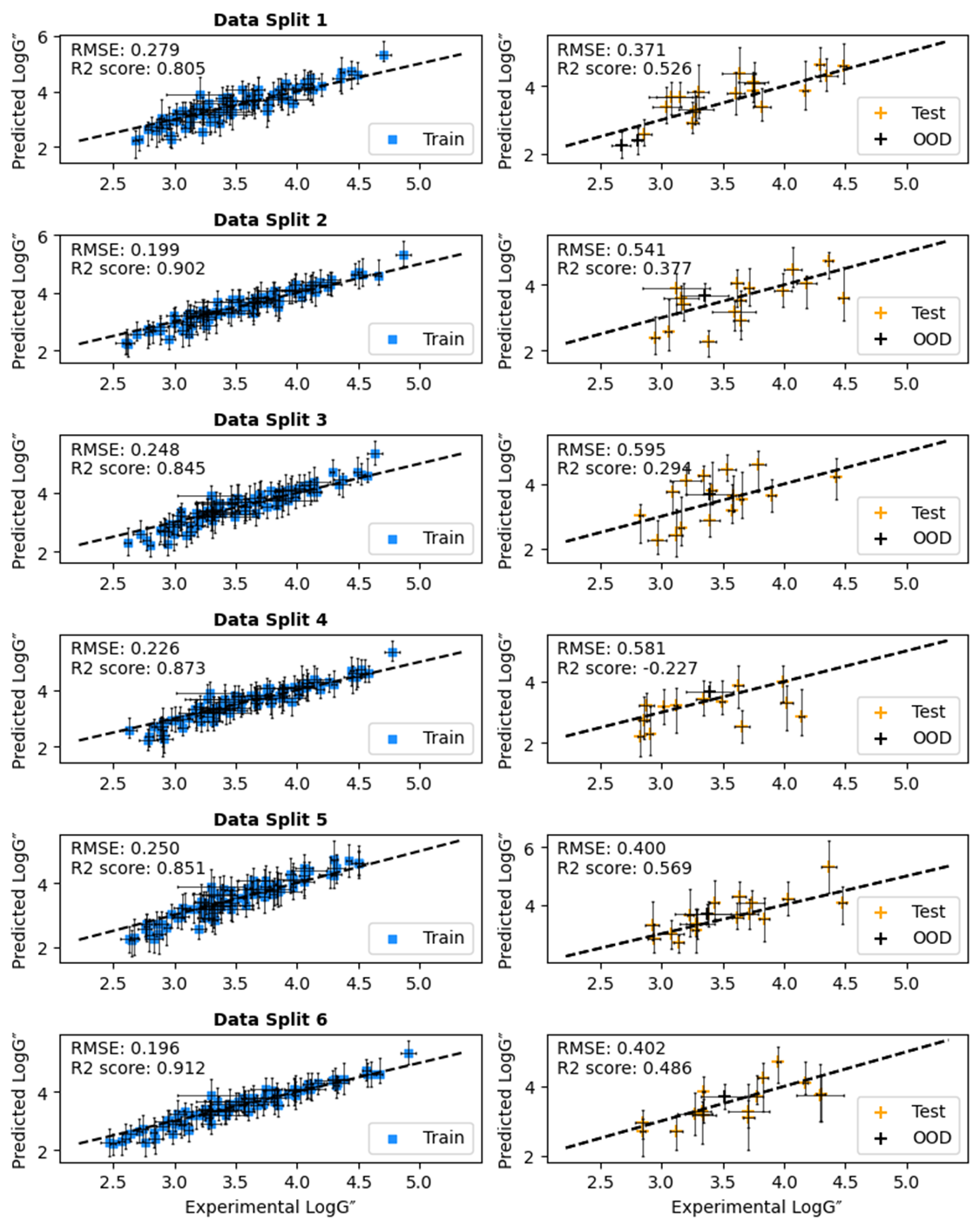


**Figure S14:** Prediction summaries for models produced using non-stratified dataset construction. Scatterplots of measured rheological values (x-axis) plotted against BART-predicted G″ (y-axis) for each of the six models produced from unique data splits. The line y=x is plotted as a dashed black line for reference. Experimental error is indicated by horizontal error bars and the 89% Bayesian credible interval is indicated by vertical error bars.


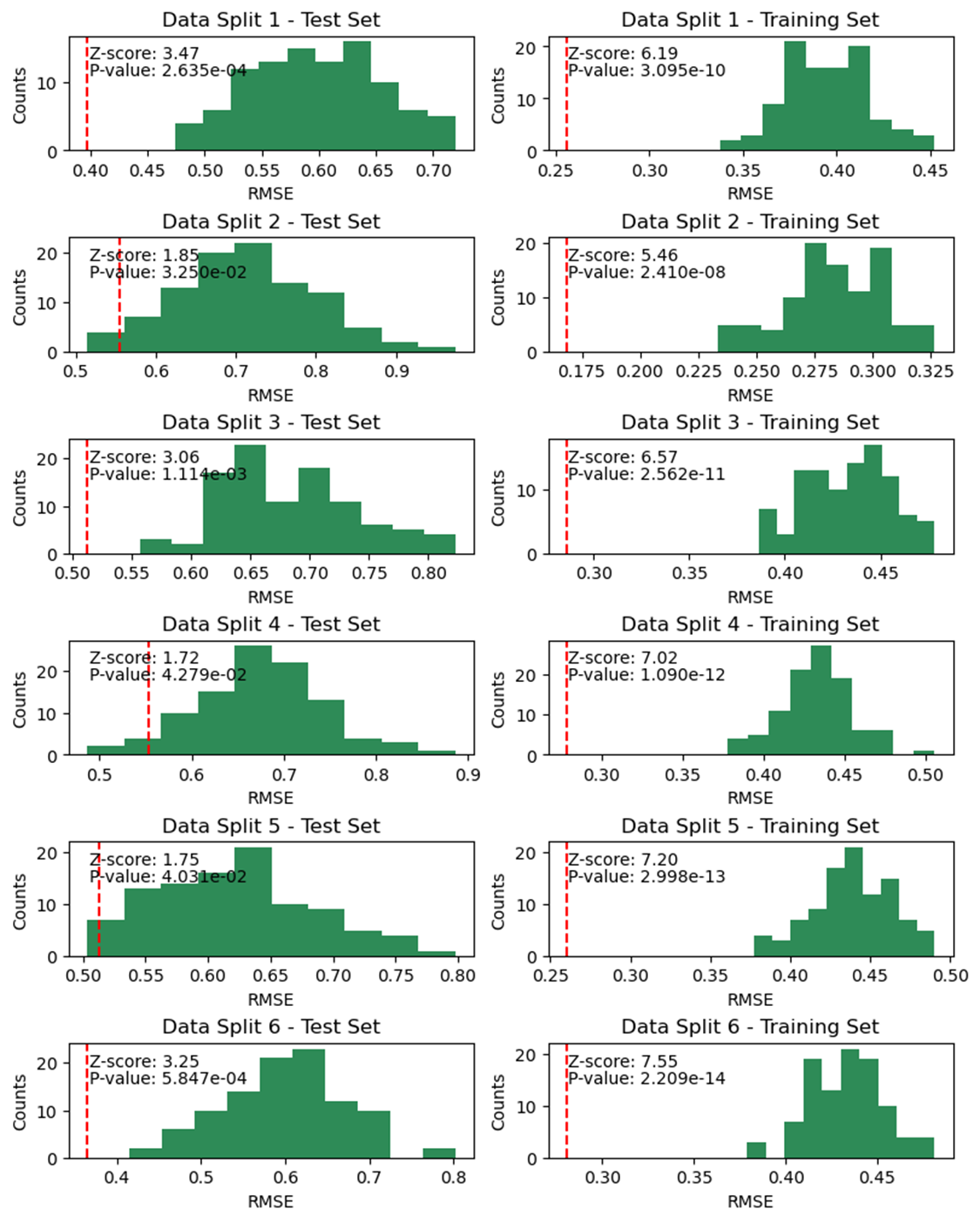


**Figure S15:** Summary of Y-randomization studies for models produced using non-stratified dataset construction. Histograms of the test set (left column) and training set (right column) RMSE scores resulting from the y randomization approach for each of six models produced for prediction of LogG′. The RMSE value for true data is shown as a vertical, red, dashed line.


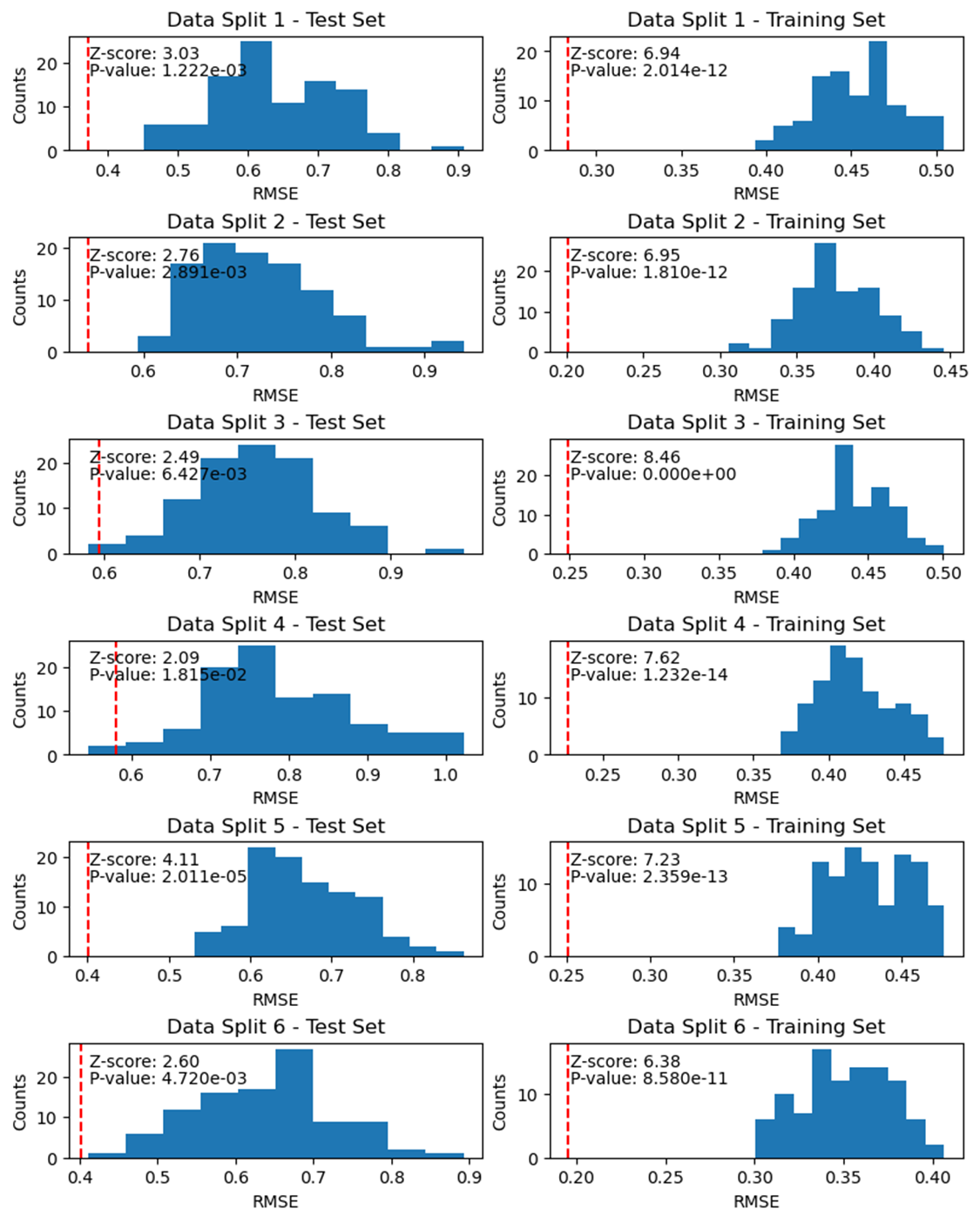


**Figure S16:** Summary of Y-randomization studies for models produced using non-stratified dataset construction. Histograms of the test set (left column) and training set (right column) RMSE scores resulting from the y randomization approach for each of six models produced for prediction of LogG″. The RMSE value for true data is shown as a vertical, red, dashed line.


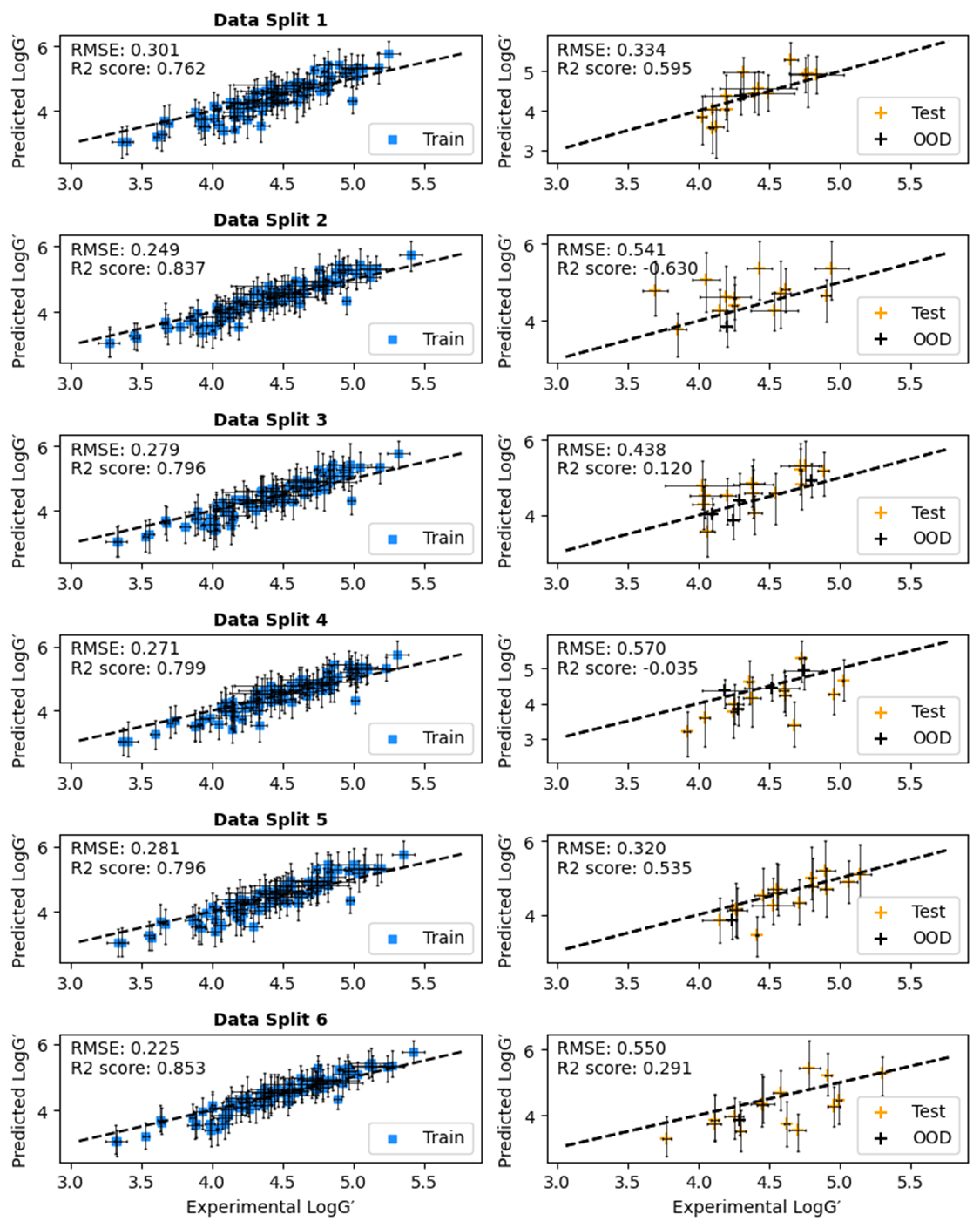


**Figure S17:** Prediction summaries for models produced using concentration as a descriptor. Scatterplots of measured rheological values (x-axis) plotted against BART-predicted G′(y-axis) for each of the six models produced from unique data splits. The line y=x is plotted as a dashed black line for reference. Experimental error is indicated by horizontal error bars and the 89% Bayesian credible interval is indicated by vertical error bars.


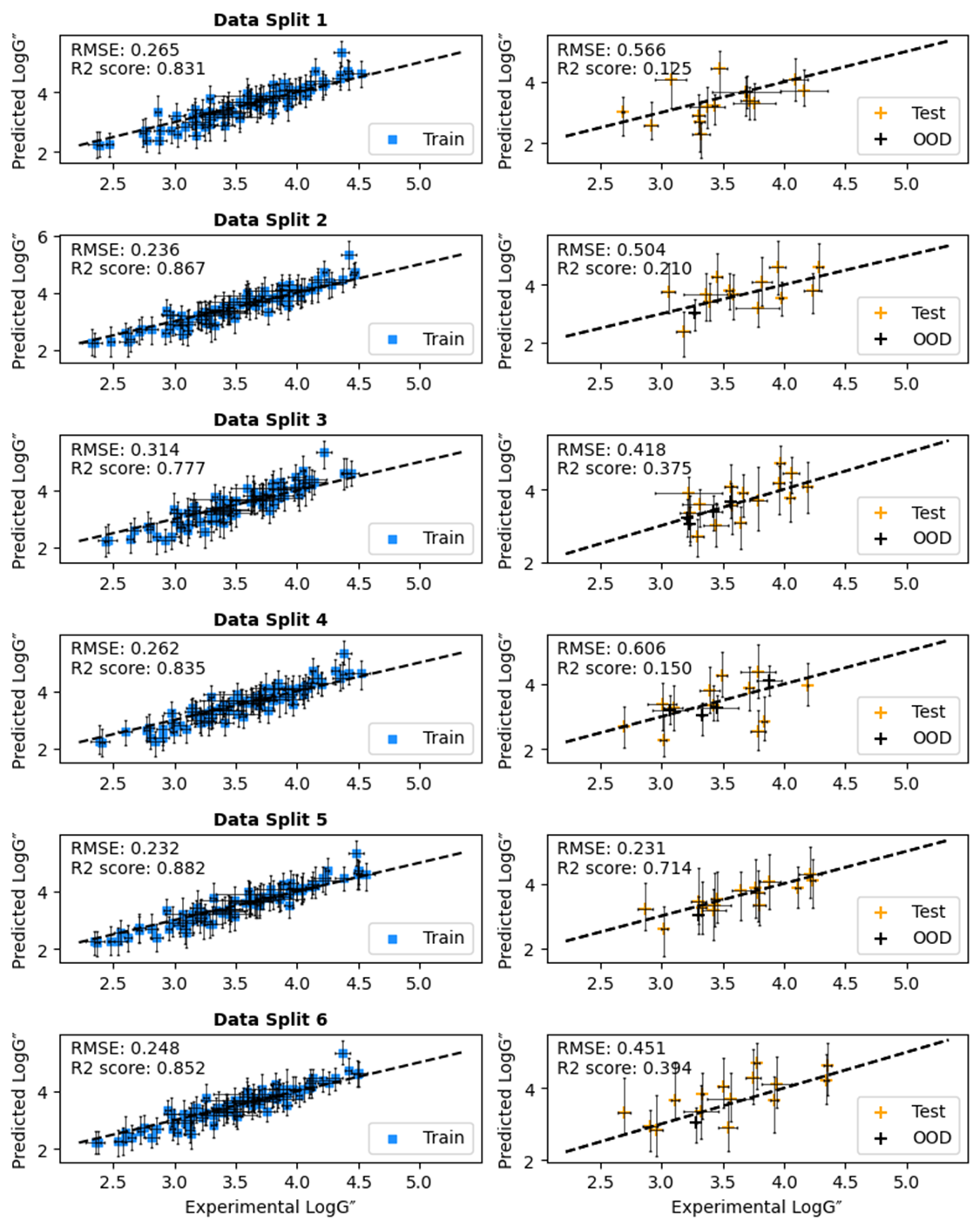


**Figure S18:** Prediction summaries for models produced using concentration as a descriptor. Scatterplots of measured rheological values (x-axis) plotted against BART-predicted G″ (y-axis) for each of the six models produced from unique data splits. The line y=x is plotted as a dashed black line for reference. Experimental error is indicated by horizontal error bars and the 89% Bayesian credible interval is indicated by vertical error bars.


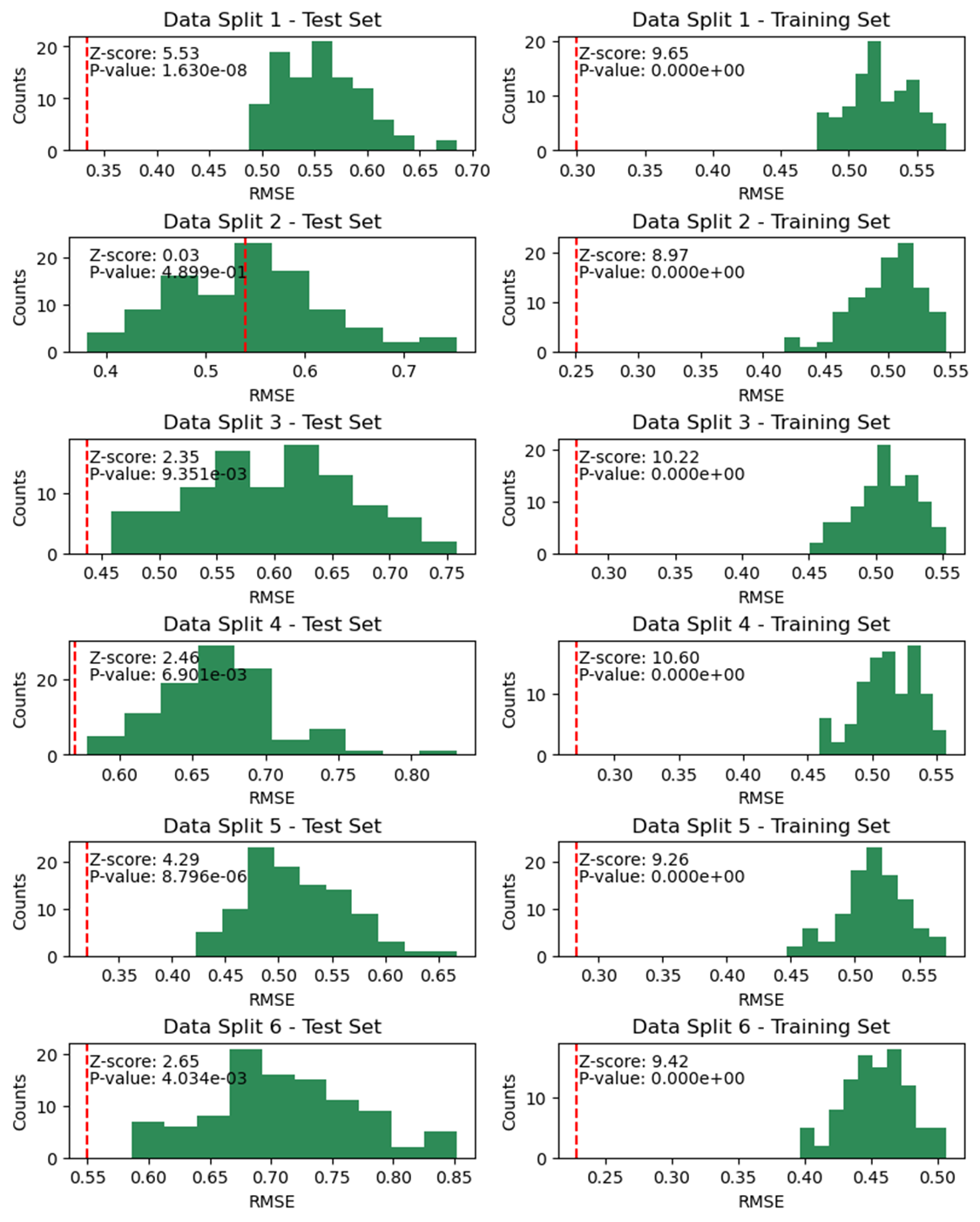


**Figure S19:** Summary of Y-randomization studies for models produced using concentration as a descriptor. Histograms of the test set (left column) and training set (right column) RMSE scores resulting from the y randomization approach for each of six models produced for prediction of LogG′. The RMSE value for true data is shown as a vertical, red, dashed line.


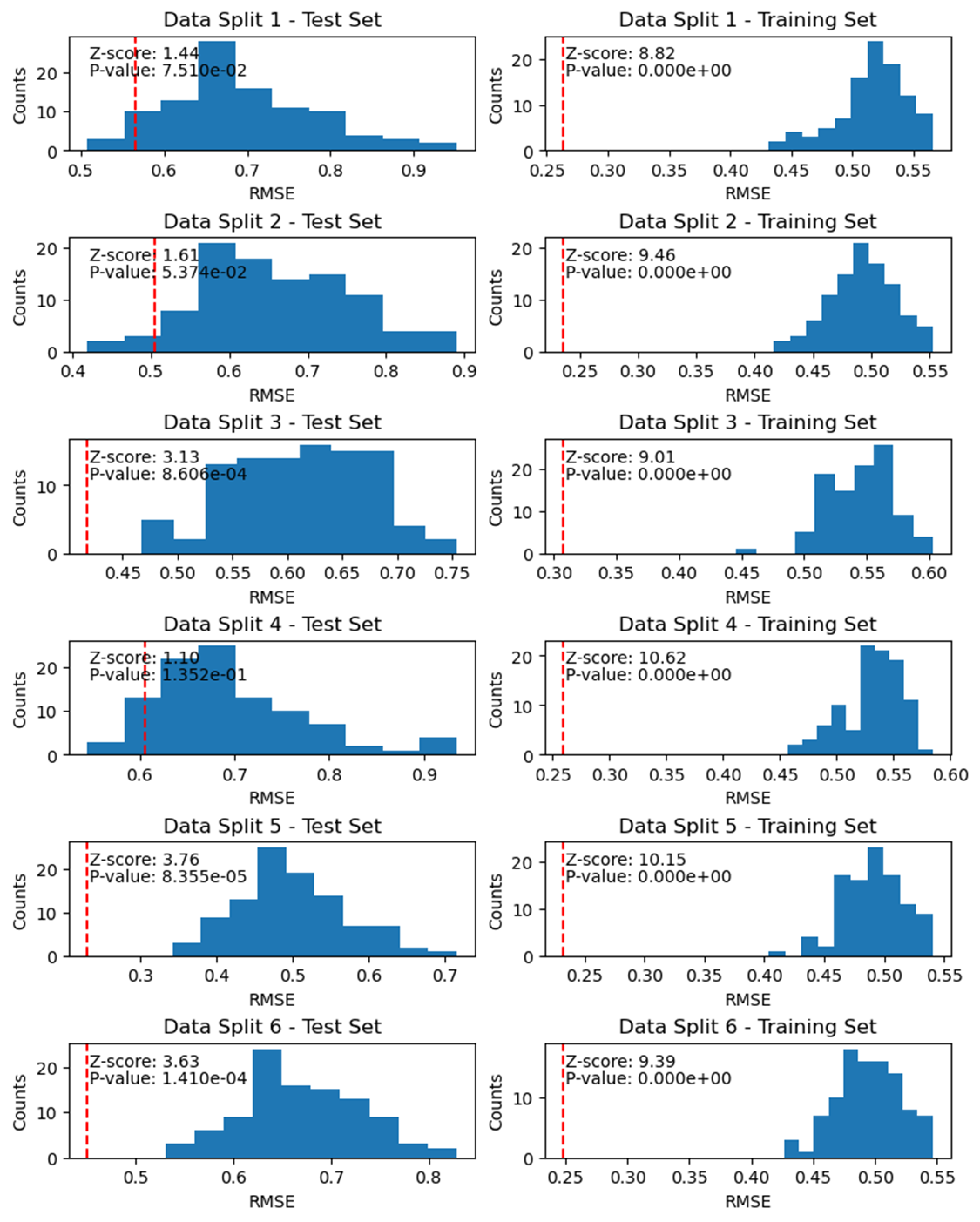


**Figure S20:** Summary of Y-randomization studies for models produced using concentration as a descriptor. Histograms of the test set (left column) and training set (right column) RMSE scores resulting from the y randomization approach for each of six models produced for prediction of LogG″. The RMSE value for true data is shown as a vertical, red, dashed line.


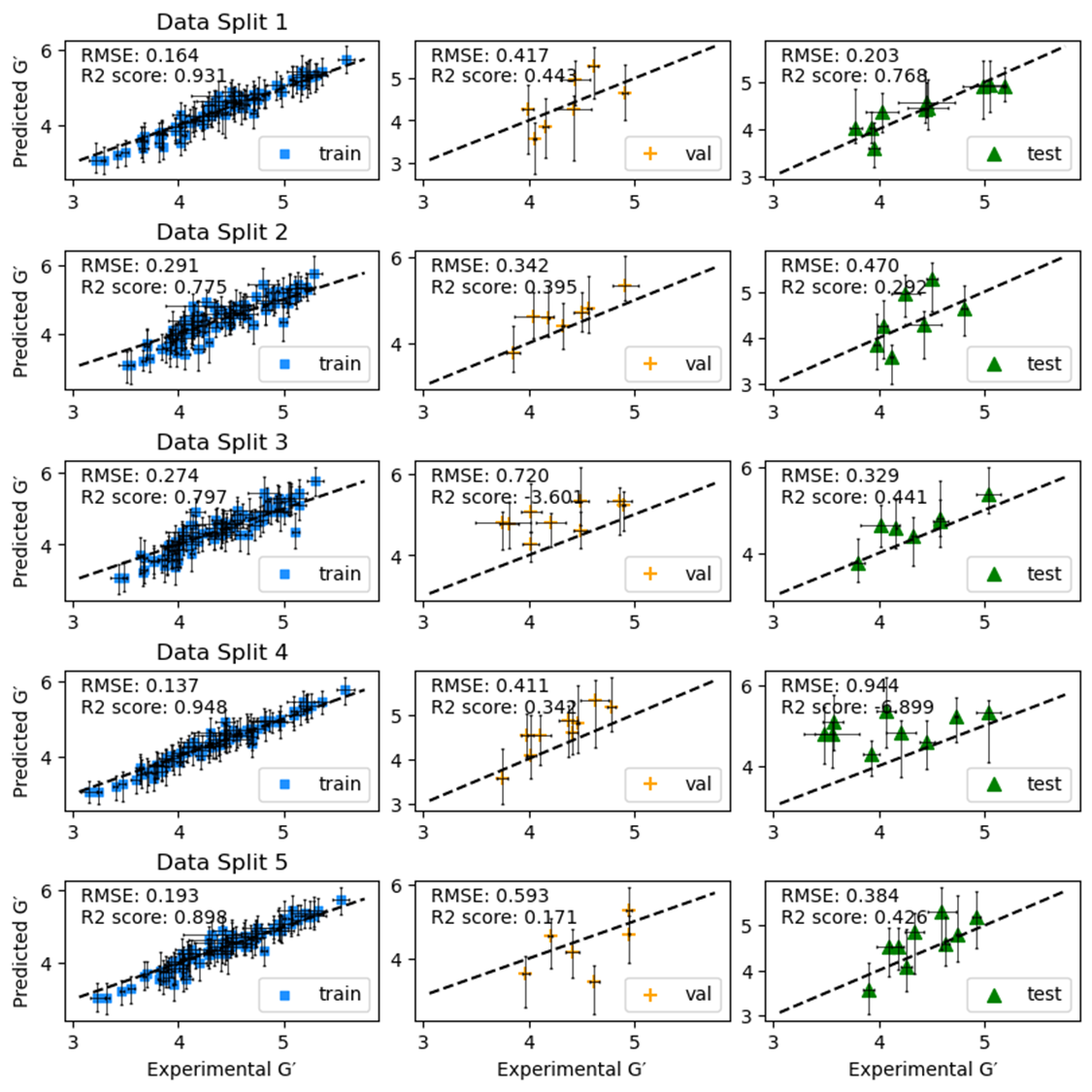


**Figure S21:** Prediction summaries for models produced using a separate validation set to tune hyperparameters – models 1-5. Scatterplots of measured rheological values (x-axis) plotted against BART-predicted G′(y-axis) for each of the six models produced from unique data splits. The line y=x is plotted as a dashed black line for reference. Experimental error is indicated by horizontal error bars and the 89% Bayesian credible interval is indicated by vertical error bars.


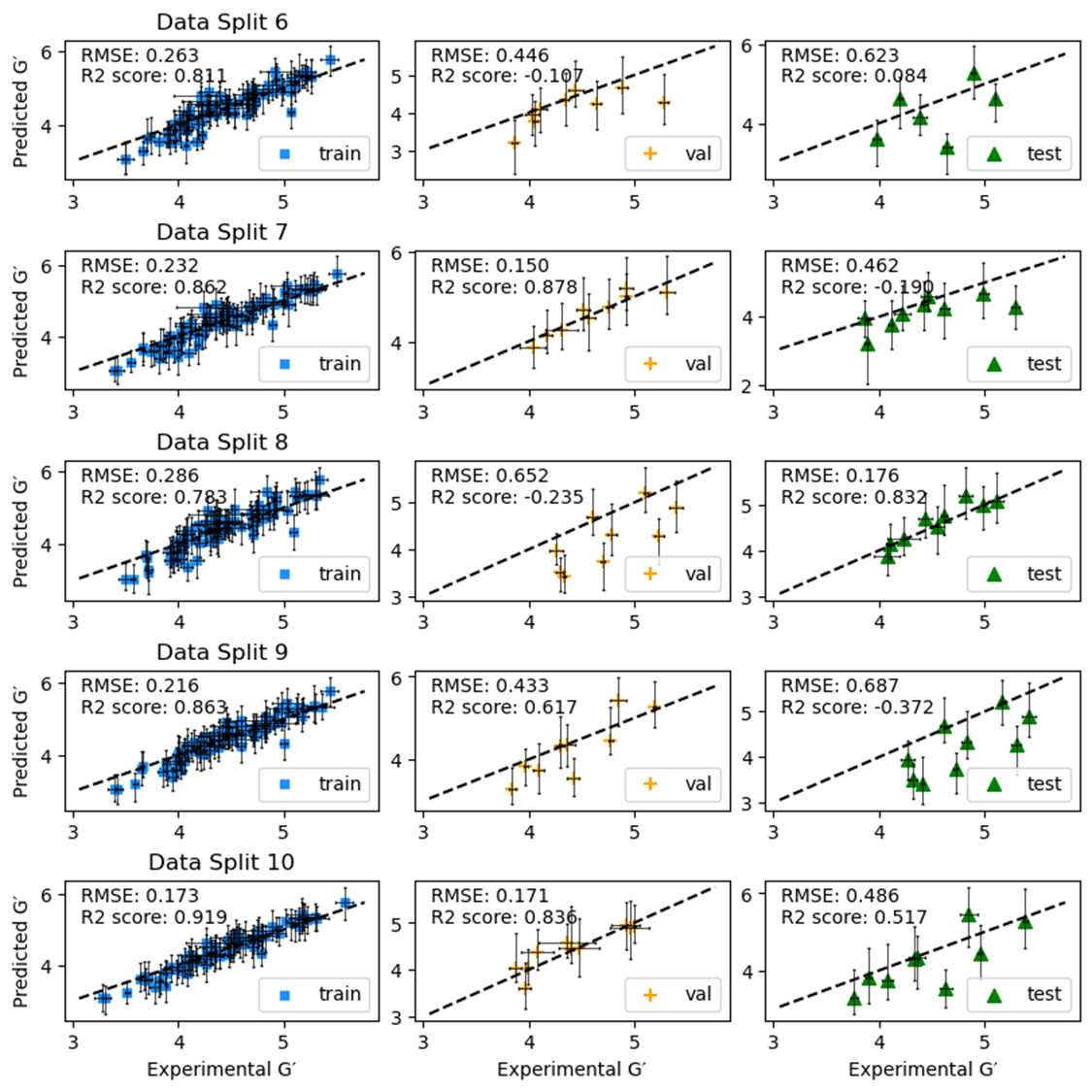


**Figure S22:** Prediction summaries for models produced using a separate validation set to tune hyperparameters – models 6-10. Scatterplots of measured rheological values (x-axis) plotted against BART-predicted G′(y-axis) for each of the six models produced from unique data splits. The line y=x is plotted as a dashed black line for reference. Experimental error is indicated by horizontal error bars and the 89% Bayesian credible interval is indicated by vertical error bars.


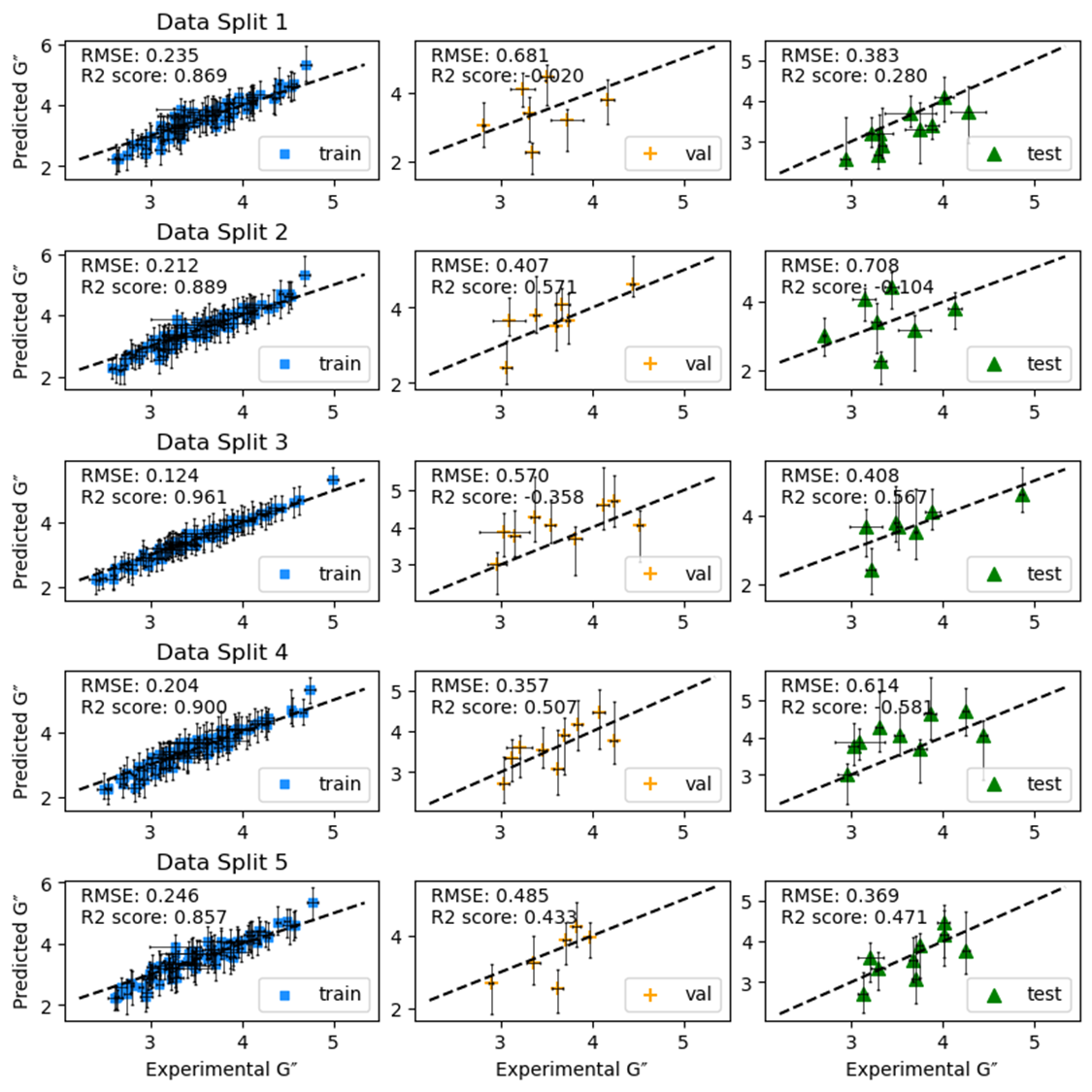


**Figure S23:** Prediction summaries for models produced using a separate validation set to tune hyperparameters – models 1-5. Scatterplots of measured rheological values (x-axis) plotted against BART-predicted G″ (y-axis) for each of the six models produced from unique data splits. The line y=x is plotted as a dashed black line for reference. Experimental error is indicated by horizontal error bars and the 89% Bayesian credible interval is indicated by vertical error bars.


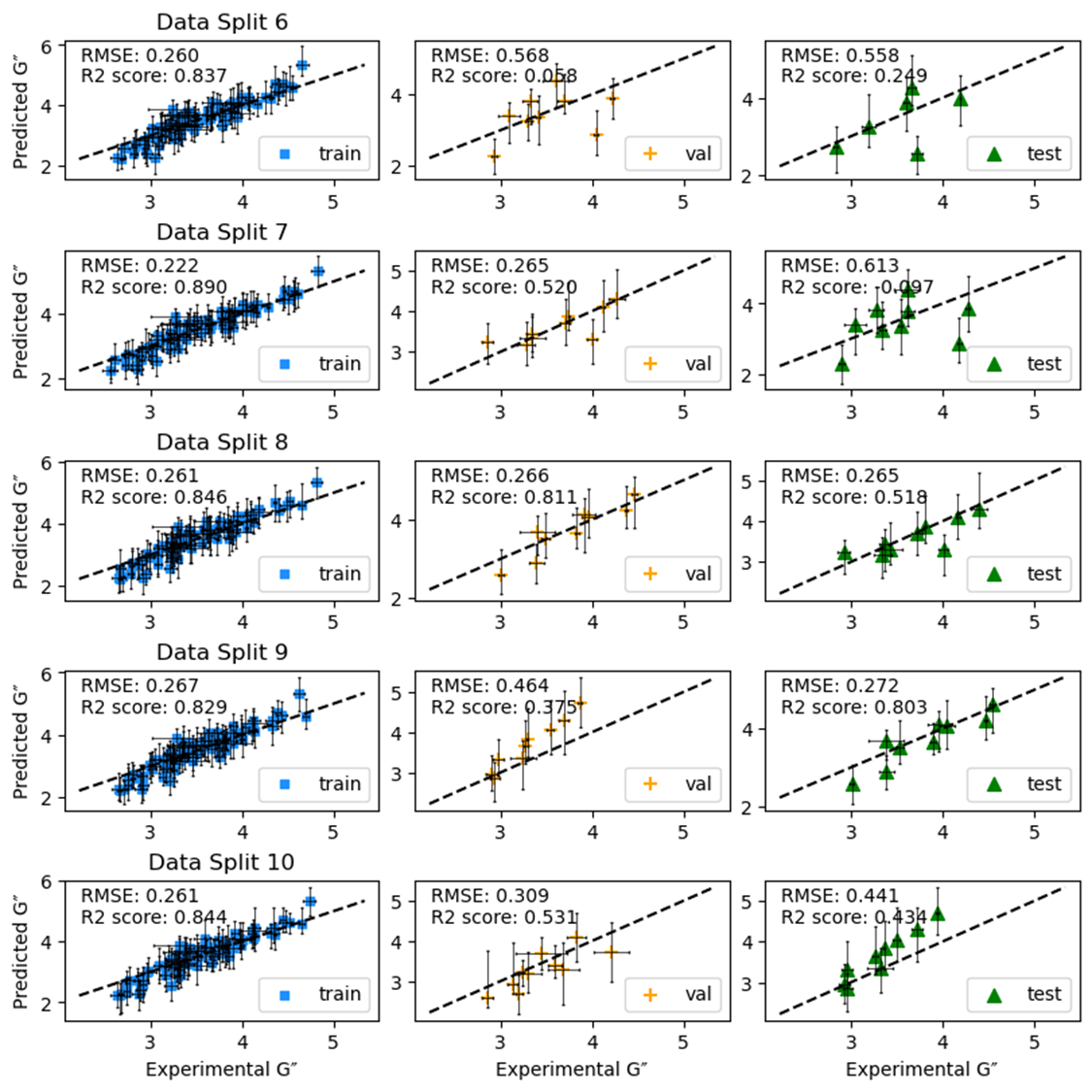


**Figure S24:** Prediction summaries for models produced using a separate validation set to tune hyperparameters – models 6-10. Scatterplots of measured rheological values (x-axis) plotted against BART-predicted G″ (y-axis) for each of the six models produced from unique data splits. The line y=x is plotted as a dashed black line for reference. Experimental error is indicated by horizontal error bars and the 89% Bayesian credible interval is indicated by vertical error bars.


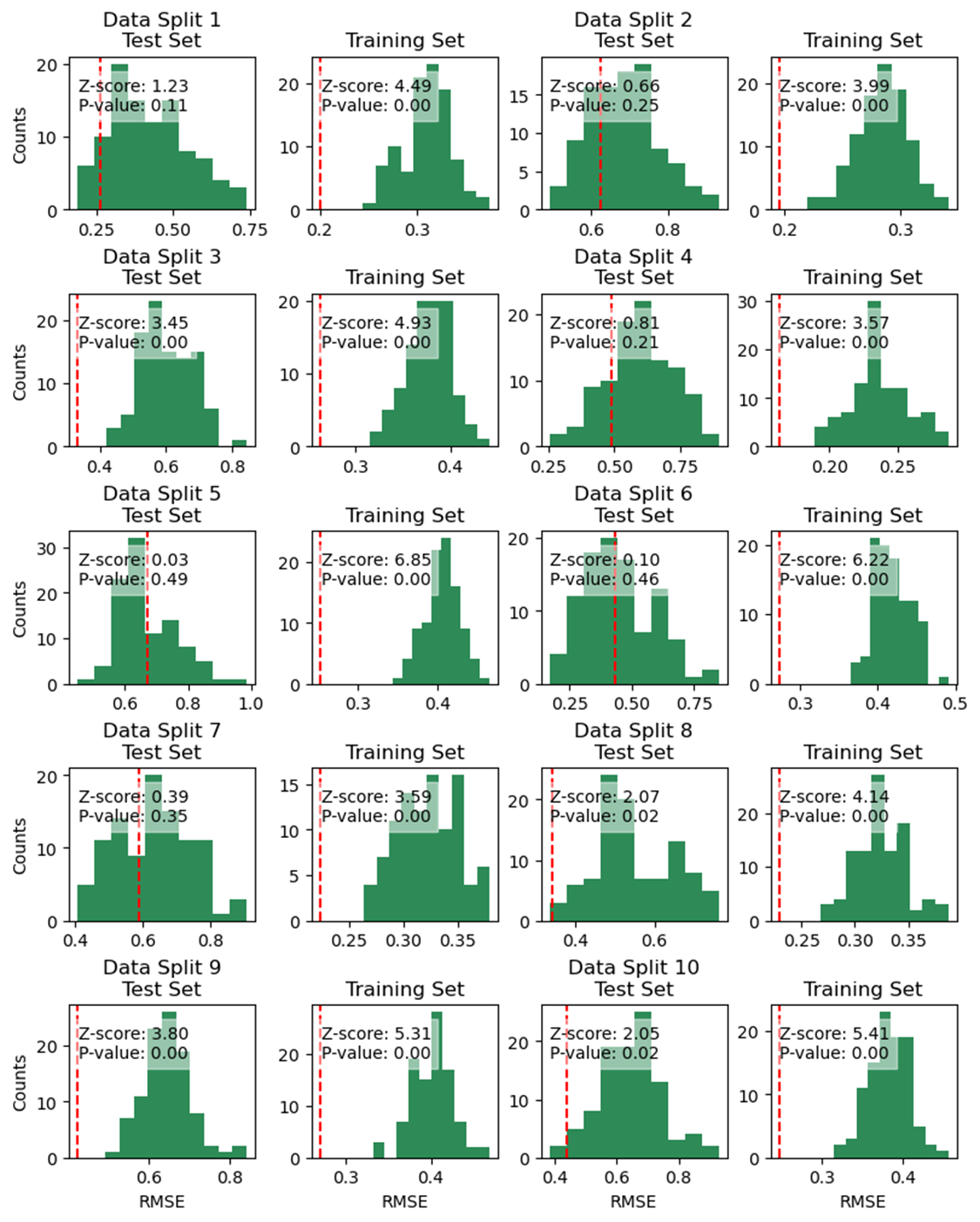


**Figure S25:** Summary of Y-randomization studies for models produced using a holdout validation set to tune hyperparameters. Histograms of the test set (left column) and training set (right column) RMSE scores resulting from the y randomization approach for each of six models produced for prediction of LogG′. The RMSE value for true data is shown as a vertical, red, dashed line.


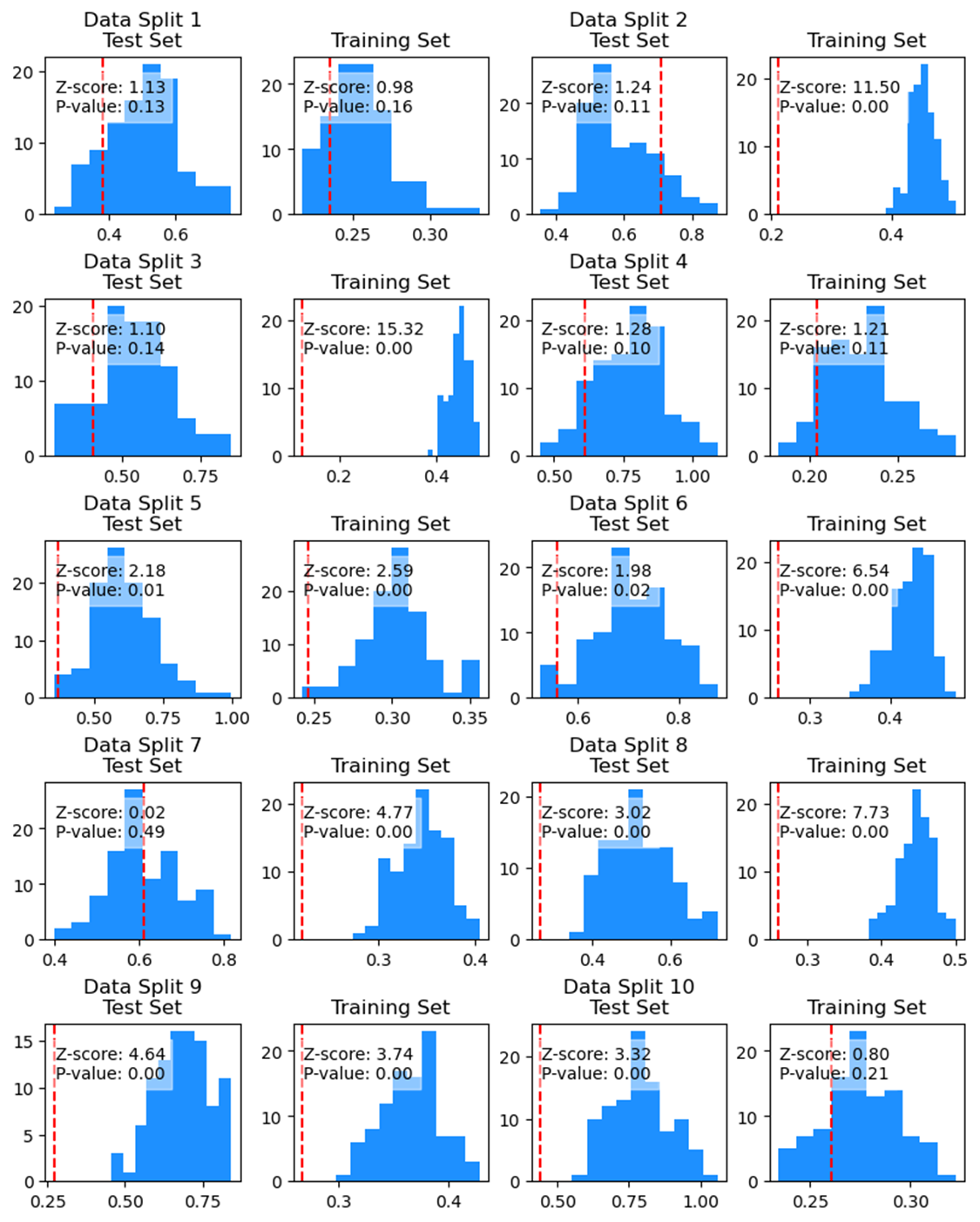


**Figure S26:** Summary of Y-randomization studies for models produced using a holdout validation set to tune hyperparameters. Histograms of the test set (left column) and training set (right column) RMSE scores resulting from the y randomization approach for each of six models produced for prediction of LogG″. The RMSE value for true data is shown as a vertical, red, dashed line.


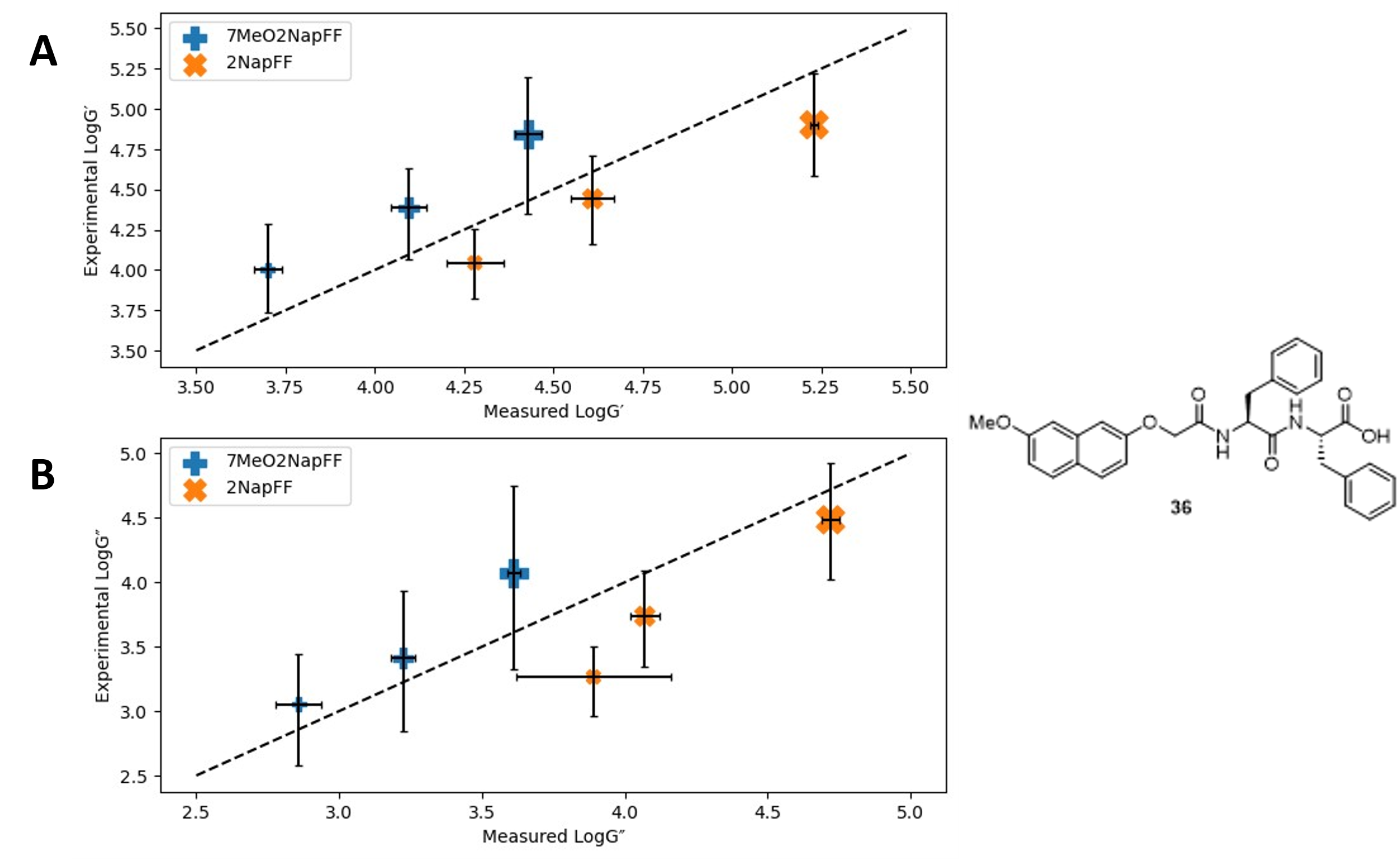


**Figure S27:** Scatterplots of measured rheological values (x-axis) plotted against BART-predicted G′ and G″ (**A** and **B**, respectively). The line y=x is plotted as a dashed black line for reference. Experimental error is indicated by horizontal error bars and the 89% Bayesian credible interval is indicated by vertical error bars. Points corresponding to predictions for 7MeONapFF are shown in blue and 2NapFF shown in orange. Points are scaled by concentration.

# Supplementary Tables

**Table S1:** Rheological data used to fit the BART models described in this work.

| **Cmpd** | **SMILES** | **G′±error** | **G″±error** | **Conc (mg/mL)** |
| --- | --- | --- | --- | --- |
| 1 | O=C(N[C@@H](CC1=CC=CC=C1)C(N[C@@H](CC2=CC=CC=C2)C(O)=O)=O)COC3=C(Br)C4=CC=CC=C4C=C3 | 3.96±0.07 | 2.89±0.08 | 2.5 |
| 1 | O=C(N[C@@H](CC1=CC=CC=C1)C(N[C@@H](CC2=CC=CC=C2)C(O)=O)=O)COC3=C(Br)C4=CC=CC=C4C=C3 | 4.69±0.05 | 3.67±0.03 | 5 |
| 1 | O=C(N[C@@H](CC1=CC=CC=C1)C(N[C@@H](CC2=CC=CC=C2)C(O)=O)=O)COC3=C(Br)C4=CC=CC=C4C=C3 | 5.21±0.02 | 4.23±0.02 | 10 |
| 2 | O=C(N[C@@H](CC1=CC=CC=C1)C(O)=O)[C@H](CC2=CC=CC=C2)NC(OCC3C4=CC=CC=C4C5=C3C=CC=C5)=O | 4.53±0.06 | 3.35±0.08 | 2.5 |
| 2 | O=C(N[C@@H](CC1=CC=CC=C1)C(O)=O)[C@H](CC2=CC=CC=C2)NC(OCC3C4=CC=CC=C4C5=C3C=CC=C5)=O | 4.58±0.04 | 3.54±0.04 | 5 |
| 2 | O=C(N[C@@H](CC1=CC=CC=C1)C(O)=O)[C@H](CC2=CC=CC=C2)NC(OCC3C4=CC=CC=C4C5=C3C=CC=C5)=O | 5.18±0.04 | 4.18±0.04 | 10 |
| 3 | O=C(N[C@@H](CC1=CC=CC=C1)C(N[C@@H](CC2=CC=CC=C2)C(O)=O)=O)COC3=CC4=CC=CC=C4C=C3 | 4.28±0.08 | 3.89±0.27 | 2.5 |
| 3 | O=C(N[C@@H](CC1=CC=CC=C1)C(N[C@@H](CC2=CC=CC=C2)C(O)=O)=O)COC3=CC4=CC=CC=C4C=C3 | 4.61±0.06 | 4.07±0.05 | 5 |
| 3 | O=C(N[C@@H](CC1=CC=CC=C1)C(N[C@@H](CC2=CC=CC=C2)C(O)=O)=O)COC3=CC4=CC=CC=C4C=C3 | 5.23±0.01 | 4.72±0.03 | 10 |
| 4 | O=C(NCC(O)=O)[C@H](CC(C)C)NC(OCC1C2=CC=CC=C2C3=C1C=CC=C3)=O | 4.78±0.09 | 3.77±0.05 | 2.5 |
| 4 | O=C(NCC(O)=O)[C@H](CC(C)C)NC(OCC1C2=CC=CC=C2C3=C1C=CC=C3)=O | 5.08±0.09 | 4.27±0.04 | 5 |
| 4 | O=C(NCC(O)=O)[C@H](CC(C)C)NC(OCC1C2=CC=CC=C2C3=C1C=CC=C3)=O | 5.35±0.09 | 4.62±0.07 | 10 |
| 5 | OC([C@H](CC1=CNC2=C1C=CC=C2)NC([C@H](CC3=CC=CC=C3)NC(OC(C)(C)C)=O)=O)=O | 4.15±0.04 | 3.19±0.10 | 2.5 |
| 5 | OC([C@H](CC1=CNC2=C1C=CC=C2)NC([C@H](CC3=CC=CC=C3)NC(OC(C)(C)C)=O)=O)=O | 4.69±0.07 | 3.71±0.05 | 5 |
| 5 | OC([C@H](CC1=CNC2=C1C=CC=C2)NC([C@H](CC3=CC=CC=C3)NC(OC(C)(C)C)=O)=O)=O | 5.20±0.06 | 4.11±0.02 | 10 |
| 6 | O=C(N[C@@H](CC1=CC=CC=C1)C(O)=O)C(C=C2)=CC=C2/C=C/C3=CC=C(C(N[C@@H](CC4=CC=CC=C4)C(O)=O)=O)C=C3 | 3.21±0.02 | 3.39±0.11 | 2.5 |
| 6 | O=C(N[C@@H](CC1=CC=CC=C1)C(O)=O)C(C=C2)=CC=C2/C=C/C3=CC=C(C(N[C@@H](CC4=CC=CC=C4)C(O)=O)=O)C=C3 | 3.77±0.02 | 3.82±0.09 | 5 |
| 6 | O=C(N[C@@H](CC1=CC=CC=C1)C(O)=O)C(C=C2)=CC=C2/C=C/C3=CC=C(C(N[C@@H](CC4=CC=CC=C4)C(O)=O)=O)C=C3 | 4.36±0.03 | 4.38±0.14 | 10 |
| 7 | O=C(N[C@@H](CC1=CC=CC=C1)C(N[C@@H](CC(C)C)C(O)=O)=O)COC2=CC=CC3=CC=CC=C32 | 4.54±0.11 | 3.60±0.14 | 2.5 |
| 7 | O=C(N[C@@H](CC1=CC=CC=C1)C(N[C@@H](CC(C)C)C(O)=O)=O)COC2=CC=CC3=CC=CC=C32 | 4.85±0.09 | 3.91±0.03 | 5 |
| 7 | O=C(N[C@@H](CC1=CC=CC=C1)C(N[C@@H](CC(C)C)C(O)=O)=O)COC2=CC=CC3=CC=CC=C32 | 5.31±0.14 | 4.46±0.07 | 10 |
| 8 | O=C(N[C@@H](CC1=CC=CC=C1)C(N[C@@H](C(C)C)C(O)=O)=O)COC2=C(Br)C3=CC=CC=C3C=C2 | 4.64±0.18 | 3.67±0.17 | 2.5 |
| 8 | O=C(N[C@@H](CC1=CC=CC=C1)C(N[C@@H](C(C)C)C(O)=O)=O)COC2=C(Br)C3=CC=CC=C3C=C2 | 4.73±0.07 | 4.09±0.08 | 5 |
| 8 | O=C(N[C@@H](CC1=CC=CC=C1)C(N[C@@H](C(C)C)C(O)=O)=O)COC2=C(Br)C3=CC=CC=C3C=C2 | 5.36±0.12 | 4.61±0.02 | 10 |
| 9 | O=C(N[C@@H](C(C)C)C(N[C@@H](C(C)C)C(N[C@@H](C(C)C)C(O)=O)=O)=O)COC1=CC2=CC=CC=C2C=C1 | 4.91±0.09 | 2.39±0.03 | 2.5 |
| 9 | O=C(N[C@@H](C(C)C)C(N[C@@H](C(C)C)C(N[C@@H](C(C)C)C(O)=O)=O)=O)COC1=CC2=CC=CC=C2C=C1 | 5.44±0.15 | 2.91±0.06 | 5 |
| 9 | O=C(N[C@@H](C(C)C)C(N[C@@H](C(C)C)C(N[C@@H](C(C)C)C(O)=O)=O)=O)COC1=CC2=CC=CC=C2C=C1 | 5.76±0.08 | 3.60±0.04 | 10 |
| 10 | OC([C@@H](NC(CN1C2=C(C3=C1C=CC=C3)C=CC=C2)=O)C)=O | 3.29±0.03 | 3.31±0.05 | 2.5 |
| 10 | OC([C@@H](NC(CN1C2=C(C3=C1C=CC=C3)C=CC=C2)=O)C)=O | 3.73±0.05 | 3.66±0.03 | 5 |
| 10 | OC([C@@H](NC(CN1C2=C(C3=C1C=CC=C3)C=CC=C2)=O)C)=O | 4.32±0.04 | 4.06±0.04 | 10 |
| 11 | O=C(COC1=CC2=CC=C(C=C2C=C1)OC)N[C@H](C(N[C@H](C(O)=O)[C@H](CC)C)=O)CC3=CC=CC=C3 | 3.86±0.12 | 3.23±0.06 | 2.5 |
| 11 | O=C(COC1=CC2=CC=C(C=C2C=C1)OC)N[C@H](C(N[C@H](C(O)=O)[C@H](CC)C)=O)CC3=CC=CC=C3 | 4.52±0.06 | 3.44±0.06 | 5 |
| 11 | O=C(COC1=CC2=CC=C(C=C2C=C1)OC)N[C@H](C(N[C@H](C(O)=O)[C@H](CC)C)=O)CC3=CC=CC=C3 | 4.99±0.01 | 3.32±0.05 | 10 |
| 12 | O=C(COC1=CC2=CC=C(Br)C=C2C=C1)N[C@H](C(N[C@H](C(N[C@H](C(O)=O)CC(C)C)=O)C(C)C)=O)C | 3.97±0.06 | 2.29±0.03 | 2.5 |
| 12 | O=C(COC1=CC2=CC=C(Br)C=C2C=C1)N[C@H](C(N[C@H](C(N[C@H](C(O)=O)CC(C)C)=O)C(C)C)=O)C | 4.27±0.04 | 2.86±0.02 | 10 |
| 12 | O=C(COC1=CC2=CC=C(Br)C=C2C=C1)N[C@H](C(N[C@H](C(N[C@H](C(O)=O)CC(C)C)=O)C(C)C)=O)C | 4.24±0.04 | 3.36±0.02 | 5 |
| 13 | O=C(N[C@@H](CC(C)C)C(N[C@@H](CC1=CC=CC=C1)C(N[C@@H](CC2=CC=CC=C2)C(O)=O)=O)=O)COC3=C(Br)C4=CC=CC=C4C=C3 | 3.52±0.03 | 3.69±0.19 | 2.5 |
| 13 | O=C(N[C@@H](CC(C)C)C(N[C@@H](CC1=CC=CC=C1)C(N[C@@H](CC2=CC=CC=C2)C(O)=O)=O)=O)COC3=C(Br)C4=CC=CC=C4C=C3 | 3.74±0.02 | 4.11±0.12 | 5 |
| 13 | O=C(N[C@@H](CC(C)C)C(N[C@@H](CC1=CC=CC=C1)C(N[C@@H](CC2=CC=CC=C2)C(O)=O)=O)=O)COC3=C(Br)C4=CC=CC=C4C=C3 | 4.27±0.04 | 4.63±0.03 | 10 |
| 14 | O=C(N[C@@H](CC1=CC=CC=C1)C(N[C@@H](CC2=CC=CC=C2)C(O)=O)=O)COC3=CC4=CC=C(OC)C=C4C=C3 | 3.60±0.05 | 2.68±0.04 | 2.5 |
| 14 | O=C(N[C@@H](CC1=CC=CC=C1)C(N[C@@H](CC2=CC=CC=C2)C(O)=O)=O)COC3=CC4=CC=C(OC)C=C4C=C3 | 4.57±0.27 | 3.69±0.28 | 5 |
| 14 | O=C(N[C@@H](CC1=CC=CC=C1)C(N[C@@H](CC2=CC=CC=C2)C(O)=O)=O)COC3=CC4=CC=C(OC)C=C4C=C3 | 4.95±0.13 | 4.10±0.10 | 10 |
| 15 | O=C(N[C@@H](C)C(N[C@@H](C(C)C)C(O)=O)=O)COC1=CC2=CC=C(Br)C=C2C=C1 | 3.85±0.03 | 3.04±0.01 | 2.5 |
| 15 | O=C(N[C@@H](C)C(N[C@@H](C(C)C)C(O)=O)=O)COC1=CC2=CC=C(Br)C=C2C=C1 | 4.97±0.14 | 4.09±0.13 | 5 |
| 15 | O=C(N[C@@H](C)C(N[C@@H](C(C)C)C(O)=O)=O)COC1=CC2=CC=C(Br)C=C2C=C1 | 5.29±0.05 | 4.46±0.07 | 10 |
| 16 | O=C(COC1=CC=C(C2=CC=CC=C21)Cl)N[C@H](C(N[C@H](C(O)=O)CC3=CC=CC=C3)=O)[C@H](CC)C | 3.60±0.04 | 2.72±0.02 | 2.5 |
| 16 | O=C(COC1=CC=C(C2=CC=CC=C21)Cl)N[C@H](C(N[C@H](C(O)=O)CC3=CC=CC=C3)=O)[C@H](CC)C | 4.16±0.06 | 3.26±0.07 | 5 |
| 16 | O=C(COC1=CC=C(C2=CC=CC=C21)Cl)N[C@H](C(N[C@H](C(O)=O)CC3=CC=CC=C3)=O)[C@H](CC)C | 5.29±0.02 | 4.26±0.04 | 10 |
| 17 | OC([C@@H](NC([C@@H](NC(COC1=CC=CC2=C1CCCC2)=O)CC3=CC=CC=C3)=O)CC4=CC=CC=C4)=O | 4.28±0.17 | 3.20±0.18 | 2.5 |
| 17 | OC([C@@H](NC([C@@H](NC(COC1=CC=CC2=C1CCCC2)=O)CC3=CC=CC=C3)=O)CC4=CC=CC=C4)=O | 4.27±0.05 | 3.41±0.06 | 5 |
| 17 | OC([C@@H](NC([C@@H](NC(COC1=CC=CC2=C1CCCC2)=O)CC3=CC=CC=C3)=O)CC4=CC=CC=C4)=O | 4.64±0.03 | 3.81±0.07 | 10 |
| 18 | CC1=CC=C(C2=C1C=CC=C2)C(N[C@H](C(N[C@H](C(N[C@H](C(O)=O)CC3=CC=CC=C3)=O)CC4=CC=CC=C4)=O)C)=O | 4.62±0.02 | 3.87±0.03 | 5 |
| 19 | CSCC[C@@H](C(N[C@H](C(N[C@H](C(O)=O)C)=O)CC1=CC=CC=C1)=O)NC(COC2=CC3=CC=C(C(C4=CC=CC=C4)=O)C=C3C=C2)=O | 4.39±0.02 | 3.67±0.02 | 5 |
| 20 | O=C(N[C@@H](C(C)C)C(NCC(O)=O)=O)COC1=CC2=CC=CC=C2C=C1 | 3.55±0.06 | 2.94±0.06 | 2.5 |
| 20 | O=C(N[C@@H](C(C)C)C(NCC(O)=O)=O)COC1=CC2=CC=CC=C2C=C1 | 4.46±0.02 | 3.84±0.01 | 5 |
| 20 | O=C(N[C@@H](C(C)C)C(NCC(O)=O)=O)COC1=CC2=CC=CC=C2C=C1 | 5.28±0.02 | 4.71±0.02 | 10 |
| 21 | O=C(COC1=CC=C(C=C1)C(F)(F)F)N[C@H](C(N[C@H](C(N[C@H](C(O)=O)CC2=CC=CC=C2)=O)[C@@H](C)CC)=O)CC3=CC=CC=C3 | 3.83±0.02 | 2.84±0.05 | 2.5 |
| 21 | O=C(COC1=CC=C(C=C1)C(F)(F)F)N[C@H](C(N[C@H](C(N[C@H](C(O)=O)CC2=CC=CC=C2)=O)[C@@H](C)CC)=O)CC3=CC=CC=C3 | 4.35±0.08 | 3.36±0.14 | 5 |
| 21 | O=C(COC1=CC=C(C=C1)C(F)(F)F)N[C@H](C(N[C@H](C(N[C@H](C(O)=O)CC2=CC=CC=C2)=O)[C@@H](C)CC)=O)CC3=CC=CC=C3 | 5.44±0.08 | 4.29±0.06 | 10 |
| 22 | O=C(N[C@@H](C)C(O)=O)COC1=CC2=CC=C(OC)C=C2C=C1 | 3.57±0.01 | 2.27±0.07 | 5 |
| 23 | O=C(N[C@@H](C(C)C)C(O)=O)[C@H](CC1=CC=CC=C1)NC(COC2=CC(C=CC=C3)=C3C=C2)=O | 4.02±0.06 | 3.13±0.03 | 2.5 |
| 23 | O=C(N[C@@H](C(C)C)C(O)=O)[C@H](CC1=CC=CC=C1)NC(COC2=CC(C=CC=C3)=C3C=C2)=O | 4.53±0.05 | 3.54±0.04 | 5 |
| 23 | O=C(N[C@@H](C(C)C)C(O)=O)[C@H](CC1=CC=CC=C1)NC(COC2=CC(C=CC=C3)=C3C=C2)=O | 4.34±0.03 | 5.34±0.06 | 10 |
| 24 | C[C@@H](C(NCC(O)=O)=O)NC(OCC1C(C=CC=C2)=C2C3=C1C=CC=C3)=O | 3.39±0.05 | 2.55±0.06 | 5 |
| 24 | C[C@@H](C(NCC(O)=O)=O)NC(OCC1C(C=CC=C2)=C2C3=C1C=CC=C3)=O | 4.64±0.00 | 3.98±0.00 | 10 |
| 25 | O=C(N[C@@H](CC1=CC=CC=C1)C(N[C@@H](CC2=CC=CC=C2)C(O)=O)=O)COC3=CC=CC4=CC=CC=C43 | 4.25±0.15 | 3.32±0.14 | 2.5 |
| 25 | O=C(N[C@@H](CC1=CC=CC=C1)C(N[C@@H](CC2=CC=CC=C2)C(O)=O)=O)COC3=CC=CC4=CC=CC=C43 | 4.75±0.03 | 3.87±0.01 | 5 |
| 25 | O=C(N[C@@H](CC1=CC=CC=C1)C(N[C@@H](CC2=CC=CC=C2)C(O)=O)=O)COC3=CC=CC4=CC=CC=C43 | 5.09±0.07 | 4.29±0.09 | 10 |
| 26 | O=C1OC2=CC=CC=C2C=C1OCC(N[C@@H](CC3=CC=CC=C3)C(N[C@@H](CC4=CC=CC=C4)C(O)=O)=O)=O | 4.02±0.02 | 3.21±0.02 | 2.5 |
| 27 | O=C(N[C@@H](C(C)C)C(NCC(O)=O)=O)COC1=C(Br)C2=CC=CC=C2C=C1 | 4.80±0.26 | 3.00±0.10 | 2.5 |
| 27 | O=C(N[C@@H](C(C)C)C(NCC(O)=O)=O)COC1=C(Br)C2=CC=CC=C2C=C1 | 4.82±0.14 | 3.68±0.07 | 5 |
| 27 | O=C(N[C@@H](C(C)C)C(NCC(O)=O)=O)COC1=C(Br)C2=CC=CC=C2C=C1 | 5.32±0.11 | 4.06±0.03 | 10 |
| 28 | O=C(COC1=CC2=CC=CC=C2C(Cl)=C1)N[C@H](C(N[C@H](C(N[C@H](C(O)=O)CC3=CC=CC=C3)=O)[C@H](CC)C)=O)CC(C)C | 3.56±0.05 | 2.71±0.05 | 2.5 |
| 28 | O=C(COC1=CC2=CC=CC=C2C(Cl)=C1)N[C@H](C(N[C@H](C(N[C@H](C(O)=O)CC3=CC=CC=C3)=O)[C@H](CC)C)=O)CC(C)C | 4.08±0.05 | 3.09±0.05 | 5 |
| 28 | O=C(COC1=CC2=CC=CC=C2C(Cl)=C1)N[C@H](C(N[C@H](C(N[C@H](C(O)=O)CC3=CC=CC=C3)=O)[C@H](CC)C)=O)CC(C)C | 4.81±0.01 | 3.77±0.03 | 10 |
| 29 | O=C(CN1C2=C(C3=C1C=CC=C3)C=CC=C2)N[C@H](C(O)=O)[C@@H](C)CC | 3.78±0.06 | 2.40±0.05 | 2.5 |
| 29 | O=C(CN1C2=C(C3=C1C=CC=C3)C=CC=C2)N[C@H](C(O)=O)[C@@H](C)CC | 4.58±0.01 | 3.80±0.02 | 5 |
| 29 | O=C(CN1C2=C(C3=C1C=CC=C3)C=CC=C2)N[C@H](C(O)=O)[C@@H](C)CC | 4.81±0.02 | 3.53±0.01 | 10 |
| 30 | O=C(N[C@@H](C(C)C)C(N[C@@H](CC1=CC=CC=C1)C(O)=O)=O)COC2=C(Br)C3=CC=CC=C3C=C2 | 3.43±0.01 | 2.59±0.03 | 2.5 |
| 30 | O=C(N[C@@H](C(C)C)C(N[C@@H](CC1=CC=CC=C1)C(O)=O)=O)COC2=C(Br)C3=CC=CC=C3C=C2 | 4.32±0.04 | 3.53±0.04 | 5 |
| 30 | O=C(N[C@@H](C(C)C)C(N[C@@H](CC1=CC=CC=C1)C(O)=O)=O)COC2=C(Br)C3=CC=CC=C3C=C2 | 4.90±0.06 | 4.06±0.08 | 10 |
| 31 | O=C(COC1=CC2=CC(OC)=CC=C2C=C1)N[C@H](C(N[C@H](C(O)=O)CC3=CC=CC=C3)=O)[C@@H](C)CC | 4.03±0.05 | 2.58±0.05 | 2.5 |
| 31 | O=C(COC1=CC2=CC(OC)=CC=C2C=C1)N[C@H](C(N[C@H](C(O)=O)CC3=CC=CC=C3)=O)[C@@H](C)CC | 4.44±0.02 | 2.92±0.05 | 5 |
| 31 | O=C(COC1=CC2=CC(OC)=CC=C2C=C1)N[C@H](C(N[C@H](C(O)=O)CC3=CC=CC=C3)=O)[C@@H](C)CC | 4.92±0.01 | 3.39±0.07 | 10 |
| 32 | O=C(N[C@@H](CC1=CC=CC=C1)C(N[C@@H](CC2=CC=CC=C2)C(O)=O)=O)COC3=CC4=CC=CC=C4C=C3OC | 4.37±0.15 | 3.20±0.14 | 2.5 |
| 32 | O=C(N[C@@H](CC1=CC=CC=C1)C(N[C@@H](CC2=CC=CC=C2)C(O)=O)=O)COC3=CC4=CC=CC=C4C=C3OC | 4.46±0.19 | 3.29±0.17 | 5 |
| 32 | O=C(N[C@@H](CC1=CC=CC=C1)C(N[C@@H](CC2=CC=CC=C2)C(O)=O)=O)COC3=CC4=CC=CC=C4C=C3OC | 4.90±0.19 | 3.73±0.20 | 10 |
| 33 | O=C([C@@H](NC(OCC1C2=C(C3=C1C=CC=C3)C=CC=C2)=O)CC4=CC=CC=C4)N[C@H](C(O)=O)C | 4.11±0.07 | 3.25±0.04 | 2.5 |
| 33 | O=C([C@@H](NC(OCC1C2=C(C3=C1C=CC=C3)C=CC=C2)=O)CC4=CC=CC=C4)N[C@H](C(O)=O)C | 4.61±0.07 | 3.80±0.07 | 5 |
| 33 | O=C([C@@H](NC(OCC1C2=C(C3=C1C=CC=C3)C=CC=C2)=O)CC4=CC=CC=C4)N[C@H](C(O)=O)C | 4.68±0.04 | 3.88±0.02 | 10 |
| 34 | OC(C1=CC=C(C=C1)C2OC[C@@H]3OC(OC[C@@H]3O2)C4=CC=C(C=C4)C(O)=O)=O | 3.06±0.02 | 2.22±0.02 | 2.5 |
| 34 | OC(C1=CC=C(C=C1)C2OC[C@@H]3OC(OC[C@@H]3O2)C4=CC=C(C=C4)C(O)=O)=O | 3.62±0.02 | 2.73±0.04 | 5 |
| 34 | OC(C1=CC=C(C=C1)C2OC[C@@H]3OC(OC[C@@H]3O2)C4=CC=C(C=C4)C(O)=O)=O | 4.09±0.02 | 3.21±0.01 | 10 |
| 35 | O=C(C1=CC=C(C=C1)C2OC[C@@H]3OC(O[C@H]([C@H](O)CO)[C@@H]3O2)C4=CC=C(C=C4)C(NCC(O)=O)=O)NCC(O)=O | 3.06±0.07 | 2.25±0.08 | 2.5 |
| 35 | O=C(C1=CC=C(C=C1)C2OC[C@@H]3OC(O[C@H]([C@H](O)CO)[C@@H]3O2)C4=CC=C(C=C4)C(NCC(O)=O)=O)NCC(O)=O | 3.70±0.02 | 2.64±0.02 | 5 |
| 35 | O=C(C1=CC=C(C=C1)C2OC[C@@H]3OC(O[C@H]([C@H](O)CO)[C@@H]3O2)C4=CC=C(C=C4)C(NCC(O)=O)=O)NCC(O)=O | 4.16±0.01 | 3.20±0.03 | 10 |

**Table S2:** Summary of the molecules assigned to training and testing sets for each of 6 splits.

| **Cmpd** | **SMILES** | **Data Split 1** | **Data Split 2** | **Data Split 3** | **Data Split 4** | **Data Split 5** | **Data Split 6** |
| --- | --- | --- | --- | --- | --- | --- | --- |
| **1** | O=C(N[C@@H](CC1=CC=CC=C1)C(N[C@@H](CC2=CC=CC=C2)C(O)=O)=O)COC3=C(Br)C4=CC=CC=C4C=C3 | Train | Train | Train | Train | Train | Test |
| **2** | O=C(N[C@@H](CC1=CC=CC=C1)C(O)=O)[C@H](CC2=CC=CC=C2)NC(OCC3C4=CC=CC=C4C5=C3C=CC=C5)=O | Train | Train | Test | Train | Train | Train |
| **3** | O=C(N[C@@H](CC1=CC=CC=C1)C(N[C@@H](CC2=CC=CC=C2)C(O)=O)=O)COC3=CC4=CC=CC=C4C=C3 | Train | Train | Test | Train | Train | Train |
| **4** | O=C(NCC(O)=O)[C@H](CC(C)C)NC(OCC1C2=CC=CC=C2C3=C1C=CC=C3)=O | Train | Test | Train | Train | Train | Train |
| **5** | OC([C@H](CC1=CNC2=C1C=CC=C2)NC([C@H](CC3=CC=CC=C3)NC(OC(C)(C)C)=O)=O)=O | Train | Train | Train | Train | Test | Train |
| **6** | O=C(N[C@@H](CC1=CC=CC=C1)C(O)=O)C(C=C2)=CC=C2/C=C/C3=CC=C(C(N[C@@H](CC4=CC=CC=C4)C(O)=O)=O)C=C3 | Train | Train | Train | Test | Train | Train |
| **7** | O=C(N[C@@H](CC1=CC=CC=C1)C(N[C@@H](CC(C)C)C(O)=O)=O)COC2=CC=CC3=CC=CC=C32 | Train | Train | Test | Train | Train | Train |
| **8** | O=C(N[C@@H](CC1=CC=CC=C1)C(N[C@@H](C(C)C)C(O)=O)=O)COC2=C(Br)C3=CC=CC=C3C=C2 | Train | Test | Train | Train | Train | Train |
| **9^a^** | O=C(N[C@@H](C(C)C)C(N[C@@H](C(C)C)C(N[C@@H](C(C)C)C(O)=O)=O)=O)COC1=CC2=CC=CC=C2C=C1 | Train | Train | Train | Train | Train | Train |
| **10** | OC([C@@H](NC(CN1C2=C(C3=C1C=CC=C3)C=CC=C2)=O)C)=O | Train | Train | Train | Train | Train | Test |
| **11** | O=C(COC1=CC2=CC=C(C=C2C=C1)OC)N[C@H](C(N[C@H](C(O)=O)[C@H](CC)C)=O)CC3=CC=CC=C3 | Train | Train | Train | Train | Test | Train |
| **12** | O=C(COC1=CC2=CC=C(Br)C=C2C=C1)N[C@H](C(N[C@H](C(N[C@H](C(O)=O)CC(C)C)=O)C(C)C)=O)C | Train | Train | Train | Test | Train | Train |
| **13** | O=C(N[C@@H](CC(C)C)C(N[C@@H](CC1=CC=CC=C1)C(N[C@@H](CC2=CC=CC=C2)C(O)=O)=O)=O)COC3=C(Br)C4=CC=CC=C4C=C3 | Train | Train | Train | Train | Train | Test |
| **14** | O=C(N[C@@H](CC1=CC=CC=C1)C(N[C@@H](CC2=CC=CC=C2)C(O)=O)=O)COC3=CC4=CC=C(OC)C=C4C=C3 | Test | Train | Train | Train | Train | Train |
| **15** | O=C(N[C@@H](C)C(N[C@@H](C(C)C)C(O)=O)=O)COC1=CC2=CC=C(Br)C=C2C=C1 | Test | Train | Train | Train | Train | Train |
| **16** | O=C(COC1=CC=C(C2=CC=CC=C21)Cl)N[C@H](C(N[C@H](C(O)=O)CC3=CC=CC=C3)=O)[C@H](CC)C | Train | Train | Train | Test | Train | Train |
| **17** | OC([C@@H](NC([C@@H](NC(COC1=CC=CC2=C1CCCC2)=O)CC3=CC=CC=C3)=O)CC4=CC=CC=C4)=O | Train | Test | Train | Train | Train | Train |
| **18** | CC1=CC=C(C2=C1C=CC=C2)C(N[C@H](C(N[C@H](C(N[C@H](C(O)=O)CC3=CC=CC=C3)=O)CC4=CC=CC=C4)=O)C)=O | Train | Train | Train | Test | Train | Train |
| **19** | CSCC[C@@H](C(N[C@H](C(N[C@H](C(O)=O)C)=O)CC1=CC=CC=C1)=O)NC(COC2=CC3=CC=C(C(C4=CC=CC=C4)=O)C=C3C=C2)=O | Train | Test | Train | Train | Train | Train |
| **20** | O=C(N[C@@H](C(C)C)C(NCC(O)=O)=O)COC1=CC2=CC=CC=C2C=C1 | Train | Train | Train | Train | Train | Test |
| **21** | O=C(COC1=CC=C(C=C1)C(F)(F)F)N[C@H](C(N[C@H](C(N[C@H](C(O)=O)CC2=CC=CC=C2)=O)[C@@H](C)CC)=O)CC3=CC=CC=C3 | Train | Train | Train | Train | Train | Test |
| **22** | O=C(N[C@@H](C)C(O)=O)COC1=CC2=CC=C(OC)C=C2C=C1 | Test | Train | Train | Train | Train | Train |
| **23^a^** | O=C(N[C@@H](C(C)C)C(O)=O)[C@H](CC1=CC=CC=C1)NC(COC2=CC(C=CC=C3)=C3C=C2)=O | Train | Train | Train | Train | Train | Train |
| **24** | C[C@@H](C(NCC(O)=O)=O)NC(OCC1C(C=CC=C2)=C2C3=C1C=CC=C3)=O | Train | Train | Train | Test | Train | Train |
| **25** | O=C(N[C@@H](CC1=CC=CC=C1)C(N[C@@H](CC2=CC=CC=C2)C(O)=O)=O)COC3=CC=CC4=CC=CC=C43 | Train | Train | Train | Train | Test | Train |
| **26** | O=C1OC2=CC=CC=C2C=C1OCC(N[C@@H](CC3=CC=CC=C3)C(N[C@@H](CC4=CC=CC=C4)C(O)=O)=O)=O | Test | Train | Train | Train | Train | Train |
| **27** | O=C(N[C@@H](C(C)C)C(NCC(O)=O)=O)COC1=C(Br)C2=CC=CC=C2C=C1 | Train | Train | Test | Train | Train | Train |
| **28** | O=C(COC1=CC2=CC=CC=C2C(Cl)=C1)N[C@H](C(N[C@H](C(N[C@H](C(O)=O)CC3=CC=CC=C3)=O)[C@H](CC)C)=O)CC(C)C | Train | Train | Test | Train | Train | Train |
| **29** | O=C(CN1C2=C(C3=C1C=CC=C3)C=CC=C2)N[C@H](C(O)=O)[C@@H](C)CC | Train | Test | Train | Train | Train | Train |
| **30** | O=C(N[C@@H](C(C)C)C(N[C@@H](CC1=CC=CC=C1)C(O)=O)=O)COC2=C(Br)C3=CC=CC=C3C=C2 | Train | Train | Train | Train | Test | Train |
| **31** | O=C(COC1=CC2=CC(OC)=CC=C2C=C1)N[C@H](C(N[C@H](C(O)=O)CC3=CC=CC=C3)=O)[C@@H](C)CC | Test | Train | Train | Train | Train | Train |
| **32** | O=C(N[C@@H](CC1=CC=CC=C1)C(N[C@@H](CC2=CC=CC=C2)C(O)=O)=O)COC3=CC4=CC=CC=C4C=C3OC | Test | Train | Train | Train | Train | Train |
| **33** | O=C([C@@H](NC(OCC1C2=C(C3=C1C=CC=C3)C=CC=C2)=O)CC4=CC=CC=C4)N[C@H](C(O)=O)C | Train | Train | Train | Train | Test | Train |
| **34^a^** | OC(C1=CC=C(C=C1)C2OC[C@@H]3OC(OC[C@@H]3O2)C4=CC=C(C=C4)C(O)=O)=O | Train | Train | Train | Train | Train | Train |
| **35^a^** | O=C(NCC(O)=O)C1=CC=C(C2OC[C@@H]3OC(C4=CC=C(C(NCC(O)=O)=O)C=C4)O[C@@H]([C@@H]3O2)[C@@H](CO)O)C=C1 | Train | Train | Train | Train | Train | Train |

**^a^** Molecules placed into training set due to having highest or lowest G′/G″

**Table S3:** Summary of metrics for models built using data split 5 with different values of hyperparameters number of trees (m) and probability of a node being terminal (alpha). Mean 5xCV RMSE metrics are given for the CV training folds and CV validation folds.

|  |  | **G′ RMSE** | | **G″ RMSE** | |
| --- | --- | --- | --- | --- | --- |
| **m^a^** | **alpha^b^** | **CV Training^c^** | **CV Validation^d^** | **CV Training^c^** | **CV Validation^d^** |
| 10 | 0.1 | 0.356 | 0.492 | 0.353 | 0.553 |
| 10 | 0.2 | 0.347 | 0.489 | 0.343 | 0.552 |
| 10 | 0.3 | 0.337 | 0.488 | 0.334 | 0.549 |
| 10 | 0.4 | 0.326 | 0.495 | 0.322 | 0.549 |
| 10 | 0.5 | 0.314 | 0.492 | 0.309 | 0.552 |
| 10 | 0.6 | 0.300 | 0.492 | 0.293 | 0.553 |
| 10 | 0.7 | 0.281 | 0.497 | 0.275 | 0.551 |
| 10 | 0.8 | 0.258 | 0.498 | 0.251 | 0.558 |
| 10 | 0.9 | 0.220 | 0.507 | 0.215 | 0.559 |
| 15 | 0.1 | 0.328 | 0.503 | 0.318 | 0.548 |
| 15 | 0.2 | 0.320 | 0.498 | 0.306 | 0.550 |
| 15 | 0.3 | 0.309 | 0.499 | 0.297 | 0.558 |
| 15 | 0.4 | 0.295 | 0.503 | 0.284 | 0.555 |
| 15 | 0.5 | 0.282 | 0.506 | 0.273 | 0.562 |
| 15 | 0.6 | 0.266 | 0.506 | 0.256 | 0.552 |
| 15 | 0.7 | 0.245 | 0.506 | 0.236 | 0.545 |
| 15 | 0.8 | 0.221 | 0.516 | 0.212 | 0.552 |
| 15 | 0.9 | 0.182 | 0.520 | 0.178 | 0.556 |
| 20 | 0.1 | 0.309 | 0.505 | 0.293 | 0.552 |
| 20 | 0.2 | 0.298 | 0.507 | 0.283 | 0.554 |
| 20 | 0.3 | 0.286 | 0.511 | 0.271 | 0.550 |
| 20 | 0.4 | 0.273 | 0.507 | 0.259 | 0.553 |
| 20 | 0.5 | 0.261 | 0.519 | 0.247 | 0.554 |
| 20 | 0.6 | 0.242 | 0.519 | 0.229 | 0.556 |
| 20 | 0.7 | 0.223 | 0.522 | 0.210 | 0.562 |
| 20 | 0.8 | 0.196 | 0.523 | 0.188 | 0.555 |
| 20 | 0.9 | 0.156 | 0.519 | 0.153 | 0.564 |
| 25 | 0.1 | 0.293 | 0.516 | 0.276 | 0.552 |
| **25** | **0.2** | **0.282** | **0.508** | 0.264 | 0.555 |
| 25 | 0.3 | 0.269 | 0.522 | 0.253 | 0.560 |
| **25** | **0.4** | 0.255 | 0.519 | **0.240** | **0.552** |
| 25 | 0.5 | 0.240 | 0.527 | 0.226 | 0.560 |
| 25 | 0.6 | 0.223 | 0.523 | 0.212 | 0.557 |
| 25 | 0.7 | 0.203 | 0.528 | 0.194 | 0.563 |
| 25 | 0.8 | 0.176 | 0.529 | 0.169 | 0.566 |
| 25 | 0.9 | 0.140 | 0.538 | 0.137 | 0.567 |
| 30 | 0.1 | 0.279 | 0.522 | 0.263 | 0.552 |
| 30 | 0.2 | 0.266 | 0.530 | 0.249 | 0.563 |
| 30 | 0.3 | 0.254 | 0.527 | 0.237 | 0.560 |
| 30 | 0.4 | 0.241 | 0.529 | 0.227 | 0.563 |
| 30 | 0.5 | 0.226 | 0.531 | 0.212 | 0.563 |
| 30 | 0.6 | 0.209 | 0.532 | 0.198 | 0.561 |
| 30 | 0.7 | 0.189 | 0.543 | 0.179 | 0.571 |
| 30 | 0.8 | 0.163 | 0.538 | 0.156 | 0.564 |
| 30 | 0.9 | 0.126 | 0.535 | 0.123 | 0.575 |
| 35 | 0.1 | 0.269 | 0.534 | 0.252 | 0.563 |
| 35 | 0.2 | 0.254 | 0.531 | 0.238 | 0.562 |
| 35 | 0.3 | 0.242 | 0.529 | 0.226 | 0.560 |
| 35 | 0.4 | 0.230 | 0.529 | 0.214 | 0.562 |
| 35 | 0.5 | 0.214 | 0.543 | 0.200 | 0.562 |
| 35 | 0.6 | 0.197 | 0.540 | 0.185 | 0.562 |
| 35 | 0.7 | 0.175 | 0.543 | 0.167 | 0.574 |
| 35 | 0.8 | 0.149 | 0.543 | 0.145 | 0.564 |
| 35 | 0.9 | 0.115 | 0.538 | 0.113 | 0.571 |
| 40 | 0.1 | 0.256 | 0.533 | 0.240 | 0.555 |
| 40 | 0.2 | 0.244 | 0.539 | 0.229 | 0.555 |
| 40 | 0.3 | 0.232 | 0.541 | 0.216 | 0.566 |
| 40 | 0.4 | 0.219 | 0.543 | 0.205 | 0.559 |
| 40 | 0.5 | 0.203 | 0.551 | 0.191 | 0.567 |
| 40 | 0.6 | 0.186 | 0.546 | 0.176 | 0.573 |
| 40 | 0.7 | 0.166 | 0.549 | 0.158 | 0.569 |
| 40 | 0.8 | 0.140 | 0.542 | 0.135 | 0.574 |
| 40 | 0.9 | 0.106 | 0.544 | 0.104 | 0.573 |
| 45 | 0.1 | 0.251 | 0.546 | 0.231 | 0.560 |
| 45 | 0.2 | 0.237 | 0.548 | 0.219 | 0.565 |
| 45 | 0.3 | 0.223 | 0.543 | 0.208 | 0.562 |
| 45 | 0.4 | 0.208 | 0.554 | 0.195 | 0.572 |
| 45 | 0.5 | 0.193 | 0.545 | 0.181 | 0.570 |
| 45 | 0.6 | 0.179 | 0.552 | 0.166 | 0.570 |
| 45 | 0.7 | 0.157 | 0.547 | 0.149 | 0.576 |
| 45 | 0.8 | 0.132 | 0.551 | 0.127 | 0.575 |
| 45 | 0.9 | 0.099 | 0.551 | 0.098 | 0.579 |
| 50 | 0.1 | 0.242 | 0.548 | 0.224 | 0.562 |
| 50 | 0.2 | 0.229 | 0.544 | 0.212 | 0.563 |
| 50 | 0.3 | 0.215 | 0.550 | 0.200 | 0.570 |
| 50 | 0.4 | 0.200 | 0.557 | 0.187 | 0.570 |
| 50 | 0.5 | 0.187 | 0.553 | 0.174 | 0.572 |
| 50 | 0.6 | 0.169 | 0.561 | 0.160 | 0.580 |
| 50 | 0.7 | 0.150 | 0.554 | 0.143 | 0.577 |
| 50 | 0.8 | 0.125 | 0.555 | 0.121 | 0.576 |
| 50 | 0.9 | 0.092 | 0.554 | 0.092 | 0.581 |

**^a^** Values for final models limited to m >= 25 ^b^ Values for final models limited to α ∈ {0.2,0.3,…,0.8} ^c^ Values are the average RMSE for training folds in 5-fold cross validation ^d^ Values are the average RMSE for validation folds in 5-fold cross validation

**Table S4:** Summary of model performance metrics for models presented in the main text or alternative modeling methods.^a^

|  |  | **Semi-stratified^b^** | | | | **Non-stratified^c^** | | | | **Semi-stratified + Concentration as Descriptor^d^** | | | |
| --- | --- | --- | --- | --- | --- | --- | --- | --- | --- | --- | --- | --- | --- |
|  | **Data Split** | **m** | **α** | **Train RMSE** | **Test RMSE** | **m** | **α** | **Train RMSE** | **Test RMSE** | **m** | **α** | **Train RMSE** | **Test RMSE** |
| **G′ Models** | 1 | 25 | 0.2 | 0.300 | 0.340 | 25 | 0.5 | 0.256 | 0.397 | 25 | 0.2 | 0.300 | 0.334 |
|  | 2 | 25 | 0.3 | 0.277 | 0.523 | 25 | 0.8 | 0.168 | 0.554 | 30 | 0.5 | 0.251 | 0.541 |
|  | 3 | 25 | 0.3 | 0.278 | 0.431 | 25 | 0.2 | 0.286 | 0.513 | 25 | 0.4 | 0.276 | 0.438 |
|  | 4 | 25 | 0.2 | 0.281 | 0.560 | 30 | 0.2 | 0.279 | 0.553 | 25 | 0.2 | 0.271 | 0.570 |
|  | 5 | 25 | 0.2 | 0.301 | 0.269 | 25 | 0.2 | 0.260 | 0.513 | 25 | 0.4 | 0.283 | 0.320 |
|  | 6 | 25 | 0.2 | 0.279 | 0.504 | 25 | 0.3 | 0.281 | 0.365 | 25 | 0.7 | 0.229 | 0.550 |
|  | **AVG** |  |  | **0.286** | **0.438** |  |  | **0.255** | **0.483** |  |  | **0.268** | **0.459** |
| **G″ Models** | 1 | 30 | 0.2 | 0.245 | 0.566 | 25 | 0.3 | 0.284 | 0.371 | 40 | 0.4 | 0.263 | 0.566 |
|  | 2 | 25 | 0.6 | 0.218 | 0.509 | 25 | 0.6 | 0.201 | 0.541 | 45 | 0.6 | 0.236 | 0.504 |
|  | 3 | 30 | 0.2 | 0.263 | 0.431 | 25 | 0.3 | 0.249 | 0.595 | 30 | 0.2 | 0.307 | 0.418 |
|  | 4 | 45 | 0.2 | 0.224 | 0.638 | 25 | 0.5 | 0.227 | 0.581 | 40 | 0.2 | 0.259 | 0.606 |
|  | 5 | 25 | 0.4 | 0.263 | 0.259 | 25 | 0.4 | 0.251 | 0.400 | 35 | 0.8 | 0.232 | 0.231 |
|  | 6 | 35 | 0.5 | 0.216 | 0.403 | 40 | 0.6 | 0.196 | 0.402 | 40 | 0.6 | 0.248 | 0.451 |
|  | **AVG** |  |  | **0.238** | **0.468** |  |  | **0.235** | **0.482** |  |  | **0.258** | **0.463** |

^a^ Presented models use cross validation of training set examples to tune hyperparameters. **^b^**Splitting method for models presented in the main text. ^c^ Splitting method does not place LMWG with highest and lowest G′/G″ in the training set by default. ^d^ Concentration was added as a descriptor for each example, other descriptors were not scaled by concentration. Otherwise identical to models presented in the main text.

**Table S5:** Summary of model performance metrics for models produced by method 3 as described in **Supplementary Note 3**. Hyperparameters in this model were tuned using a holdout validation set.

|  | **Data Split** | **m** | **α** | **Train RMSE** | **Validation RMSE** | **Test RMSE** |
| --- | --- | --- | --- | --- | --- | --- |
| **G′ Models** | 1 | 40 | 0.8 | 0.164 | 0.417 | 0.203 |
|  | 2 | 25 | 0.2 | 0.291 | 0.342 | 0.470 |
|  | 3 | 25 | 0.2 | 0.274 | 0.720 | 0.329 |
|  | 4 | 45 | 0.8 | 0.137 | 0.411 | 0.944 |
|  | 5 | 25 | 0.8 | 0.193 | 0.593 | 0.384 |
|  | 6 | 35 | 0.2 | 0.263 | 0.446 | 0.623 |
|  | 7 | 35 | 0.6 | 0.232 | 0.150 | 0.462 |
|  | 8 | 25 | 0.2 | 0.286 | 0.652 | 0.176 |
|  | 9 | 50 | 0.2 | 0.216 | 0.433 | 0.687 |
|  | 10 | 30 | 0.8 | 0.173 | 0.171 | 0.486 |
|  | **AVG** |  |  | **0.223** | **0.433** | **0.476** |
| **G″ Models** | 1 | 30 | 0.3 | 0.235 | 0.681 | 0.383 |
|  | 2 | 30 | 0.4 | 0.212 | 0.407 | 0.708 |
|  | 3 | 45 | 0.8 | 0.124 | 0.570 | 0.408 |
|  | 4 | 25 | 0.7 | 0.204 | 0.357 | 0.614 |
|  | 5 | 30 | 0.3 | 0.246 | 0.485 | 0.369 |
|  | 6 | 25 | 0.2 | 0.260 | 0.568 | 0.558 |
|  | 7 | 40 | 0.4 | 0.222 | 0.265 | 0.613 |
|  | 8 | 30 | 0.3 | 0.261 | 0.266 | 0.265 |
|  | 9 | 25 | 0.3 | 0.267 | 0.464 | 0.272 |
|  | 10 | 25 | 0.3 | 0.261 | 0.309 | 0.441 |
|  | **AVG** |  |  | **0.229** | **0.437** | **0.463** |

**Table S6:** Table of all descriptors, corresponding SMARTS string (where applicable) and mean, absolute SHAP values for the G′ and G″ model.

| Descriptor | SMARTS | G′ SHAP | G″ SHAP |
| --- | --- | --- | --- |
| ECFP_1 | *-[#6@@H](-*)-* | 0.035 | 0.036 |
| ECFP_9 | *:[#6](-[#6](=[#8])-[#6](:*):*):* | 0.000 | 0.000 |
| ECFP_25 | *-[#6]1:[#6]:[#6]:[#6](:[#6]:*:1)-[#6]=* | 0.002 | 0.003 |
| ECFP_41 | [#8]=[#6](-[#7]-[#0@@H])-[#6]-* | 0.020 | 0.023 |
| ECFP_45 | *-[#7](:[#6](:[#6]:*):[#6](:*):*):* | 0.000 | 0.002 |
| ECFP_46 | *:[#6]:[#6](-[#6](=[#8])-[#7]-[#6](-*)-*):[#6](:*):* | 0.001 | 0.001 |
| ECFP_59 | *-[#8]-[#6]1:[#6](-[#35]):[#6](:[#6]:*):[#6](:*):*:[#6]:1 | 0.002 | 0.006 |
| ECFP_79 | *-[#6@@H](-[#6]-[#6](:*):*)-* | 0.009 | 0.023 |
| ECFP_94 | *-[#8]-[#6]1:[#6](-*):*:[#6](:*):[#6]:[#6]:1 | 0.001 | 0.007 |
| ECFP_102 | *-[#6]-[#8]-[#6]1:[#6]:[#6]:*:[#6]:[#6]:1 | 0.002 | 0.001 |
| ECFP_106 | *1:[#6]:[#6]:[#6](-[#6](-[#8])=[#8]):[#6]:[#6]:1 | 0.001 | 0.001 |
| ECFP_117 | *=[#6](-[#7]-[#6@@H](-*)-*)-* | 0.023 | 0.020 |
| ECFP_147 | *-[#6](-[#8]-[#6]-*)=* | 0.017 | 0.007 |
| ECFP_173 | [#8]=[#6](-[#7]-[#6]-*)-[#6@H](-[#6]-*)-[#7]-* | 0.009 | 0.006 |
| ECFP_192 | [#8]=[#6](-[#7]-[#0@@H])-[#6]-[#8]-[#6](:*):* | 0.016 | 0.010 |
| ECFP_197 | *=[#6](-[#7]-[#6]-*)-[#0@H] | 0.003 | 0.006 |
| ECFP_203 | *:[#6]:[#6]1:[#6]:[#6]:[#6](-*):*:[#6]:1:* | 0.003 | 0.013 |
| ECFP_221 | [#8]=[#6]1:[#8]:*:[#6](:*):[#6]:[#6]:1-[#8]-[#6]-* | 0.003 | 0.005 |
| ECFP_249 | *-[#6](:*):[#6](:[#6]:*)-[#8]-[#6] | 0.000 | 0.002 |
| ECFP_265 | *:[#6]:[#6](-[#6](-[#7]-[#6]-*)=[#8]):[#6]:* | 0.001 | 0.003 |
| ECFP_283 | *-[#6@@H](-[#6])-* | 0.004 | 0.016 |
| ECFP_294 | [#6](-[#0@@H])-[#6] | 0.002 | 0.018 |
| ECFP_303 | *1:[#6]:[#6]:[#6](-[#17]):[#6](:[#6]:*):[#6]:1:* | 0.000 | 0.000 |
| ECFP_305 | [#8]=[#6]1:[#8]:[#6](:[#6]:*):[#6](:*):*:[#6]:1-* | 0.000 | 0.000 |
| ECFP_310 | *1:[#6](-[#6]-[#6]-[#6]-[#6]-1):* | 0.000 | 0.001 |
| ECFP_322 | *-[#8]-[#6](:[#6]:*):[#6]:* | 0.006 | 0.006 |
| ECFP_366 | *:[#6](:[#6](-[#17]):[#6]:*):* | 0.001 | 0.003 |
| ECFP_442 | *:[#6]1:*:[#6]:[#6](-[#35]):[#6]:[#6]:1:[#6]:* | 0.001 | 0.001 |
| ECFP_486 | *:[#6](-[#6](=[#8])-[#7]-*):* | 0.001 | 0.003 |
| ECFP_507 | *-[#7]-[#6@@H](-[#6](-[#6])-[#6])-[#6](-*)=* | 0.012 | 0.009 |
| ECFP_572 | *=[#6](-[#7]-[#6@@H](-[#6@@H](-[#6]-*)-[#6])-[#6](-[#7]-[#0@@H])=[#8])-* | 0.002 | 0.008 |
| ECFP_573 | *-[#6@@H](-*)-[#6](-[#7]-[#0@@H])=[#8] | 0.033 | 0.018 |
| ECFP_598 | *1:[#6]:[#6]2:[#6]:*:[#6]:[#6]:[#6]:2:[#6]:[#6]:1 | 0.000 | 0.011 |
| ECFP_628 | *-[#6]-[#8]-[#6]1:[#6]:[#6](:*):*:[#6](-*):[#6]:1 | 0.002 | 0.002 |
| ECFP_680 | *-[#6@H](-[#6]-[#6](-*)-*)-* | 0.007 | 0.003 |
| ECFP_684 | [#6]-[#6@@H](-[#6](-[#7]-[#6]-*)=[#8])-[#7]-* | 0.003 | 0.003 |
| ECFP_718 | *-[#8]-[#6]1:[#6]:*:[#6](:*):[#6]:[#6]:1 | 0.007 | 0.013 |
| ECFP_726 | [#8]=[#6](-[#7]-*)-[#6@H](-[#6]-[#6](-*)-*)-[#7]-[#6](-*)=* | 0.007 | 0.004 |
| ECFP_728 | *-[#35] | 0.002 | 0.005 |
| ECFP_736 | *-[#7]-[#6@@H](-[#6]-*)-[#6](-*)=* | 0.004 | 0.029 |
| ECFP_781 | *:[#6]:[#6](-[#8]-[#6]):[#6]:* | 0.005 | 0.024 |
| ECFP_802 | *-[#6]-[#7](:[#6](:*):*):[#6](:*):* | 0.007 | 0.003 |
| ECFP_835 | *-[#8]-[#6]1:[#6]:[#6](:[#6]:*):[#6](:*):*:[#6]:1 | 0.003 | 0.008 |
| ECFP_841 | *-[#8]-[#6] | 0.001 | 0.035 |
| ECFP_851 | [#6](-[#0@@H])(-[#7]-[#6@@H](-[#6]-[#6](:*):*)-[#6](-[#8])=[#8])=* | 0.006 | 0.009 |
| ECFP_875 | *-[#6](:[#6]:[#6](:*):*):* | 0.004 | 0.004 |
| ECFP_926 | *-[#6]-* | 0.003 | 0.009 |
| ECFP_989 | *-[#7]-[#6@@H](-[#6])-[#6](-*)=* | 0.014 | 0.003 |
| ECFP_1015 | *=[#6](-[#7]-[#6@@H](-[#6](-[#6])-[#6])-[#6](-[#7]-*)=[#8])-* | 0.016 | 0.026 |
| ECFP_1019 | *-[#6](-*)-* | 0.007 | 0.004 |
| ECFP_1039 | [#8]=[#6](-[#7]-[#6@@H](-[#0@@H])-*)-[#6]-[#7](:*):* | 0.004 | 0.007 |
| ECFP_1057 | [#6]-[#0@@H] | 0.005 | 0.015 |
| ECFP_1064 | *=[#6](-[#7]-[#6@@H](-[#6])-[#6](-[#8])=[#8])-* | 0.018 | 0.003 |
| ECFP_1087 | *:[#6]:[#6]1:[#6]:[#6]:[#6]:*:[#6]:1:* | 0.011 | 0.016 |
| ECFP_1088 | *:[#6]:[#6]:[#6]:* | 0.023 | 0.027 |
| ECFP_1104 | *1:[#6](-*):[#6]2:[#6]:*:[#6]:[#6]:[#6]:2:[#6]:[#6]:1 | 0.010 | 0.010 |
| ECFP_1110 | *-[#7]-[#6@@H](-[#6]-*)-[#6](-[#7]-[#6@@H](-*)-*)=[#8] | 0.007 | 0.019 |
| ECFP_1112 | *-[#8]-[#6]1:[#6]:*:[#6]:[#6]2:[#6]:*:[#6]:[#6]:[#6]:1:2 | 0.025 | 0.008 |
| ECFP_1118 | *-[#7]-[#6@@H](-[#6](-*)-*)-[#6](-*)=* | 0.009 | 0.004 |
| ECFP_1167 | [#8]=[#6](-[#7]-[#6@@H](-[#6])-[#6](-*)=*)-[#6]-* | 0.005 | 0.001 |
| ECFP_1187 | *=[#6](-*)-[#6]-[#8]-[#6](:[#6]:*):[#6](:*)-* | 0.008 | 0.001 |
| ECFP_1329 | *=[#6](-[#7]-[#6@@H](-[#6@@H](-[#6]-*)-[#6])-[#6](-[#8])=[#8])-* | 0.007 | 0.008 |
| ECFP_1343 | *-[#7]-[#6@@H](-[#6](-*)-*)-[#6](-[#8])=[#8] | 0.024 | 0.009 |
| ECFP_1357 | *:[#6](-*):[#6](:[#6]:*):[#6](:*):* | 0.006 | 0.011 |
| ECFP_1385 | *:[#6]:[#6](-[#6](=*)-*):[#6]:* | 0.006 | 0.000 |
| ECFP_1410 | *-[#8]-[#6]1:[#6]:[#6]:[#6]:*:[#6]:1-* | 0.014 | 0.001 |
| ECFP_1460 | *-[#7]-[#6@@H](-[#6@@H](-*)-*)-[#6](-[#7]-[#6@@H](-*)-*)=[#8] | 0.005 | 0.058 |
| ECFP_1517 | [#8]=[#6](-[#7]-[#6@@H](-[#6@@H](-*)-*)-[#6](-*)=*)-[#6]-* | 0.021 | 0.013 |
| ECFP_1530 | *-[#6@@H](-*)-[#6](-[#7]-[#6@@H](-[#6](-*)-*)-[#6](-*)=*)=[#8] | 0.009 | 0.004 |
| ECFP_1575 | [#8]=[#6](-[#7]-[#6]-[#6](-*)=*)-[#6@H](-*)-* | 0.004 | 0.011 |
| ECFP_1624 | *-[#7]-[#6@@H](-[#6]-*)-[#6](-[#8])=[#8] | 0.008 | 0.008 |
| ECFP_1665 | [#8]=[#6](-[#7]-[#0@@H])-[#6]1:[#6]:[#6]:*:[#6]:[#6]:1 | 0.002 | 0.004 |
| ECFP_1691 | *-[#6](-*)-[#6](=[#8])-[#7]-[#6](-[#6])-[#6](=*)-* | 0.018 | 0.007 |
| ECFP_1776 | [#6](-[#0@@H])(-[#7]-[#6@@H](-[#6](-[#6])-[#6])-[#6](-[#8])=[#8])=* | 0.014 | 0.005 |
| ECFP_1829 | *=[#6](-[#7]-[#6@@H](-[#6])-[#6](-[#7]-[#0@@H])=[#8])-* | 0.002 | 0.000 |
| ECFP_1844 | *-[#6@@H](-*)-[#6](-[#8])=[#8] | 0.016 | 0.013 |
| ECFP_1855 | *:[#6]:[#6](:[#6]:*):[#6](:*):* | 0.014 | 0.005 |
| ECFP_1906 | *-[#6]-[#8]-[#6]1:[#6]:[#6]:*:[#6](:*):[#6]:1:[#6]:* | 0.006 | 0.006 |
| ECFP_1933 | *-[#6]1:[#6]:[#6]2:[#6]:[#6](-*):*:[#6]:[#6]:2:[#6]:*:1 | 0.000 | 0.003 |
| ECFP_1935 | *-[#7]-[#6@@H](-[#6](-*)-*)-[#6](-[#7]-[#6]-*)=[#8] | 0.002 | 0.003 |
| ECFP_1977 | *=[#6](-*)-[#6@H](-[#6]-*)-[#7]-[#6](-[#8]-*)=[#8] | 0.087 | 0.031 |
| ECFP_2001 | [#6](-[#0@@H])(-[#7]-[#6@@H](-[#6]-[#6](-*)-*)-[#6](-[#8])=[#8])=* | 0.000 | 0.003 |
| FCFP_8 | *-[#35] | 0.002 | 0.008 |
| FCFP_224 | *-[#6]-[#8]-[#6]1:[#6]:[#6](:*):*:[#6]:[#6]:1 | 0.005 | 0.010 |
| FCFP_428 | [#6](-[#0@@H])-[#6](:[#6]:*):[#6]:* | 0.011 | 0.025 |
| FCFP_792 | [#6](-[#6@H](-[#0@@H])-*)-[#6] | 0.004 | 0.003 |
| FCFP_806 | *=[#6](-*)-[#6]-[#8]-* | 0.023 | 0.014 |
| FCFP_1304 | *-[#6]1:[#6](-[#35]):[#6]2:[#6]:[#6]:*:[#6]:[#6]:2:[#6]:*:1 | 0.002 | 0.001 |
| FCFP_1395 | *:[#6]:[#6]1:[#6]:[#6]:[#6](-*):*:[#6]:1:* | 0.015 | 0.015 |
| FCFP_1668 | *-[#6@@H](-[#6](-[#6])-[#6])-* | 0.009 | 0.029 |
| FCFP_1727 | *=[#6](-[#7]-[#6@@H](-[#6@@H](-[#6]-*)-[#6])-[#6](-[#7]-[#0@@H])=[#8])-* | 0.006 | 0.033 |
| FCFP_1907 | *=[#6](-[#7]-[#6@@H](-[#6])-[#6](-[#7]-[#0@@H])=[#8])-* | 0.004 | 0.006 |
| FCFP_1915 | *:[#6]1:[#6]:[#6]:[#6](-[#35]):[#6]:*:1 | 0.001 | 0.003 |
| nRings | - | 0.016 | 0.020 |

**Table S7:** Summary of tuned model metrics using Random Forest Regression as the ML algorithm

|  | **G′ Model RMSE** | | **G″ Model RMSE** | |
| --- | --- | --- | --- | --- |
| Data Split | Training Set | Testing Set | Training Set | Testing Set |
| 0 | 0.331 | 0.285 | 0.305902 | 0.591857 |
| 1 | 0.231 | 0.586 | 0.317363 | 0.525866 |
| 2 | 0.322 | 0.421 | 0.34211 | 0.356147 |
| 3 | 0.305 | 0.591 | 0.169769 | 0.626281 |
| 4 | 0.323 | 0.244 | 0.336849 | 0.316619 |
| 5 | 0.301 | 0.493 | 0.240301 | 0.369294 |
| **AVG** | **0.302** | **0.437** | **0.285** | **0.464** |

**Table S8:** Measured and predicted G′ and G″ values for 7MeO2NapFF (**36**) and analogous naphthalene compound **3** (2NapFF)

| **Cmpd** | **Conc** | **G′** | **G′ Pred** | **G′ Lower CI^a^** | **G′ Upper CI^a^** | **G″** | **G″**  **Pred** | **G″**  **Lower CI** | **G″**  **Upper CI** |
| --- | --- | --- | --- | --- | --- | --- | --- | --- | --- |
| **36** | 2.5 | 3.70 ±0.04 | 4.00 | 3.74 | 4.29 | 2.86 ±0.08 | 3.05 | 2.58 | 3.45 |
| **36** | 5 | 4.09 ±0.05 | 4.39 | 4.06 | 4.63 | 3.22 ±0.04 | 3.41 | 2.84 | 3.94 |
| **36** | 10 | 4.43 ±0.04 | 4.84 | 4.35 | 5.19 | 3.61 ±0.02 | 4.07 | 3.32 | 4.74 |
| **3** | 2.5 | 4.28 ±0.08 | 4.04 | 3.82 | 4.26 | 3.89 ±0.27 | 3.26 | 2.96 | 3.50 |
| **3** | 5 | 4.61 ±0.06 | 4.44 | 4.16 | 4.71 | 4.07 ±0.05 | 3.74 | 3.35 | 4.09 |
| **3** | 10 | 5.23 ±0.01 | 4.90 | 4.58 | 5.22 | 4.72 ±0.03 | 4.49 | 4.02 | 4.92 |

^a^ Confidence interval

# References

[1] P. Ravarino, S. Panja, S. Bianco, T. Koev, M. Wallace, D. J. Adams, *Angew. Chemie Int. Ed.* **2023**, *62*, e202215813.

[2] L. Chen, S. Revel, K. Morris, L. C. Serpell, D. J. Adams, *Langmuir* **2010**, *26*, 13466.

[3] D. J. Adams, L. M. Mullen, M. Berta, L. Chen, W. J. Frith, *Soft Matter* **2010**, *6*, 1971.

[4] E. R. Draper, E. G. B. Eden, T. O. McDonald, D. J. Adams, *Nat. Chem.* **2015**, *7*, 848.

[5] C. Patterson, B. Dietrich, C. Wilson, A. R. Mount, D. J. Adams, *Soft Matter* **2022**, *18*, 1064.

[6] P. S. Kubiak, S. Awhida, C. Hotchen, W. Deng, B. Alston, T. O. McDonald, D. J. Adams, P. J. Cameron, *Chem. Commun.* **2015**, *51*, 10427.

[7] M. C. Nolan, A. M. Fuentes Caparrós, B. Dietrich, M. Barrow, E. R. Cross, M. Bleuel, S. M. King, D. J. Adams, *Soft Matter* **2017**, *13*, 8426.

[8] A. M. Fuentes-Caparrós, F. de Paula Gómez-Franco, B. Dietrich, C. Wilson, C. Brasnett, A. Seddon, D. J. Adams, *Nanoscale* **2019**, *11*, 3275.

[9] E. R. Draper, T. O. McDonald, D. J. Adams, *Chem. Commun.* **2015**, *51*, 12827.

[10] J. Raeburn, L. Chen, S. Awhida, R. C. Deller, M. Vatish, M. I. Gibson, D. J. Adams, *Soft Matter* **2015**, *11*, 3706.

[11] J. K. Gupta, D. J. Adams, N. G. Berry, *Chem. Sci.* **2016**, *7*, 4713.

[12] J. Raeburn, T. O. McDonald, D. J. Adams, *Chem. Commun.* **2012**, *48*, 9355.

[13] D. J. Adams, I. Young, *J. Polym. Sci. Part A Polym. Chem.* **2008**, *46*, 6082.

[14] D. J. Cornwell, O. J. Daubney, D. K. Smith, *J. Am. Chem. Soc.* **2015**, *137*, 15486.

[15] H. A. Chipman, E. I. George, R. E. McCulloch, *Ann. Appl. Stat.* **2010**, *4*, 266.

[16] O. A. Martin, R. Kumar, J. Lao, *Bayesian Modeling and Computation in Python*, Chapman And Hall/CRC, Boca Raton, **2021**.

[17] C. J. Carlson, *Methods Ecol. Evol.* **2020**, *11*, 850.
